# C-terminal truncation of IFN-γ inhibits proinflammatory macrophage responses and is deficient in autoimmune disease

**DOI:** 10.1038/s41467-018-04717-4

**Published:** 2018-06-20

**Authors:** Antoine Dufour, Caroline L. Bellac, Ulrich Eckhard, Nestor Solis, Theo Klein, Reinhild Kappelhoff, Nikolaus Fortelny, Parker Jobin, Jacob Rozmus, Jennifer Mark, Paul Pavlidis, Vincent Dive, Sean J. Barbour, Christopher M. Overall

**Affiliations:** 1 Department of Oral Biological and Medical Sciences, Faculty of Dentistry, University of British Columbia, 4.401-2350 Health Sciences Mall, Vancouver, V6T 1Z3 BC Canada; 2 Centre for Blood Research, 4.401-2350 Health Sciences Mall, Vancouver, V6T 1Z3 BC Canada; 3 Department of Biochemistry and Molecular Biology, University of British Columbia, 4.401-2350 Health Sciences Mall, Vancouver, V6T 1Z3 BC Canada; 4 Department of Pediatrics, Child and Family Research Institute and BC Children’s Hospital, University of British Columbia, 3110A-950 West 28th Av, Vancouver, V5Z 4H4 BC Canada; 5 Centre for High Throughput Biology, University of British Columbia, 2125 East Mall, Vancouver, V6T 1Z3 BC Canada; 6 Department of Psychiatry, University of British Columbia, 2125 East Mall, Vancouver, V6T 1Z3 BC Canada; 7 Commissariat a l’Energie Atomique (CEA) CE-Saclay, Labex LERMIT, Service d’Ingenierie Moleculaire des Proteines, Bat 152, Gif/Yvette, 91191 France; 8 Department of Medicine, University of British Columbia, 2775 Laurel St, Vancouver, V6T 1Z3 BC Canada; 9 Present Address: Department of Physiology and Pharmacology McCaig Institute for Bone and Joint Health, Cumming School of Medicine, HRIC 3C64 3330 Hospital, Dr NW Calgary, T2N 4N1 AB Canada; 10 Present Address: Swissmedic, Swiss Agency for Therapeutics Products, Hallerstrasse 7, P.O. Box, Bern 9, CH-3000 Switzerland

## Abstract

Controlled macrophage differentiation and activation in the initiation and resolution of inflammation is crucial for averting progression to chronic inflammatory and autoimmune diseases. Here we show a negative feedback mechanism for proinflammatory IFN-γ activation of macrophages driven by macrophage-associated matrix metalloproteinase 12 (MMP12). Through C-terminal truncation of IFN-γ at 135Glu↓Leu136 the IFN-γ receptor-binding site was efficiently removed thereby reducing JAK-STAT1 signaling and IFN-γ activation of proinflammatory macrophages. In acute peritonitis this signature was absent in *Mmp12*^*–/–*^ mice and recapitulated in *Mmp12*^*+/+*^ mice treated with a MMP12-specific inhibitor. Similarly, loss-of-MMP12 increases IFN-γ–dependent proinflammatory markers and iNOS^+^/MHC class II^+^ macrophage accumulation with worse lymphadenopathy, arthritic synovitis and lupus glomerulonephritis. In active human systemic lupus erythematosus, MMP12 levels were lower and IFN-γ higher compared to treated patients or healthy individuals. Hence, macrophage proteolytic truncation of IFN-γ attenuates classical activation of macrophages as a prelude for resolving inflammation.

## Abstract (web summary)

IFN-γ is central in inflammatory pathogenesis, response to infection and autoimmune diseases. Here the authors show that MMP12 expression is reduced in patients with SLE and that MMP12 post-translationally truncates IFN-y, inhibiting its function and affecting pathogenesis of mouse models of peritonitis, SLE and rheumatoid arthritis.

## Introduction

In the initiation and resolution of inflammation the transition of macrophage populations from proinflammatory to immunosuppressive is orchestrated by coordinated multiple stimuli^1, 2^. Proinflammatory macrophages are classically activated by interferon-γ (IFN-γ) secreted from type 1 T helper (Th1) cells and natural killer (NK) cells, which also blocks Th2 cell proliferation^3–5^. IFN-γ receptor (IFNGR) engagement rapidly induces janus kinase (JAK)-signal transducer and activator of transcription 1 (STAT1) phosphorylation leading to IFN-γ-response gene transcription that increases phagocytosis, inducible nitric oxide synthase (iNOS) and radical oxygen species (ROS) levels^1, 4^. In the resolution of inflammation, alternatively activated immunosuppressive macrophages are induced by Th2 cytokines including interleukin 4 (IL-4), IL-13, IL-10, TGF-β, and immune complexes^3, 5^. An underappreciated regulatory mechanism of cytokines is post-translational truncation, as has been shown for IFN-α^6^ and long known for chemokines^7, 8^. Indeed, most chemokines are cleaved in their N-termini or C-termini by matrix metalloproteinases (MMPs), which can inactivate^9^, activate^10, 11^, convert to antagonists^12, 13^, or switch receptor specificity^14^ of chemokines. Thus, cytokine transcript analyses alone may misinform biological data interpretation.

The mechanisms underlying the inappropriate balance of Th1 to Th2 cells, and macrophage phenotypes in chronic inflammation and autoimmunity are incompletely understood. Perturbed upregulation of IFN-γ can lead to autoimmune diseases, such as rheumatoid arthritis, lupus nephritis, and systemic lupus erythematosus (SLE), in which elevated Th1 versus Th2 cell populations increase IFN-γ levels and the activation and number of destructive proinflammatory macrophages^15–18^. In a mouse genetic knockout of macrophage-associated MMP12 (metalloelastase)^19, 20^ the balance between Th1 and Th2-induced macrophage populations was swayed toward a Th1 signature in chronic experimental autoimmune encephalomyelitis^20^. This switch was associated with higher levels of IFN-γ activity, but the mechanism was unknown.

MMP12 cleaves substrates important for macrophage migration^21, 22^, for example, elastin, fibronectin, laminin, entactin, type I and IV collagens, and proteoglycan core proteins^22–24^. *Mmp12*^*–/–*^ mice are relatively healthy and, consistent with the extracellular matrix substrates of MMP12, have a defect in elastinolytic activity and a reduction in macrophage invasion to penetrate basement membranes, and a decrease in IL-13^22, 25^. MMP12 also has antimicrobial activity^26^ and a function in antiviral immunity by cleavage of IFN-α, but not IFN-β^6^, and regulates neutrophil influx via chemokine processing^23, 27^.

Conflicting reports suggest IFN-γ has dichotomous time-dependent activity and actions in autoimmunity that is not well understood^4, 16^. We hypothesized that MMP12 may contribute to the temporal regulation of IFN-γ activity by proteolytic processing. Here we show an inverse correlation between mRNA levels of IFN-γ and macrophage MMP12 in SLE patients and upon treatment. We characterize inactivation of IFN-γ by C-terminal proteolytic processing that contributes to attenuation of proinflammatory macrophage activation in acute inflammation. This negative feedback mechanism is driven by MMP12 secretion by proinflammatory IFN-γ-activated macrophages, with elevated MMP12 expression in IL-4-activated immunosuppressive macrophages reinforcing inflammation resolution. Contrary to the detrimental functions classically ascribed to MMPs in inflammation, low-MMP12 levels may be a risk factor underlying excessive proinflammatory IFN-γ macrophage activation in disease.

## Results

### Negative association of MMP12 with human lupus

**Fig. 1:**
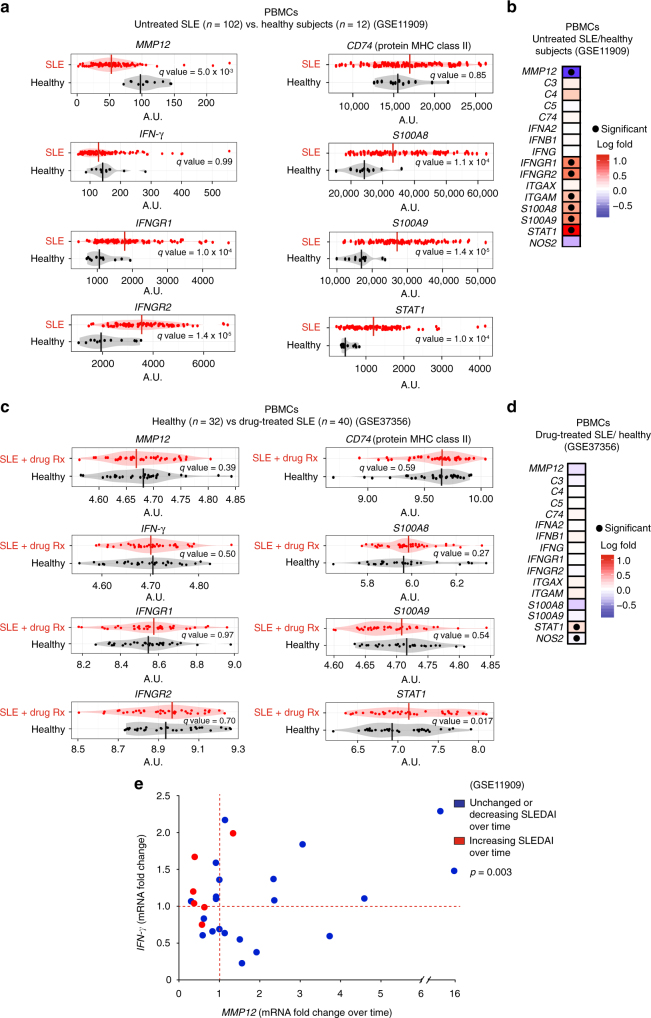
Human PBMC MMP12 and IFN-γ response gene mRNAs in SLE. **a** PMBC mRNA levels and **b** quantification of *MMP12, C3, C4, C5, CD74, IFNA2, IFNB1, IFNG, IFNGR1, IFNGR2, ITGAM, ITGAX S100A8, S100A9, STAT1*, and *NOS2* mRNAs in SLE patients (*n* = 102) or healthy control subjects (*n* = 12) in the GSE11909 transcript dataset^28^. Vertical bars are the mean values in all bean plots. **c** Comparison of these mRNA levels in PBMCs of drug-treated SLE patients (*n* = 40) or healthy control subjects (*n* = 32) in the GSE37356 mRNA dataset^15^. Statistical significance in **b** and **d** was determined by calculating the *q-*values in **a** and in **c**. **e** Longitudinal comparison (31 time points) in 20 individual subjects of the fold changes of *MMP12* and *IFNG* PBMC mRNA levels upon clinical deterioration (increasing SLEDAI in individual patients over time, *n* = 7 time points), or upon clinical improvement (unchanged or decreasing SLEDAI in individual patients over time, *n* = 24 time points). Patient information (ethnicity, gender, age, SLEDAI and drug treatment or not) from GSE11909 is in Supplementary Tables S4-S5^28^. Statistical significance was determined by a two-tailed paired Student’s *t*-test: *p* = 3 × 10^−3^

To examine the association of MMP12 expression in human autoimmune disease, we analyzed two human SLE peripheral blood mononuclear cell (PBMC) transcriptome datasets. In the GSE11909^28^ study, untreated active SLE was associated with significantly reduced *MMP12* mRNA (*q* = 5.0 × 10^−3^) (*n* = 102) compared with healthy subjects (*n* = 12) (Fig. 1a). IFN-γ response genes were correspondingly significantly higher than in healthy controls: *IFNGR1* (*q* = 1 × 10^−4^), *IFGNR2* (*q* = 1 × 10^−5^), *ITGAM* (*q* = 3 × 10^−2^), *S100A8* (*q* = 1 × 10^−4^), *S100A9* (*q* = 1 × 10^−4^), and *STAT1* (*q* = 1 × 10^−4^) (Fig. 1b; Supplementary Fig. 1). In a different cohort of stabilized SLE patients that had been successfully treated with corticosteroid, hydroxychloroquine, and/or immunosuppressants (*n* = 40) (GSE37356)^15^, the *MMP12* levels were comparable (*q* = 0.39) to those of healthy controls (*n* = 32) (Fig. 1c), with no significant differences observed in the levels of IFN-γ response genes *IFNGR1* (*q* = 0.97), *IFGNR2* (*q* = 0.70), *ITGAM* (*q* = 0.41), *S100A8* (*q* = 0.27) and *S100A9* (*q* = 0.54), or *IFNG* (*q* = 0.50) (Fig. 1c; Supplementary Fig. 2).

Associated with increasing systemic lupus erythematosus disease activity index (SLEDAI) and clinical deterioration of SLE patients over time was increased IFN-γ associated with significantly decreased *MMP12* mRNA levels (two-tailed paired Student’s *t*-test: *p* = 6 × 10^−3^) (GSE11909, *n* = 114)^28^ (Fig. 1e, Supplementary Fig. 3). Conversely, in patients showing clinical improvement with decreasing SLEDAI over time, *MMP12* mRNA was markedly increased up to ~16-fold (two-tailed paired Student’s *t*-test: *p* = 3 × 10^−2^) (Fig. 1e, Supplementary Fig. 3). In sum, our reanalysis of human clinical sample transcript data revealed that low levels of MMP12 were associated with autoimmune disease and elevated IFN-γ signature genes. Thus, MMP12 may exert clinically significant protective roles that are deficient in the development of SLE. The return of higher MMP12 levels upon treatment and resolution of clinical symptoms are consistent with this hypothesis.

### C-terminal truncation of IFN-γ

**Fig. 2:**
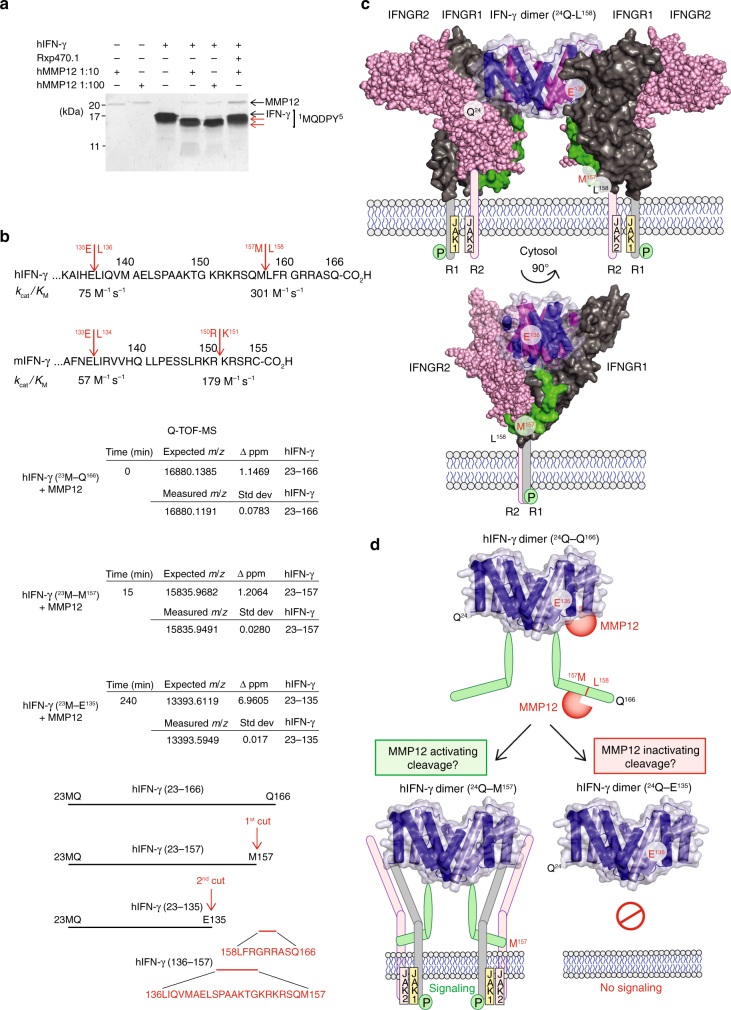
MMP12 cleaves IFN-γ removing the IFN-γ receptor-binding site. **a** Silver stained 15% SDS-PAGE analysis of in vitro cleavage of human (h) IFN-γ by 10 or 100 ng hMMP12 catalytic domain at 1:10 and 1:100 enzyme to substrate ratios, incubated over 18 h at 37 °C. Revealing C-terminal cleavage, N-terminal sequencing identified an intact N-terminus commencing at 1MQDPY both in IFN-γ and in the two cleavage products (red arrows). The MMP12-specific inhibitor, Rxp470.1, blocked IFN-γ cleavage and autocatalytic cleavage of MMP12 resulting in stabilized MMP12 protein levels over 18 h. Molecular weight marker positions are shown. **b** Q-TOF-MS analysis of IFN-γ cleavage reaction products revealed C-terminal cleavage first between 157Met↓Leu158 and then at 135Glu↓Leu136 (see Supplementary Fig. 4). The *k*_cat_/*K*_M_ values calculated for each cleavage event in human and mouse (m) IFN-γ are shown. **c** Based on the crystal structures of the IFN-γ homodimer (pdb entry: 1HIG)^33, 51^, a secondary complex consisting of the IFN-γ dimer, two high-affinity IFN-γ receptor 1 molecules (IFNGR1(pdb entry: 1FG9); 29Val-Ser241; dark gray), and two low affinity IFN-γ receptor 2 chains (IFNGR2 (pdb entry: 1FYH); 30Leu-Thr237; light pink) were modeled. The IFN-γ C-terminal peptide (135Glu–158Leu) responsible for IFN-γ receptor interaction and signaling was modeled (green). The transmembrane peptide and JAK1/2 are shown in cartoon form. **d** Frontal view of the structured region of the IFN-γ homodimer with the C-terminal non-structured flexible region (from 146Ala to Gln166) cartooned in green. The two MMP12 cleavage sites are shown: 157Met↓Leu158 and 135Glu↓Leu136

We hypothesized that MMP12 cleaves IFN-γ and attenuates classical activation of macrophages in vivo. Enzyme kinetic analyses revealed that human and mouse IFN-γ were efficiently cleaved proximal to the C-terminus by human and mouse MMP12, respectively (Fig. 2a; Supplementary Fig. 4a). IFN-γ cleavage was prevented by the MMP12-specific inhibitor, Rxp470.1^6, 29, 30^. The first cleavage product of human IFN-γ was apparent within 15 min of co-incubation, consistent with a *k*_cat_/*K*_M_ (1) of 301 M^−1^ s^−1^ (Fig. 2b). A second MMP12 cleavage rapidly followed (*k*_cat_/*K*_M_ = 75 M^−1^ s^−1^ (1)) and both products displayed an intact N-terminus (Met23 of the recombinant protein) as shown by Edman microsequencing (Fig. 2a). High-mass accuracy top down mass spectrometry identified the two cleavage sites in human IFN-γ at 157Met↓Leu158 and 135Glu↓Leu136 (Fig. 2b, Supplementary Figs. 4b and 5a–c). The 135Glu↓Leu136 scissile bond is conserved in human, monkey, mouse, and rat (N/HE↓LIR/QV) suggestive of a conserved function (Supplementary Fig. 4b). In contrast, MMP12 did not cleave the immunosuppressive macrophage alternative activator, IL-4 (Supplementary Fig. 6).

IFNGRs are comprising two ligand binding IFNGR1 chains and two signal-transducing IFNGR2 chains^3, 31^. We related the cleavage sites in the IFN-γ dimer to the IFNGR chain structures using in silico structural analysis (Fig. 2c). The IFN-γ non-structured C-terminal strand was modeled from Ala146 since it is absent from the crystal structure^32, 33^. Our analysis predicted that the strand remaining after cleavage at 157Met↓Leu158 would retain binding to both homodimers of IFNGR1 and IFNGR2, consistent with activation^34, 35^ (Fig. 2d). However, both cleavage products were consistently identified together at all time points by mass spectrometry indicating rapid processive cleavage leading to the final shorter truncated product ending at Glu135 (Fig. 2b and Supplementary Fig. 5a–c). As the loss-of-IFNGR2 binding is predicted by truncation at 135Glu↓Leu136, this suggests the net effect of MMP12 is loss-of-IFN-γ signaling.

### Reduced IFN-γ activation of macrophages by MMP12 in vivo

**Fig. 3:**
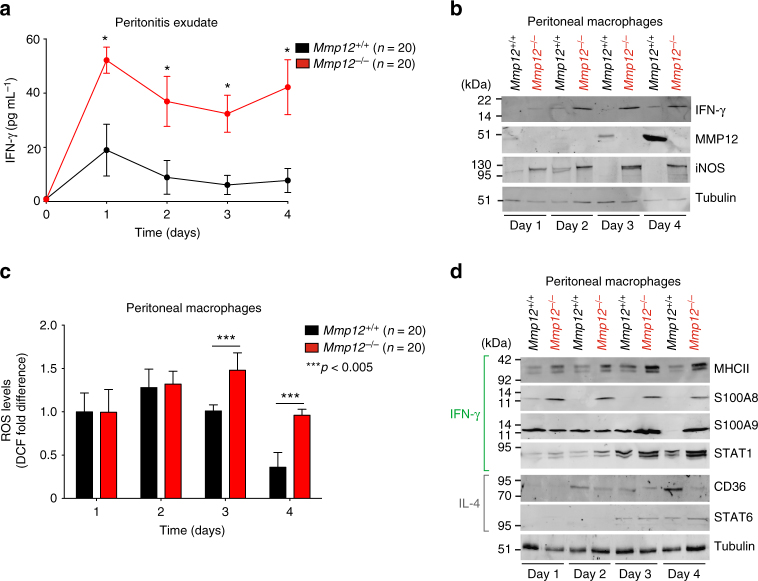
MMP12 decreases IFN-γ-activated macrophage markers in acute peritonitis. **a** ELISA of IFN-γ protein levels in peritoneal exudate of male *Mmp12*^*+/+*^ B10.RIII (*n* = 20) and *Mmp12*^*–/–*^ B10.RIII (*n* = 20) mice at days 0–4 after induction of peritonitis (*n* = 4 for each genotype for each time point, *N* = 2) expressed as the mean ± s.d. There was no IFN-γ quantified in healthy peritoneum in the absence of inflammation on day 0. Statistical significance was determined by two-tailed unpaired Student’s *t*-test: *p < 0.05. **b** 10% SDS-PAGE western blot analysis of IFN-γ, MMP12, and iNOS proteins in primary peritoneal macrophages harvested daily from *Mmp12*^*+/+*^ (*n* = 20) and *Mmp12*^*–/–*^ (*n* = 20) B10.RIII mice (*N* = 2). Tubulin, loading control. **c** Cellular ROS levels in primary peritoneal macrophages were quantified by calculating the mean fluorescence intensity after treatment with 2,-7-dichlorofluorescein diacetate (DCF) (*n* = 20 for each genotype, *n* = 4 for each time point, *N* = 2). Data were normalized to day 1 *Mmp12*^*+/+*^ B10.RIII macrophages and expressed as fold differences. Error bars, s.d. Statistical significance was determined by a two-tailed unpaired Student’s *t*-test: ***p < 0.005. **d** 10% SDS-PAGE western blot analysis of markers characteristic for macrophage activation by IFN-γ (MHCII, S100A8, and S100A9) and STAT1, or by IL-4 (CD36) and STAT6 in *Mmp12*^*+/+*^ and *Mmp12*^*–/–*^ B10.RIII mouse macrophages harvested daily (*n* = 20 for each genotype, *n* = 4 for each time point, *N* = 2). Tubulin, loading control. Molecular weight marker positions in all blots are as shown

We next examined IFN-γ levels in acute thioglycollate-induced peritonitis from 0 to 4 days in *Mmp12*^*+/+*^ B10.RIII (*n* = 20) and *Mmp12*^*–/–*^ B10.RIII (*n* = 20) mice (*n* = 4, for each genotype for each day). Consistent with the initiation of IFN-γ clearance by MMP12 in the *Mmp12*^*+/+*^ mice, ELISA analyses revealed that IFN-γ protein was ~3-fold higher in the peritonitis exudate at days 1 to 4 in the *Mmp12*^*–/–*^ mice (Fig. 3a). This difference was also found in peritoneal macrophage lysates by western blotting (Fig. 3b) and was associated with increasing amounts of MMP12 protein over time in the *Mmp12*^*+/+*^ B10.RIII mice (Fig. 3b). The IFN-γ-activated macrophage response markers^5^, iNOS (Fig. 3b) with attendant ROS production (Fig. 3c), were also up at day 2 and then later declined at days 3 and 4 in the *Mmp12*^*+/+*^ B10.RIII peritoneal macrophages. However, these levels were always greater in the *Mmp12*^*–/–*^ B10.RIII primary macrophages, with iNOS and ROS continuing to significantly increase over time; whereas, iNOS protein was undetectable in the *Mmp12*^*+/+*^ B10.RIII macrophages at days 3 and 4.

As no one marker is indicative of either proinflammatory classically activated or immunosuppressive alternatively activated macrophages, we supported the iNOS findings in peritonitis by a spectrum of other markers associated with IFN-γ activation. Thus, STAT1, MHCII, S100A8, and S100A9 were also at higher levels in primary *Mmp12*^*–/–*^ B10.RIII macrophages compared with *Mmp12*^*+/+*^ B10.RIII cells, particularly at the later time points (Fig. 3d). In contrast, *Mmp12*^*+/+*^ B10.RIII mice displayed higher levels of CD36 on day 4, which is a marker of IL-4 alternative activation. Thus, *Mmp12*^*–/–*^ B10.RIII mice displayed elevated IFN-γ protein and prolonged classical macrophage activation in acute inflammation compared with *Mmp12*^*+/+*^ B10.RIII mice. In other words, the absence of MMP12 shifted peritoneal macrophage populations to one enriched with IFN-γ-classically activated proinflammatory macrophages.

### IFN-γ cleavage reduces STAT1 and iNOS during phagocytosis

**Fig. 4:**
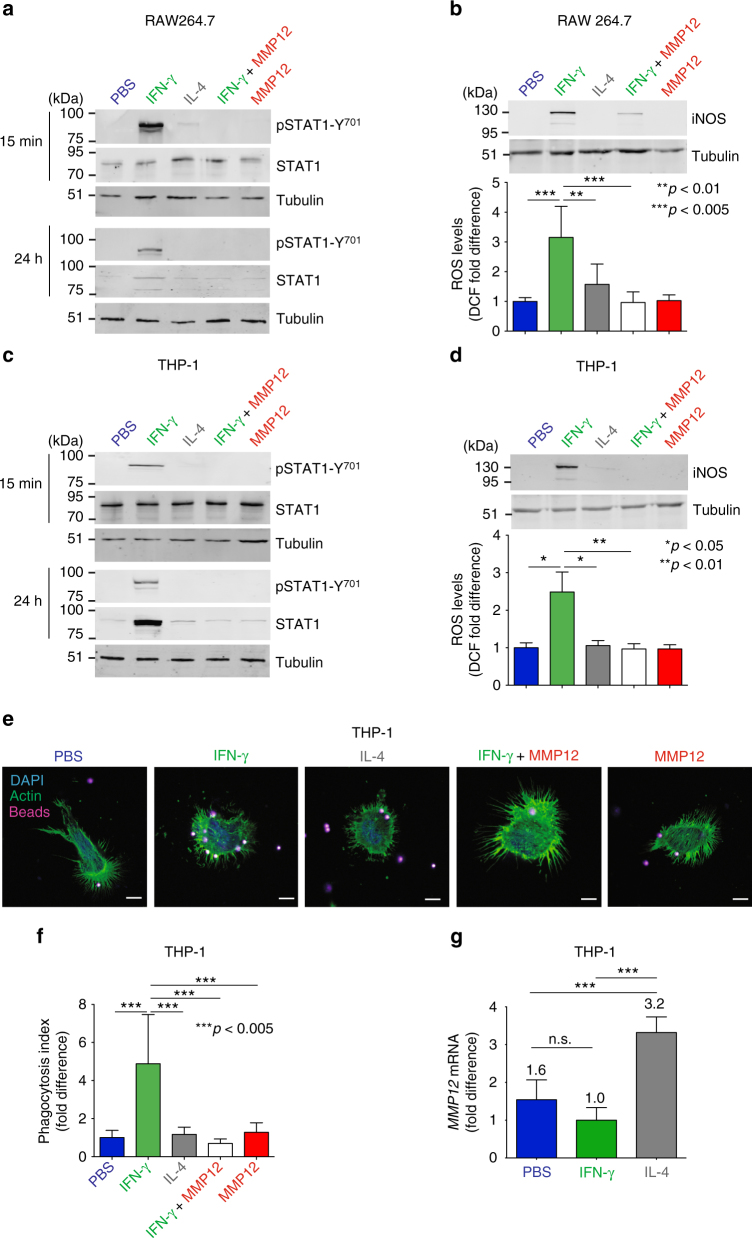
MMP12 reduces IFN-γ signaling and responses in macrophages. **a** Mouse RAW264.7 cells were treated for 15 min or 24 h with PBS, 20 ng/mL mouse IFN-γ, 20 ng/mL mouse IFN-γ pre-incubated with 2 ng/mL mouse MMP12 (37 °C, 18 h), 2 ng/mL mouse MMP12 alone, or 30 ng/mL mouse IL-4 (*n* = 4, *N* = 2). After 10% SDS-PAGE, cell lysates were western blotted for pSTAT1-Y701 and STAT1 proteins (Supplementary Fig. 7b). **b** Western blot of iNOS protein in RAW264.7 cells treated as above for 24 h (*n* = 4, *N* = 2). ROS levels were quantified by calculating the mean fluorescence intensity after treatment with 2-,7-dichlorofluorescein diacetate (DCF)(*n* = 4, *N* = 2). Data were normalized to PBS-treated cells and expressed as fold differences. Error bars denote s.d. Statistical significance was determined by a two-tailed unpaired Student’s *t*-test: *p < 0.05; **p < 0.01; ***p < 0.005. **c**, **d** Western blotting, quantification, and statistical analyses of pSTAT1-Y701, STAT1, and iNOS protein in human THP-1 cells as described for **a** and **b** (*n* = 4, *N* = 2) (see also Supplementary Fig. 7a). Tubulin and molecular weight marker positions are shown. **e** Representative images and **f** phagocytic index of THP-1 macrophages incubated for 24 h with PBS, 30 ng/mL IL-4, 20 ng/mL IFN-γ, or 20 ng/mL IFN-γ pretreated with 2 ng/mL human MMP12 (37 °C, 18 h), or 2 ng/mL MMP12 alone, and then incubated with serum-coated fluorescent 2-μm microparticles. Scale bars, 20 μm. The phagocytic index was quantified from the number of beads per cell (5–30 cells per field) in each of 20 fields (*n* = 3, *N* = 2). Data were normalized to PBS-treated THP-1 macrophages and the means expressed as fold differences. Error bars, s.d. Statistical significance was determined by a two-tailed unpaired Student’s *t*-test: ***p < 0.005. **g** *MMP12* mRNA analysis of PMA-matured THP-1 cells treated with PBS, IFN-γ, or IL-4 (*n* = 3, *N* =2). A-values were normalized to IFN-γ-induced THP-1 cell mean values and expressed as fold differences. Error bars, s.d. Statistical significance was determined by a two-tailed unpaired Student’s *t*-test: NS not significant difference; ***p = 1 × 10^−5^

IFN-γ activates JAK-STAT1 signaling with phosphorylation of STAT1 at Tyr701 leading to IFN-γ response gene transcription occurring within 15–30 min and a characteristic upregulation of total STAT1 protein expression after 18–24 h that is highly correlated with IFN-γ activation of macrophages^3, 36^. To examine the effect of MMP12 cleavage on IFN-γ signaling, we treated mouse RAW264.7 cells lines for 15 min or 24 h with full-length mouse IFN-γ, or with mouse MMP12-cleaved mouse IFN-γ terminating at Glu133 that was mass spectrometry confirmed (Fig. 4a, b). We also treated PMA-matured THP-1 human monocytes with human IFN-γ or with human MMP12-cleaved human IFN-γ terminating at Glu135 (mass spectrometry confirmed) (Fig. 4c, d). In both macrophage cell lines, IFN-γ led to the characteristic phosphorylation of STAT1 at Tyr701 after 15 min with subsequent upregulation of total STAT1 protein at 24 h (Supplementary Fig. 7a, b). Tyr701 phosphorylation was associated with an increase in iNOS protein (Fig. 4b, d). In contrast, incubation with MMP12-truncated IFN-γ neither induced STAT1 phosphorylation nor increased total STAT1 or iNOS proteins in either cell line.

An important response to macrophage classical activation by IFN-γ is elevated phagocytosis with associated induction of microbicidal activity wrought by ROS^3^ that is generated by elevated iNOS^5^. In a fluorescent bead phagocytosis assay with differentiated THP-1 cells, PBS-treated and IL-4-activated macrophages showed low levels of phagocytosis (Fig. 4e, f). Phagocytosis was increased 4.9-fold in IFN-γ-activated THP-1 cells, but when IFN-γ was cleaved by MMP12, the cells retained the lower phagocytic activity of non-activated macrophages. These functional data are consistent with the elevation in iNOS protein levels in both IFN-γ-activated RAW264.7 and THP-1 cells compared to PBS-treated or IL-4-alternately activated macrophages, as well as a corresponding increase in cellular ROS (Fig. 4b, d). In contrast, MMP12 cleavage of IFN-γ reduced the increase in the IFN-γ activation markers iNOS and ROS.

As expected, IL-4 treatment of RAW264.7 and THP-1 cells did not affect STAT1 protein phosphorylation (Fig. 4a, c), but did trigger the characteristic Tyr641 phosphorylation of STAT6^37^ (Supplementary Fig. 7c, d). Thus, IFN-γ signaling was reduced by MMP12 cleavage of IFN-γ at 135Glu↓Leu136, which in turn reduced IFN-γ-activated macrophage polarization, whereas IL-4-activated macrophage polarization was unaffected by MMP12. Finally, we analyzed *MMP12* mRNA expression in PMA-matured THP-1 cells (M_0_) that were treated with IFN-γ, IL-4, or PBS vehicle (*n* = 3, *N* = 2). PBS controls or IFN-γ-activated cells expressed *MMP12* mRNA that were measured with microarray A-values of 9.62 and 8.99, respectively, which were not significantly different (two-tailed paired Student’s *t*-test: *p* = 0.09). In contrast, IL-4-activated THP-1 cells expressed 3.2-fold more *MMP12* mRNA with an A-value of 10.73 and that was significantly different from the PBS controls and IFN-γ-activated THP-1 cells (two-tailed paired Student’s *t*-test: *p* = 1 × 10^−5^) (Fig. 4g). Hence, these cellular readouts support an IFN-γ function-inactivating cleavage by MMP12 to lessen IFN-γ-activation of macrophages, but not that of IL-4 macrophage activation. Indeed, IL-4 activation led to elevated *MMP12* expression, which may act *in trans* in mixed cell populations to further reduce IFN-γ macrophage activation.

### *Mmp12* loss is associated with elevated IFN-γ markers in arthritis

**Fig. 5:**
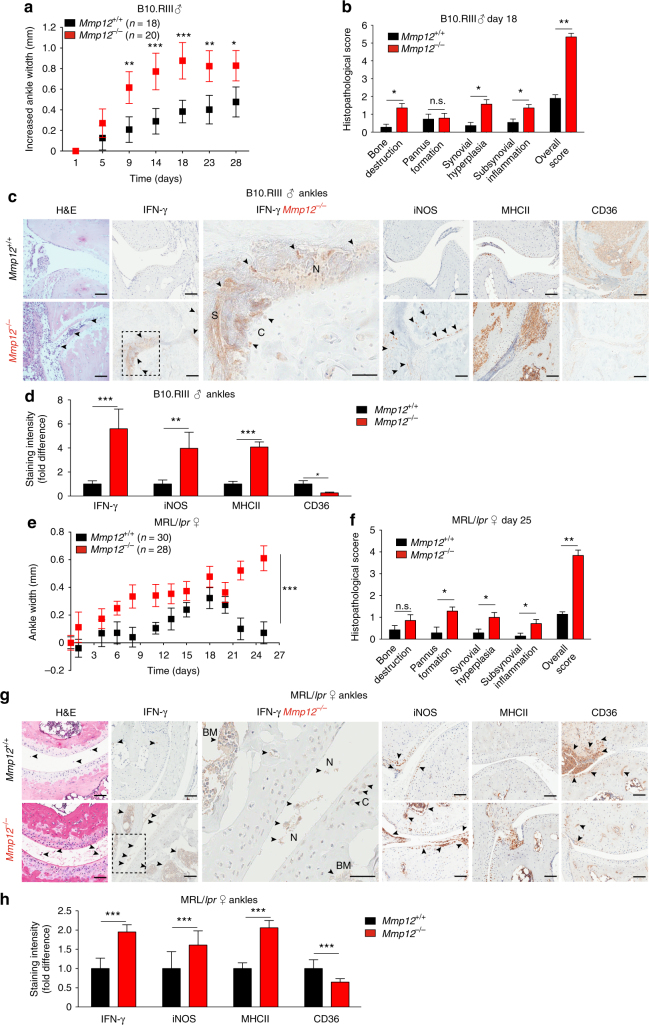
Altered macrophage markers in rheumatoid arthritis in *Mmp12*^*–/–*^ mice. **a** Hind ankle widths of *Mmp12*^*+/+*^ and *Mmp12*^*–/–*^ male B10.RIII mice (*n* = 18 and 20, respectively, for each time point) after onset of collagen-induced arthritis (day 0), means ± s.e.m. Mann–Whitney *t*-test: *p < 0.05, **p < 0.01, ***p < 0.005. **b** Histopathology of hind ankles after H&E staining (day 18) *Mmp12*^*+/+*^ (*n* = 3) and *Mmp12*^*–/–*^ (*n* = 3). Bone destruction (*p* < 6 × 10^−5^), pannus formation (NS not significant), synovial hyperplasia (*p* < 3 × 10^−5^), and subsynovial inflammation (*p* < 7 × 10^−4^) were quantified as a histopathological score (*p* < 9 × 10^−4^, means ± s.d.), two-tailed unpaired Student’s *t*-test: *p < 0.05, **p < 0.01. **c** Representative images of H&E, IFN-γ, iNOS, MHCII, and CD36 immunostaining of hind ankle joints of *Mmp12*^*+/+*^ (*n* = 3) and *Mmp12*^*–/–*^ (*n* = 3) mice. Here and in **g**: N NETs; C cartilage; S synovial space; arrowheads, high antibody or H&E staining; scale bars, 100 μm. **d** Immunostaining quantification of *Mmp12*^*+/+*^ versus *Mmp12*^*–/–*^ male B10.RIII mice hind ankle joints for IFN-γ (*p* < 2 × 10^−5^), iNOS (*p* < 1 × 10^−3^), MHCII (*p* < 2 × 10^−5^), and CD36 (*p* < 2 × 10^−2^) (means ± s.d.), two-tailed unpaired Student’s *t*-test: *p < 0.05, **p < 0.01, ***p < 0.005. **e** Ninety-day-female MRL/*lpr* mice were injected with CFA (day 0). Hind ankle size was measured in *Mmp12*^*+/+*^and *Mmp12*^*–/–*^ MRL/*lpr* mice (*n* = 30 and 28, respectively, for each time point) (means ± s.d.), two-tailed unpaired Student’s *t*-test: ***p < 0.005. **f** Histopathology of H&E stained-hind ankle joints of *Mmp12*^*+/+*^ (*n* = 3) and *Mmp12*^*–/–*^ (*n* = 3) MRL/*lpr* (day 25). Bone destruction (n.s.), pannus formation (*p* < 0.01), synovial hyperplasia (*p* < 3 × 10^−2^), and subsynovial inflammation (*p* < 3 × 10^−2^) were quantified as the histopathological score (*p* < 6 × 10^−3^) (means ± s.d.), two-tailed unpaired Student’s *t*-test: *p < 0.05, **p < 0.01. **g** Representative images of H&E, IFN-γ, iNOS, MHCII, and CD36 immunostaining of hind ankle joints of *Mmp12*^*+/+*^ and *Mmp12*^*–/–*^ MRL/*lpr* female mice. BM bone marrow. **h** Immunostaining intensities of *Mmp12*^*+/+*^ and *Mmp12*^*–/–*^ mouse hind ankle joints for IFN-γ (*p* < 2 × 10^−5^), iNOS (*p* < 1 × 10^−3^), MHCII (*p* < 4 × 10^−4^), and CD36 (*p* < 4 × 10^−4^) (means ± s.d.), two-tailed unpaired Student’s *t*-test: ***p < 0.005

We found that genetic deletion of MMP12 increased the severity of inflammatory arthritis in mice. Twenty-eight days after induction of collagen-induced arthritis, male *Mmp12*^*–/–*^ B10.RIII mice (*n* = 20) exhibited more severe arthritis (Fig. 5a, b), with increased ankle width and histopathological scores compared with male *Mmp12*^*+/+*^ B10.RIII mice (*n* = 18). The *Mmp12*^*–/–*^ B10.RIII mice also had higher levels of IFN-γ and the IFN-γ-activated macrophage markers iNOS and MHCII, with concomitant near absent CD36 staining (Fig. 5c, d). Neutrophils and neutrophil extracellular traps (NETs) in the synovial space^23^ further indicated an acute IFN-γ-driven proinflammatory response^38^.

We confirmed these findings in female mice on another background. The autoimmune MRL/*lpr* mouse strain^39^ spontaneously develops systemic autoimmunity with severe lymphadenopathy, arthritis and glomerulonephritis. In our substrain, we found it was necessary to initiate autoimmune arthritis earlier with Complete Freund’s adjuvant (CFA), otherwise gross lymphadenopathy necessitated euthanasia too soon to derive meaningful insights into the development of severe arthritis. As CFA stimulates the hyperproduction of IFN-γ, expression of MHC class II genes, and Th1/classical activation of macrophages^39, 40^ we reasoned this strain was suited to assess the effects of MMP12 on IFN-γ-driven disease and macrophages in vivo.

In female *Mmp12*^*–/–*^ MRL/*lpr* mice, we saw similar associations as found in the B10.RIII mice. Hind ankle joints at baseline were normal with no evidence of disease as quantified by a 4-point histopathological grading scheme^41^, including no difference in paw edema in both the *Mmp12*^*+/+*^ (*n* = 30) and *Mmp12*^*–/–*^ (*n* = 28) female MRL/*lpr* mice (Supplementary Fig. 8b). With loss-of-MMP12 there was an increased incidence of arthritis as compared to *Mmp12*^*+/+*^ MRL/*lpr* mice (Supplementary Fig. 8a). In the acute disease phase immediately following CFA induction, the *Mmp12*^*–/–*^ MRL/*lpr* mice displayed a faster onset of arthritis that was detectable at day 6 by significant and more severe ankle swelling than *Mmp12*^*+/+*^ MRL/*lpr* mice, that continued to exacerbate to day 25 (Fig. 5e). In contrast, in *Mmp12*^*+/+*^ MRL/*lpr* mice, ankle edema started to decrease at day 19 and continued to fall to day 25—at which time the *Mmp12*^*–/–*^ MRL/*lpr* mice showed larger pannus formation, more synovial hyperplasia and subsynovial inflammation (Supplementary Fig. 8c) with a histopathological score of 3.8 in comparison to 1.1 for *Mmp12*^*+/+*^ MRL/*lpr* mice (Fig. 5f). In quantifying differences in IFN-γ-activated macrophage proteins at day 25 we found the *Mmp12*^*–/–*^ MRL/*lpr* ankles had elevated IFN-γ, as well as elevated iNOS and MHCII classical activation marker proteins, reflecting sustained IFN-γ-stimulated macrophage responses in vivo (Fig. 5g, h). This indicated that without MMP12, MRL/*lpr* mice experienced a loss-of-control of macrophage activation and inflammation that led to a sustained disease flare.

### Elevated IFN-γ activation in Mmp12^–/–^ mouse models of lupus

**Fig. 6:**
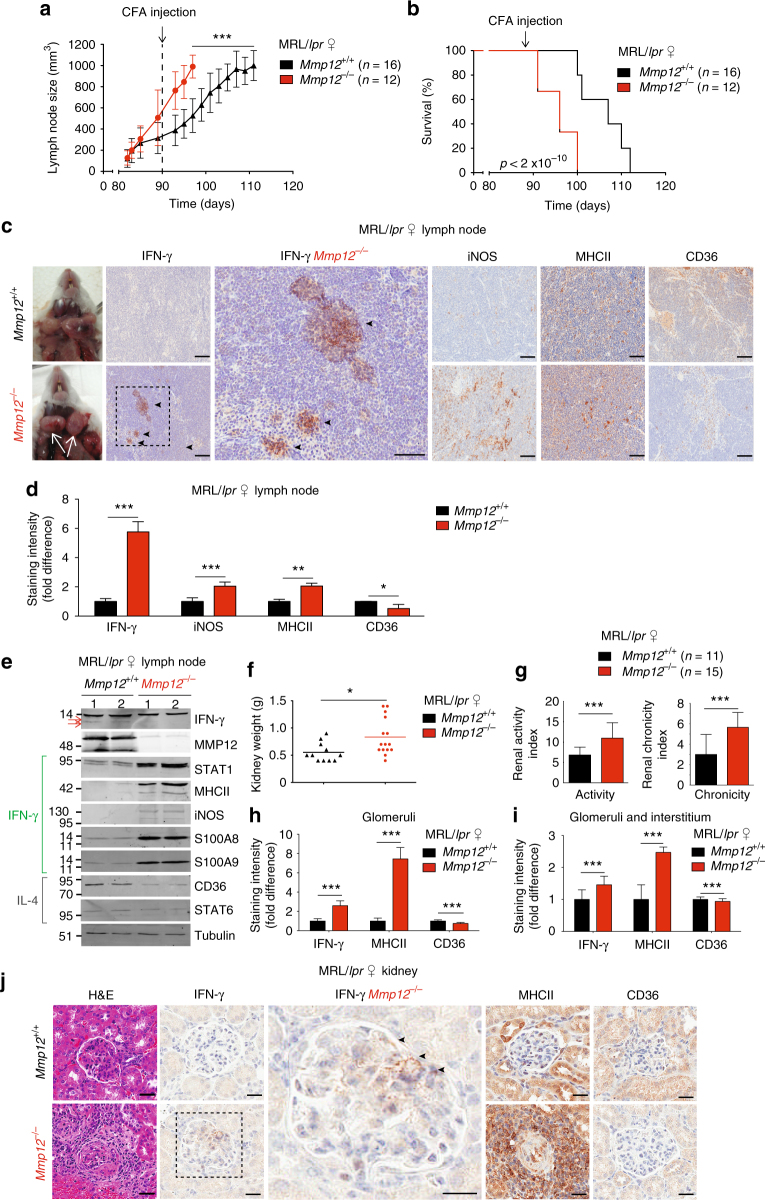
*Mmp12*^*–/–*^ mouse mortality and IFN-γ macrophages numbers in SLE. **a** Following CFA injection, the size of the superficial cervical lymph nodes of *Mmp12*^*+/+*^ and *Mmp12*^*–/–*^ MRL/*lpr* (*n* = 16 and 12, respectively, for each time point) female mice were measured and expressed as means ± s.d. Two-tailed unpaired Student’s *t*-test: ***p < 0.005. **b** Kaplan–Meier curves showing mortality rates of female *Mmp12*^*+/+*^ MRL/*lpr* (*n* = 16) and *Mmp12*^*–/–*^ (*n* = 12) mice. Two-tailed unpaired Student’s *t*-test: *p* < 2 × 10^−10^, (see Supplementary Fig. 9c, d for survival data). **c** Representative images of superficial cervical lymph nodes (white arrows) immunostained for IFN-γ, iNOS, MHCII, and CD36 from *Mmp12*^*+/+*^ (*n* = 3) and *Mmp12*^*–/–*^ (*n* = 3) female MRL/*lpr* mice at their humane end points (*Mmp12*^*+/+*^ (day 112) and *Mmp12*^*–/–*^ (day 98)). Scale bars, 100 μm. **d** Quantification of immunostaining intensities of *Mmp12*^*+/+*^ (*n* = 3) versus *Mmp12*^*–/–*^ (*n* = 3) MRL/*lpr* mice superficial cervical lymph nodes for IFN-γ (*p* < 9 × 10^−6^), iNOS (*p* < 2 × 10^−7^), MHCII (*p* < 1 × 10^−2^), and CD36 (*p* < 2 × 10^−2^) expressed as the mean ± s.d. Two-tailed unpaired Student’s *t*-test: *p < 0.05, **p < 0.01, ***p < 0.005. **e** Superficial cervical lymph nodes were analyzed by western blotting for macrophages markers of IFN-γ activation (iNOS, MHCII, S100A8, and S100A9) and STAT1, as well as for IL-4 induced CD36 and STAT6. Two biological replicates analyses from *Mmp12*^*+/+*^ (*n* = 8) and *Mmp12*^*–/–*^ (*n* = 8) MRL/*lpr* mice, are shown. Red arrows indicate lower molecular forms of IFN-γ. **f** Mean kidney weights and **g** mean histological scores of activity and chronicity indexes from *Mmp12*^*+/+*^ (*n* = 11) and *Mmp12*^*–/–*^ MRL/*lpr* (*n* = 15) mice were measured at their humane end points. Error bars, s.d. Two-tailed unpaired Student’s *t*-test: *p < 0.05, ***p < 0.005. Quantification of immunostaining in **h** glomeruli and **i** in glomeruli and interstitium of *Mmp12*^*+/+*^ (*n* = 3) and *Mmp12*^*–/–*^ MRL/*lpr* (*n* = 3) mice at their humane end points for IFN-γ (*p* < 2 × 10^−12^), MHCII (*p* < 7 × 10^−17^), and CD36 (*p* < 2 × 10^−6^). Quantification is expressed as means ± s.d. Two-tailed unpaired Student’s *t*-test: ***p < 0.005. **j** Representative images of H&E, IFN-γ, MHCII, and CD36 immunostaining of the renal glomeruli sections of *Mmp12*^*+/+*^ and *Mmp12*^*–/–*^ female MRL/*lpr* mice; arrowheads, strong IFN-γ staining. Scale bars, 100 μm

Elevated levels of IFN-γ and STAT1 signaling are implicated in chronic lymphoid-driven human diseases, such as SLE^36, 39, 42^ and in mouse models of SLE, including the MRL/*lpr* strain^39, 40^. The signature lymphoproliferative response in female MRL/*lpr* mice, as evidenced by enlarged lymph nodes, was seen by day 90 in female *Mmp12*^*–/–*^ (*n* = 18) and *Mmp12*^*+/+*^ (*n* = 38) mice (Fig. 6a, Supplementary Fig. 9a, b, *N* = 2) and later for males (*n* = 13 each for *Mmp12*^*–/–*^ and *Mmp12*^*+/+*^ male mice, Supplementary Fig. 9a). Consistent with the MMP12-dependent differences in lymph node size apparent by day 90 in the female mice, CFA synchronization further expanded the differential increase in lymphadenopathy manifest in the *Mmp12*^*–/–*^ MRL/*lpr* females (*n* = 18, *N* = 2) over that seen for the female *Mmp12*^*+/+*^ MRL/*lpr* mice (*n* = 38, *N* = 2) (Fig. 6a, Supplementary Fig. 9b), supporting a role for MMP12 in dampening acute inflammatory responses.

As in human autoimmune disease, where there is significantly increased prevalence in females versus males^43^, female mice in the absence of CFA had a shortened survival time than males (112 days versus 131 days) before lymphadenopathy led to humane endpoint euthanasia (Fig. 6b, Supplementary Fig. 9c-e, *N* = 2). Protein levels of IFN-γ and the IFN-γ-activated macrophage markers iNOS and MHCII were significantly increased in the superficial cervical lymph nodes of the female *Mmp12*^*–/–*^ MRL/*lpr* mice compared with female *Mmp12*^*+/+*^ MRL/*lpr* mice (day 98) (Fig. 6c, d). As immunohistochemistry is only semi-quantitative we confirmed the elevation of these IFN-γ classical activation macrophage proteins, plus STAT1, S100A8, and S100A9, in lymph node extracts of the knockout versus the wild-type *Mmp12* MRL/*lpr* mice (Fig. 6e). Of note, we detected lower molecular weight forms of IFN-γ in *Mmp12*^*+/+*^ but not *Mmp12*^*-/-*^ mice (Fig. 6e), suggestive of in vivo processing of IFN-γ. Thus, the lack of MMP12 was associated with a shift in macrophage populations to one retaining ongoing IFN-γ-activation in vivo.

In association with the enlarged cervical lymph nodes in *Mmp12*^*–/–*^ MRL/*lpr* female mice (Fig. 6a), we found a relative decrease in CD68^+^ cells consistent with previous reports^22, 23^ and elevated inflammatory markers e.g., increased neutrophil myeloperoxidase, TUNEL and CD31^+^ cells (Supplementary Fig. 10b). Although, spleen weights in both sexes were unchanged with or without MMP12 (Supplementary Fig. 9f) and there were no significant differences in the number of circulating leukocytes and monocytes in *Mmp12*^*+/+*^ (*n* = 15) and *Mmp12*^*–/–*^ (*n* = 27) MRL/*lpr* female mice (Supplementary Fig. 10a), we observed increased kidney weights in *Mmp12*^*–/–*^ MRL/*lpr* female mice (*n* = 15) versus *Mmp12*^*+/+*^ MRL/*lpr* littermates (*n* = 11) (Fig. 6f). Histological scores of Activity Index and Chronicity Index^44, 45^ were significantly elevated in *Mmp12*^*–/–*^ MRL/*lpr* female mice (*n* = 15) versus *Mmp12*^*+/+*^ MRL/*lpr* littermates (*n* = 11) (Fig. 6g), indicating both worse acute lupus nephritis activity and resultant chronicity. The concentration of MHCII and IFN-γ proteins were significantly elevated in *Mmp12*^*–/–*^ kidney glomeruli compared with *Mmp12*^*+/+*^ littermates, reflecting a shift in the *Mmp12*^*–/–*^ MRL/*lpr* macrophage population in vivo (Fig. 6h–j). Overall, in the MRL/*lpr* model of SLE, *Mmp12*^*–/–*^ mice exhibited sustained IFN-γ signaling favoring ongoing proinflammatory responses versus *Mmp12*^*+/+*^ MRL/*lpr* mice, which exhibited normal macrophage phenotypic succession to alternatively activated CD36+ macrophages with time.

### C-terminal cleavage of IFN-γ is reduced in lupus nephritis

**Fig. 7:**
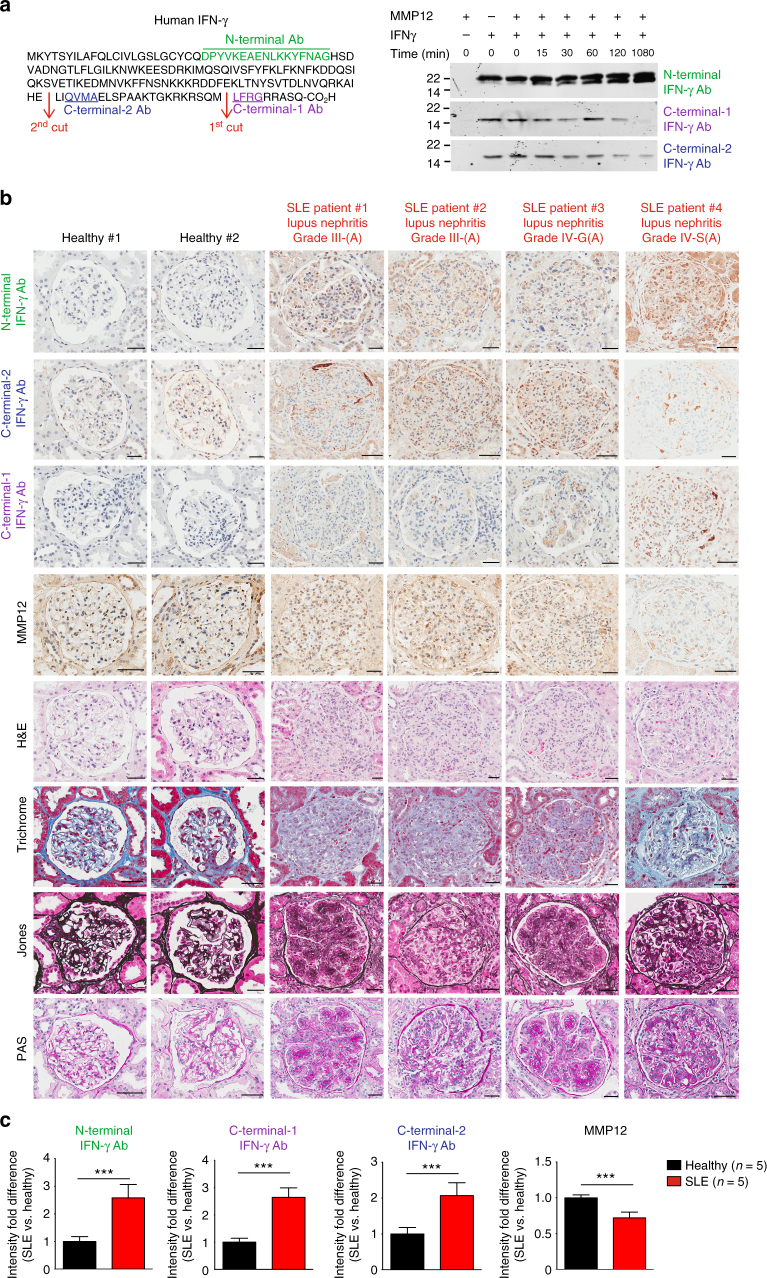
IFN-γ epitope antibody staining of human lupus nephritis biopsies. **a** Left, amino acid sequences (underlined) of human IFN-γ peptides used to raise and affinity-purify rabbit anti–N-terminal, C-terminal-1, and C-terminal-2 IFN-γ epitope antibodies. Right, western blot analysis of human IFN-γ after time-dependent cleavage to 1080 min by human MMP12. Note: disappearance of the C-terminal epitopes as MMP12 cleavage proceeds. Molecular weight marker positions in all blots are as shown. **b** Immunostaining of human kidney biopsies using anti–N-terminal, C-terminal-2, C-terminal-1, and MMP12 antibodies; and staining with hematoxylin and eosin (H&E), Trichrome, Jones, and PAS of kidney biopsies of healthy (*n* = 5), lupus nephritis at Grades III-(A) (*n* = 3) and IV-(A) (*n* = 2) as diagnosed in Supplementary Fig. 11. Scale bar, 100 μm. Original magnification, ×400. **c** Quantification of immunostaining intensities in healthy (*n* = 5) and lupus nephritis (*n* = 5) kidney biopsies (additional data are included in Supplementary Fig. 11) and expressed as means ± s.d. Statistical significance was determined by a two-tailed unpaired Student’s *t*-test: ***p < 0.005

We next analyzed MMP12 and IFN-γ protein levels and the IFN-γ cleavage status in patients with lupus nephritis (Supplementary Table 1). To do so we raised and affinity-purified three antibodies to human IFN-γ to determine its activation status in vivo (Fig. 7a). To profile total (active plus inactive) IFN-γ protein, we raised an antibody against the N-terminal IFN-γ peptide, 25DPYVKEAENLKKYFNAG41. To identify full-length IFN-γ protein, we raised antibodies against the C-terminal sequence 158LFRG161 (C-terminal-1 antibody) distal to the first cleavage site and therefore only present in non-cleaved IFN-γ. By recognizing a sequence (QVMA) between the 1st and 2nd MMP12 cleavage sites, the C-terminal-2 antibody discriminated between the two C-terminal MMP12-truncated forms. So, whereas positive staining for the N-terminal antibody identified all 3 forms of IFN-γ, including the inactive MMP12-truncated IFN-γ 23–135, the C-terminal-2 antibody confirmed the presence of both the active IFN-γ proteoforms past position 135. As the human and murine IFN-γ sequences diverge significantly in the C-terminus (Supplementary Fig. 4b), the anti-human IFN-γ antibodies did not cross react with mouse IFN-γ.

After a time-course incubation of IFN-γ with MMP12 for up to 1080 min the presence of a C-terminal cleaved form of IFN-γ was apparent using the N-terminal antibody, even as soon as 15 min (Fig. 7a), confirming the kinetic data of efficient cleavage. The C-terminal-2 antibody will recognize both full-length and IFN-γ cleaved at 157Met↓Leu158. Decreasing immunoreactivity for both C-terminal epitopes over time in vitro confirmed cleavage removal of the short C-terminal peptides (Fig. 7a). Although cleavage at 135Glu↓Leu136 can occur first, the loss-of-C-terminal-1 immunoreactivity with a weak C-terminal-2 band still present at 1080 min indicated that 157Met↓Leu158 cleavage can also precede 135Glu↓Leu136 cleavage. Therefore, the prominent lower immunoreactive band detected by the strong N-terminal antibody is likely the 135Glu↓Leu136 cleaved inactive IFN-γ proteoform.

With these antibodies, we analyzed IFN-γ cleavage status in five healthy subjects and five active (A) patients with SLE Stage III-(A) (*n* = 3) or Stage IV-(A) (*n* = 2) lupus nephritis (Fig. 7b, Supplementary Fig. 12 and Supplementary Table 1). As compared to the normal biopsies, the SLE biopsies displayed a significant fold-increase in total glomerular IFN-γ, as shown by the anti–N-terminal antibody (Fig. 7c). This included significant increases in full-length IFN-γ (23–166), as revealed by immunoreactivity against the N-terminal and C-terminal-1 antibodies, and significant increases in active IFN-γ in the SLE patients, as revealed by positive immunostaining of both anti–C-terminal antibodies. Indeed, the significant immunostaining by the anti–C-terminal antibodies was associated with lower MMP12 immunostaining, supporting the hypothesis of reduced MMP12–mediated IFN-γ cleavage in SLE vs. normal kidney.

### MMP12 modulation of STAT1 signaling in primary macrophages

**Fig. 8:**
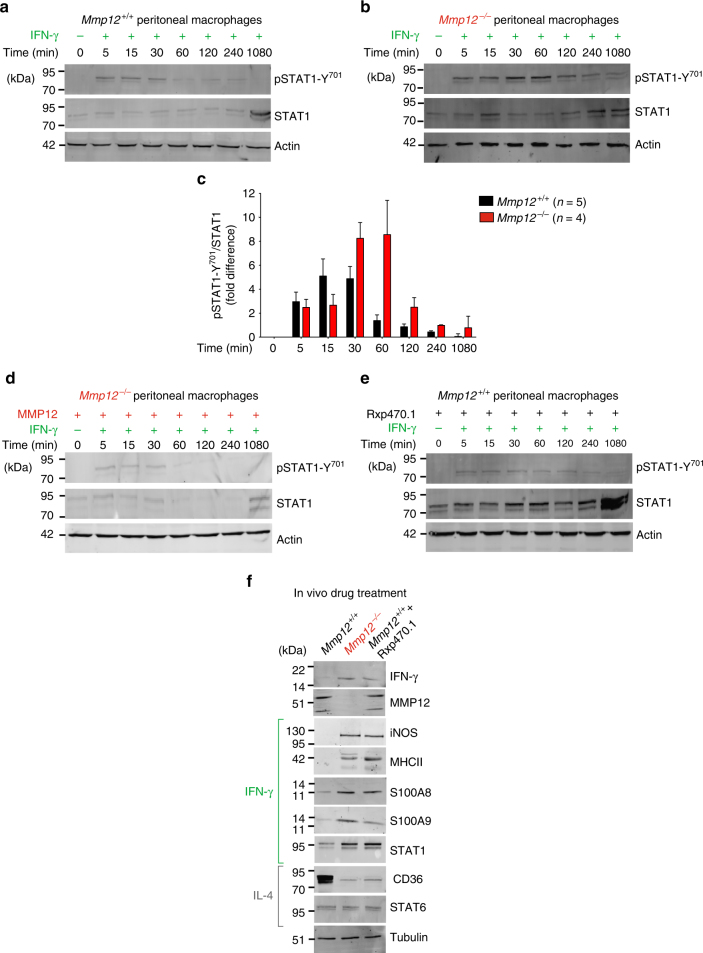
Prolonged IFN-γ signaling in *Mmp12*^*–/–*^ primary peritoneal macrophages. Western blot analysis for pSTAT1-Y701 and STAT1 in primary peritoneal macrophages harvested from individual (**a**, **e**) *Mmp12*^*+/+*^ B10.RIII (*n* = 5 for each time point) and (**b**, **d**) *Mmp12*^*–/–*^ B10.RIII (*n* = 4 for each time point) mice 4 days after induction of peritonitis. Cells were treated with 20 ng/mL of IFN-γ for 0–1080 min. **c** Ratios of pSTAT1-Y701 to STAT1 protein levels were determined after densitometry quantification of the western blots. The data are expressed as fold differences in the ratio of the means for *Mmp12*^*+/+*^ (*n* = 5 for each time point) and *Mmp12*^*–/–*^ (*n* = 4 for each time point) B10.RIII mice. **d** Rescue of *Mmp12*^*–/–*^ peritoneal macrophages with recombinant mouse MMP12 protein (1:100) incubated for the times shown (*n* = 4 for each time point). **e** *Mmp12*^*+/+*^ B10.RIII macrophages were incubated for 30 min with 100 nm specific MMP12 inhibitor Rxp470.1 before addition of 20 ng/mL IFN-γ (*n* = 4 for each time point). **f** Western blot analysis of IFN-γ, MMP12, iNOS, MHCII, S100A8, S100A9, STAT1, CD36, and STAT6 proteins in primary peritoneal macrophages harvested from *Mmp12*^*+/+*^ (*n* = 4) and *Mmp12*^*–/–*^ (*n* = 4) B10.RIII mice at day 4 post-intraperitoneal injection with vehicle; and *Mmp12*^*+/+*^ B10.RIII mice treated daily with 5 mg/kg Rxp470.1 (*n* = 4) for 4 days during the induction of peritonitis. Actin or tubulin loading controls and molecular weight marker positions in all blots are as shown

To mechanistically explore the in vivo consequences of loss-of-MMP12 on pSTAT1-Y701 signaling by IFN-γ, we induced peritonitis in *Mmp12*^*+/+*^ B10.RIII mice (*n* = 5) and *Mmp12*^*–/–*^ B10.RIII mice (*n* = 4). At day 4, we collected peritoneal macrophages and stimulated the primary macrophages with IFN-γ. In the *Mmp12*^*+/+*^ cells, we observed the characteristic pulse in pSTAT1-Y701 levels that decayed after ~30 min (Fig. 8a). In contrast, upon IFN-γ treatment primary macrophages from the *Mmp12*^*–/–*^ mice displayed prolonged activation of pSTAT1-Y701 phosphorylation to 60 min and beyond (Fig. 8b, c).

We then performed gain- and loss-of-function experiments. Treatment with recombinant mouse MMP12 of four individual cultures of *Mmp12*^*–/–*^ B10.RIII primary macrophages harvested from four mice (Fig. 8d) terminated the sustained activation of pSTAT1-Y701 seen in the vehicle control *Mmp12*^*–/–*^ cells without exogenous MMP12 (Fig. 8b). Hence adding MMP12 rescued the temporal pattern of pSTAT1-Y701 phosphorylation to that resembling *Mmp12*^*+/+*^ B10.RIII (*n* = 5) primary peritoneal macrophages, which also decayed at ~30 min (Fig. 8a). Similarly, treatment of primary *Mmp12*^*+/+*^ B10.RIII macrophage cultures with the selective MMP12 inhibitor, Rxp470.1 (Fig. 8e), extended the activation of pSTAT1-Y701 and recapitulated the *Mmp12*^*–/–*^ B10.RIII primary cell responses (Fig. 8b). Hence, a genetic lack of MMP12 or the specific inhibition of MMP12 prolonged STAT1 signaling, thereby providing an explanation for the IFN-γ-classically activated macrophage dominated profile of *Mmp12*^*–/–*^ B10.RIII mice in vivo.

Finally, to directly link MMP12 catalytic activity with the controlled normal succession of IFN-γ-classically activated macrophage populations to alternately activated macrophages in vivo, we injected *Mmp12*^*+/+*^ B10.RIII mice with 5 mg/kg MMP12-specific inhibitor (Rxp470.1) (*n* = 4) or vehicle (*n* = 4) daily for 4 days during the development of peritonitis. Although MMP12 is predominantly a macrophage-associated protease^21, 22^, other cells also express the enzyme^6^, which systemic administration of Rxp470.1 will block. In primary macrophages of the vehicle controls harvested at day 4 we found that the cell population was enriched for macrophages with high CD36 expression (indicative of the alternative macrophage activation pathway), an absence of iNOS and MHCII proteins, and low levels of other IFN-γ-classically activated macrophage signature proteins (Fig. 8f). This represents the normal temporal evolution of macrophage phenotypes in acute inflammation. In contrast, treatment of the *Mmp12*^*+/+*^ B10.RIII mice (*n* = 4) for 4 days with Rxp470.1 shifted the day 4 peritoneal macrophage population to one retaining the early proinflammatory IFN-γ-classically activated macrophage characteristics of *Mmp12*^*+/+*^ B10.RIII at day 2 (Fig. 3b, d) or *Mmp12*^*–/–*^ B10.RIII mice at day 4 (Figs. 8f and 3b, d). Thus, IFN-γ, iNOS, MHCII, S100A8, S100A9, and STAT1 proteins were elevated in peritoneal macrophages harvested from the Rxp470.1-treated *Mmp12*^*+/+*^ mice compared with vehicle-treated *Mmp12*^*+/+*^ mice at day 4 and the profile now matched that of the *Mmp12*^*–/–*^ mice at day 4. Notably, this loss-of-controlled evolution in macrophage populations over time was also a feature of *Mmp12*^*–/–*^ B10.RIII arthritis at 28 days (Fig. 5) and lymphadenopathy in MRL/*lpr* mice at 96 days (Fig. 6e). Thus, injection of a specific MMP12 inhibitor in vivo in this loss-of- function experiment directly linked MMP12 catalytic activity with the normal controlled shift in proinflammatory to immunosuppressive macrophage cell phenotype ratios occurring in vivo.

## Discussion

It is important to recognize all levels of control of key cytokines contributing to the transition from proinflammatory to immunosuppressive macrophage dominated inflammation, and therefore disease risk, susceptibility and resolution. IFN-γ is one of the main effectors in autoimmune diseases, such as SLE, rheumatoid arthritis, and multiple sclerosis^19, 46^. However, the role of post-translational modification of IFN-γ in these diseases has not been previously recognized. We demonstrated that MMP12, primarily a monocyte-macrophage protease^21, 22^, downregulates IFN-γ activity by precise C-terminal cleavage at an evolutionary conserved site that prevents phosphorylation of STAT1-Tyr701, and thus IFN-γ signaling and classical activation of macrophages. Initial MMP12 cleavage then sets the stage for IFN-γ clearance in vivo that reduces total IFN-γ levels over time. By forming a post-translational negative feedback mechanism, we found that the normal succession from IFN-γ-classically activated proinflammatory to alternately activated macrophages was dampened when MMP12 was genetically deficient or therapeutically inhibited, especially in acute inflammation, but also in chronic inflammatory autoimmune models experiencing disease flares. Thus, there was a clear shift in inflammatory cell infiltrates to populations enriched for IFN-γ-activated macrophages with attendant tissue pathology in *Mmp12* knockout mice in three different models of inflammation, in both male and female mice, and on two different genetic backgrounds.

We observed an important role for the MMP12/IFN-γ processing axis in acute inflammation in models of peritonitis and in the acute collagen-induced model of arthritis. The role of MMP12 was also apparent in MRL/*lpr* mouse models of arthritis and lupus, especially after CFA hyperinduction of IFN-γ, where differences in its activity would be more manifest depending upon MMP12 initiated inactivation and clearance. This suggests an important role of MMP12 in dampening disease flares. We further showed the significance of dysregulated IFN-γ in human disease by demonstrating that reduced MMP12 mRNA levels were correlated with systemic lupus erythematosus clinical deterioration over time, and also demonstrated increased active IFN-γ in glomeruli of patients suffering from lupus nephritis when compared to healthy individuals, highlighting a potential diagnostic and therapeutic path. Adding to mechanisms in regulation of macrophage activation and to other anti-inflammatory activities of MMP12^23^, including C-terminal cleavage and inactivation of IFN-α^6^ and antibacterial roles^26^, IFN-γ C-terminal truncation provides a new level of post-translational negative feedback control in the evolution of macrophage populations. Our present work further challenges the classical detrimental roles ascribed to other MMPs that are elevated in SLE^47, 48^.

Numerous pathways and cell types are important in the pathogenesis of chronic inflammation and autoimmune diseases. Multifactorial control of normal macrophage polarization leads to many possible break points that can fail, increasing the risk of chronic inflammation and autoimmunity. Such complexity renders diagnosis and treatment challenging. We were able to translate our findings identifying proteolytic control of IFN-γ activity and IFN-γ activation of macrophages to human autoimmune disease. By analyzing previous data, we found that SLE diagnosed by multiple clinical parameters, including elevated anti-double stranded DNA antibody levels, was associated with lower PBMC *MMP12* mRNA levels than in healthy subjects. Further, with clinical improvement upon treatment during the chronic inflammatory phase of the disease, *MMP12* mRNA expression increased to near healthy control levels. In a different study in which clinical responses after treatment of 40 SLE patients were examined, we found a strong association in the reduction of IFN-γ response gene expression that coincided with elevated expression of *MMP12* mRNA and IL-4 alternatively activated macrophage markers. We found that *MMP12* normalization occurs after instituting treatment and precedes clinical improvement, whereas patients whose *MMP12* levels are not affected appeared not to improve. Although this clinical association does not prove a mechanistic link for MMP12 in protection from autoimmunity in human disease, it supports the findings from our animal model studies.

The temporal integration of opposing cytokine signals important in differentiation fate decisions for IFN-γ classical activation versus IL-4 activation and other alternate macrophage activation pathways, and the spectrum of cell populations occurring in between, is a multi-faceted, highly nuanced immune mechanism that underpins the development of chronic inflammation and autoimmunity and leads to acute clinical relapses. As *MMP12* expression is higher in IL-4 activated macrophages than in IFN-γ-activated cells, *MMP12* expression by alternatively activated macrophages may amplify *in trans* the reduction in IFN-γ activity by IFN-γ-activated macrophage MMP12 to reinforce the decrease in proinflammatory cells. Conversely, low-MMP12 activity favors chronic disease progression and so may be a risk factor for SLE, and potentially other autoimmune diseases. Our findings also further emphasize that using genomic or transcriptomic data alone risks overlooking the key importance of post-translational modifications of immune regulatory and autoimmune signature cytokines. The present data establishing the proteolytic regulation of IFN-γ activity in acute inflammation, including disease flares of chronic disease, suggest a more generally applicable mechanism not limited to SLE or lupus nephritis, but also potentially at play in other IFN-γ-linked phenotypes. Having identified that MMP12 expression provides a benefit in mouse models of IFN-γ-driven inflammatory diseases, it will be important to prospectively evaluate MMP12 activity in a larger cohort of patients with diseases such as SLE. If activity is significantly lower in a large patient cohort with active disease, it will provide crucial information that potentially may point to new therapeutic modalities that restore normal cytokine cellular control to augment the production of MMP12.

## Methods

### MMP12 cleavage assays of IFN-γ

Recombinant human and mouse MMP12, expressed and purified as described^49^, were incubated with 10 or 100 ng human or mouse MMP12, 37 °C in assay buffer (100 mM Tris-HCl, 50 mM NaCl, 5 mM CaCl_2_, pH 8.0) at enzyme/substrate ratios from 1:10 to 1:100. IFN-γ cleavage was determined by silver staining of 15% Tris-Tricine gels and western blots. To identify cleavage sites, cleaved peptides were analyzed by the Edman sequencing, liquid chromatography tandem–mass spectrometry (LC–MS/MS), and MALDI-TOF MS on a Applied Biosystems Voyager DE STR Mass Spectrometer as described^50^. The specific MMP12 inhibitor RXP470.1^6, 30^ was a control in the cleavage assays. The *k*_cat_/*K*_M_ values were quantified according to (1) from IFN-γ fragment generation over time as described^50^:

1 $$\frac{{k}_{\rm{cat}}}{{K}_{\rm{M}}} = \frac{k}{{[E]}}\,{\rm where}\,k = \frac{{{\rm ln}2}}{{t_{1/2}}} = \frac{{0.69}}{{t_{1/2}}}.$$

### Top down mass spectrometry

IFN-γ and MMP12 cleavage fragments were analyzed by top down liquid chromatography tandem–mass spectrometry (LC–MS/MS). After cleavage (15 min or 240 min), samples were first purified by solid phase extraction on Empore Octyl C8 disks (3M Empore) packed as a C18 StageTips. Tips were activated with 50% acetonitrile, equilibrated with 0.1% formic acid, samples were acidified, loaded and then washed with 2 column volumes of 0.1% formic acid, and eluted in 50% acetonitrile, 0.1% formic acid. Eluates were directly infused into an Impact II high resolution, high-mass accuracy quadrupole time-of-flight (QTOF) system using a CaptiveSpray ion source (Bruker Daltonics) at a flow rate of 3 µL/min, ionized with 4.5 kV, 0.3 bar nebulizer gas pressure, heated to 180 °C and analyzed in positive ion mode. MS1 spectra were acquired in the mass range of 110–5000 m/z at a scan rate of 1 Hz. Following acquisition, a composite MS1 spectrum was generated by summing all MS1 spectra during acquisition time using Compass DataAnalysis v4.3 (Bruker Daltonics). Spectra were deconvoluted by mMass and used to determine the mass of peptide and protein ions. Default mMass baseline correction and smoothing parameters were used.

### In silico structural analysis

Based on the crystal structures of the IFN-γ dimer (pdb entry 1HIG^51^ [10.2210/pdb1HIG/pdb]) and IFN-γ receptor complexes (1FG9 [10.2210/pdb1FG9/pdb]^32^ and 1FYH [10.2210/pdb1FYH/pdb]^33^) we modeled a secondary complex consisting of (i) the IFNG dimer (UniProt P01579; Gln24-Leu158), (ii) two high-affinity IFNG receptor 1 molecules (IFNGR1; P15260; Val29-Ser241), and (iii) two low affinity IFNG receptor 2 chains (IFNGR2; P38484; Leu30-Thr237). Missing loops were resolved using ModLoop^52^ and truncated side chains were rebuilt using Dunbrack’s backbone-dependent rotamer library^53^ within Chimera (15264254). The C-terminal IFNG sequence (Pro145-Leu158) was based on published biochemical information^34, 54^ and our data in this manuscript using the Rosetta FlexPepDock web server^55^ and Modeller^56^ within Chimera^57^, and manually evaluated. Molecular graphics figures were made using the molecular visualization system PyMOL (The PyMOL Molecular Graphics System, Version 1.7.1.3, Schrödinger, LLC)^58^.

### Mice

*MMP12*–deficient mice on the C57BL/6 × 129Sv/Ev background were generated as described by Shipley et al.^22^ with *Mmp12* exon 2-disrupted embryonic stem cells injected into C57BL/6J blastocysts. After the chimeras were crossed to C57BL/6J mice they were backcrossed for 10 generations. As the C57BL/6J strain is considered a low inflammation strain in these disease models, we then backcrossed these homozygous mice for 8 generations onto the MRL/MpJ-*Fas*^*lpr*^/J (MRL/*lpr*) strain (catalog no. substrain 000485; The Jackson Laboratory). Thereafter, the *Mmp12*^*+/+*^ MRL/*lpr* and *Mmp12*^*–/–*^ MRL*lpr* male and female mice, housed in ventilated racks, were maintained as homozygous lines, leading to an autoimmune disease mouse model after ~90 days that can be synchronized between mice upon injection of CFA for severe arthritic and kidney disease development^41^ before gross lymphadenopathy reached humane end points as determined by the UBC Animal Care Committee.

To analyze collagen-induced arthritis, we backcrossed *Mmp12*^*–/–*^ mice on the 129Sv/Ev background for seven generations onto the B10.RIII-H2^r^ *H2-T18*^*b*^ background (The Jackson Laboratory, stock #000457^23^). Genotyping was performed by the HotShot DNA isolation followed by polymerase chain reaction with *Mmp12* primers (forward) and (reverse) and a primer specific for the neomycin insert (reverse). The PCR products were 1100 bp for *Mmp12*^*+/+*^ mice and 550 bp for *Mmp12*^*–/–*^ mice. The UBC Animal Care Committee approved all mouse breeding and animal experimental procedures and, established and supervised humane end points.

### Thioglycollate-induced sterile peritonitis

At day 90, male and female mice were injected intraperitoneal with 0.5 mL 4% Brewers thioglycollate broth (Sigma-Aldrich, St. Louis, MO) to establish acute sterile peritonitis. Mice were excluded from further studies if obvious adhesions were observed between the peritoneum and skin or if marked increases were observed in the thickness of the peritoneum. Mice were killed at day 0, 1, 2, 3, or 4 and 5 mL of phosphate buffered saline (PBS) was administered via intraperitoneal injection to collect infiltrating cells and peritoneal exudate. Cells were plated for 4–18 h in RPMI 1640 medium, 10% cosmic calf serum and non-essential amino acids. For in vivo MMP12 inhibition, we administered 5 mg/kg Rxp470.1 intraperitoneal once per day for 4 days in *Mmp12*^*+/+*^ B10.RIII (*n* = 4) and *Mmp12*^*–/–*^ B10.RIII (*n* = 4) mice and collected the peritonitis fluid and cells by PBS lavage on day 4.

### Cells

Human THP-1 monocytic cells (ATCC, Manassas, Virginia, USA), mouse RAW264.7 macrophages (ATCC, Manassas, Virginia, USA) and primary mouse peritoneal macrophages were cultured in 10% fetal bovine serum (v/v) in RPMI. All cell lines were tested and found to be mycoplasma negative.

### Macrophage activation

Human THP-1 cells (5 × 10^6^ cells in 10 mL) were treated with phorbol 12-myristate 13-acetate (PMA) (100 ng/mL) for 24 h to induce cell attachment and differentiation. THP-1 cells and mouse RAW264.7 cells were activated with 20 ng/mL of IFN-γ (human #300-02, PeproTech, or mouse #315-05, Rocky Hill, NJ, respectively), or 30 ng/mL IL-4 (human #200-04 PeproTech, or mouse #214-14, Rocky Hill, NJ, respectively).

### Quantification of intracellular radical oxygen species

Cellular ROS levels were determined by incubating 1 × 10^5^ cells with 5 μg/mL 2,-7-dichlorofluorescein diacetate (DCF-DA, Sigma-Aldrich) for 15 min at 37 °C (*n* = 4, *N* = 2). Cells were washed twice with Hank’s complete balanced saline solution (HBSS) and harvested after trypsinization, and the fluorescence (excitation 488 nm, emission 525 nm) was measured using a POLARstar optima (BMG Labtech, Durham, NC). Statistical analysis was determined by a two-tailed unpaired Student’s *t*-test.

### Phagocytosis assay

THP-1 cells (5 × 10^6^ cells in 10 mL) were stimulated with 200 ng/mL PMA for 24 h to induce cell attachment and differentiation and then the medium was changed. A 2.69% Fluoresbrite® 2-μm microparticle suspension (12 μL) (Polysciences, Inc., Warrington, PA) was incubated in 175 μL normal human serum (Complement Technology Inc., Tyler, TX) for 30 min at 37 °C before addition to the PMA-activated cells for 1 h. Phagocytosis was stopped by adding 2 mL of ice-cold HBSS. The bead solution was removed, and the cells were washed twice with HBSS, then harvested by trypsinization and washed. The phagocytosis index was determined in each experiment (*n* = 3 repeats per experiment, and the experiment itself performed twice, *N* = 2) by counting the number of beads per cell in 20 separate fields containing 5–30 cells/image for each repeat. Statistical analysis was determined by a two-tailed unpaired Student’s *t*-test.

### Antibodies

Anti-Mouse CD11c FITC (clone N418) was from BioLegend and used at a concentration of 1 μg/mL × 10^6^ cells. Polyclonal rabbit anti-STAT1 (#9172), pSTAT1-Y701 (#9167), STAT6 (#9362), and pSTAT6-Y641 (#9361) were used at a dilution of 1/1000 and were from Cell Signaling. Polyclonal rabbit anti-iNOS (ab15323), anti-IFN-γ (EPR1108, ab133566), anti-MHC Class II (ab55152), and anti-CD36 (ab137320) were used at a dilution of 1/1000 and were from Abcam. Polyclonal goat anti-S100A8 (AF3059) and anti-S100A9 (AF2065) were used at a dilution of 1/1000 and were from R&D Systems. Polyclonal rabbit anti-MMP12 catalytic domain antibody and anti-hinge domain antibody (Triple Point Biologics) were used at a dilution of 1/1000. Anti-mouse myeloperoxidase (MPO) from Hycult (HM1051) were used at a dilution of 1/100 on tissue sections.

### Raising and affinity purification of IFN-γ epitope antibodies

Three IFN-γ peptides (GenScript, Piscataway, NJ): DPYVKEAENLKKYFNAG-GC; C-GGLFRG, and CRGG-QVMA (Arg was added for solubility), all synthesized with either a G or GG added as a flexible spacer to a cysteine to enable conjugation to succinimidyl 4-(N-maleimidomethyl) cyclohexane-1-carboxylate/Keyhole Limpet Hemocyanin (Sigma-Aldrich, St-Louis, MO). The conjugates were emulsified in CFA and injected in New Zealand white rabbits. Antibody expression was boosted with peptide in incomplete Freund’s adjuvant at 3 weeks. Serum antibody titers to the respective peptides were quantified by ELISA. SulfoLink Immobilization (Pierce Biotechnology, Rockford, IL) was used to conjugate the peptides to affinity-purify antibodies from rabbit sera. Peptides (5 mg) were dissolved in 2 mL of coupling buffer (50 mM Tris, 5 mM EDTA, pH 8.5), reduced with 25 mM TCEP, and incubated for 30 min. The sulfhydryl-containing peptides were coupled to 3 mL SulfoLink columns by mixing with the SulfoLink resin for 15 min and incubated for 2 h. Non-specific binding sites were blocked with 50 mM cysteine for 1 h. The columns were then equilibrated with 50 mM Tris, 5 mM EDTA, pH 8.5 before affinity purification of anti-peptide antibodies from rabbit sera. After column washes, high-affinity antibodies were eluted using 0.1 M glycine, pH 2.5. Antibody specificity was verified by ELISA and western blotting to IFN-γ and MMP12-cleaved IFN-γ. Western blotting of human kidney extracts was used to assess specificity and non-specific binding before translation to ELISA assays.

### ELISA

Plates were coated with 5 μg/mL of each of the 3 IFN-γ peptides in Vollers buffer (15 mM Na_2_CO_3_, 35 mM NaHCO_3_, pH 9.6) overnight and then blocked with 1% BSA in PBS for 1 h. The wells were rinsed three times with PBS-0.05% Tween 20 and 100 μL of a serial dilution of antibodies (1:10^2^–1:10^9^) were added and incubated at room temperature for 2 h. The wells were rinsed with PBS-Tween 20 (0.05%) before 100 μL of 1:1000 goat anti-rabbit HRP antibody (Bio-Rad Laboratories, Hercules, CA) was added and incubated for 1 h. Wells were aspirated and incubated in the dark with SIGMAFAST™ OPD HRP substrate (Sigma-Aldrich, St-Louis, MO) until visible coloration was seen. At this time 50 μL of 1 M acetic acid was added to stop the reaction and the absorbance was measured at 450 nm on a POLARstar optima microplate reader (BMG Labtech, Durham, NC).

### Collagen-induced arthritis in B10.RIII mice

Collagen-induced arthritis was induced by injection with type II collagen (4 mg/mL) at two inguinal sites in 49-day-old-male *Mmp12*^*–/–*^ (*n* = 20) and *Mmp12*^*+/+*^ mice (*n* = 18) all on the B10.RIII background. Day 0 was defined as 21 days after injection and was typically the date of onset of arthritis. At 18 and 28 days later, the mice were killed and the hind ankles were dissected and prepared for immunostaining and histological staining by hematoxylin and eosin (H&E) and toluidine blue to visualize cartilage proteoglycan^23^. NETs were previously characterized in collagen-induced arthritis by a variety of stains, including hematoxylin and eosin in combination^23^ or alone^59, 60^, the histological appearance and distribution of which are consistent with the NETs identified here by hematoxylin and eosin.

### Arthritis in MRL/*lpr* mice

Spontaneous arthritis and lupus nephritis are a characteristic of the MRL/*lpr* strain. To ensure reasonably severe and consistent disease stage between animals before excessive lymphadenopathy necessitated animal euthanasia it was necessary to synchronize the initiation of systemic autoimmunity by CFA earlier than spontaneous disease was manifest. We injected 0.05 mL of a 1:1 emulsion of CFA (Sigma) in water intradermal at two inguinal sites in 90-day-old mice^41^. The width of both hind ankles was measured on three consecutive days before injection and every 2–3 days for 25 days following injection using electronic digital calipers (Marathon). Baseline ankle width was calculated as the average of the pre-injection measurements. Arthritis was deemed present with a 10% increase in average ankle width. Spleen weights were determined of female *Mmp12*^*–/–*^ MRL*/lpr* (*n* = 22) and *Mmp12*^*+/+*^ MRL*/lpr* (*n* = 27) mice with or without CFA injection. Statistical analysis was performed with Prism 5 (GraphPad Software) using two-way analysis of variance with Bonferroni post-tests.

### Immunostaining of biopsy sections

Mouse ankle, lymph node and kidney, and human kidney biopsies were dissected and formalin-fixed for 16 h. Hind ankles were decalcified in 10% formic acid for 72 h. Biopsies were then dehydrated through graded ethanol series and paraffin-embedded. Tissue sections (3–5 μm) were deparaffinized, rehydrated through an ethanol series ending in water and stained with hematoxylin and eosin, toluidine blue, trichrome, Jones, or Periodic acid-Schiff (PAS). For immunostaining, tissue sections were rehydrated, quenched to remove endogenous peroxidase, blocked in 1/10 normal goat serum for 1 h and then incubated with antibody in Tris-buffered saline. Primary antibodies and species appropriate secondary antibodies were used as performed by Wax-it Histology Services (Vancouver, BC, Canada).

### Mouse glomerulonephritis activity and chronicity index scoring

The means of the activity and chronicity scores from *Mmp12*^+/+^ MRL/*lpr* (*n* = 11) and *Mmp12*^–/–^ MRL/*lpr* (*n* =15) mice were calculated, for each biopsy, by analysis of 10 components divided into two scoring systems: Renal activity index (1—cellular proliferation, 2—leukocyte infiltration, 3—fibrinoid necrosis or karyorrhexis, 4—cellular crescents, 5—hyaline thrombi, wire loops and 6—mononuclear cell infiltration), and Renal chronicity index (7—glomerular sclerosis, 8—fibrous crescents, 9—interstitial fibrosis, 10—tubular atrophy)^61, 62^. Each individual component was scored 0 (for normal), 1, 2, or 3 (for severe abnormality, 3 being the worse). In calculating the activity index, fibrinoid necrosis and cellular crescents were weighed by a factor of 2. The renal activity index scale ranges from 0 to 24 points and the renal chronicity index scale from 0 to 12.

For immunostaining analyses of mouse kidney glomeruli, and glomeruli plus the interstitium a minimum of 10 different fields, with each field having a minimum of 5 glomeruli were examined and quantified for each antibody, for each of these two separate analyses (*N* = 2). Three mice per group (*Mmp12*^+/+^ (*n* = 3) and *Mmp12*^–/–^ MRL/lpr (*n* = 3)) were analyzed separately for the glomeruli and the glomeruli and interstitium staining quantifications.

### Mouse whole-blood leukocyte and monocyte counts

Whole blood was collected from mice in EDTA-collection tubes and analyzed on a Siemens Advia 120 Hematology System (Oakville, ON, Canada) with the Perox method. The whole blood of each mouse (*Mmp12*^+/+^ (*n* = 15) and *Mmp12*^*–/–*^ (*n* = 27) MRL/*lpr*) was analyzed in technical duplicates.

### *MMP12* mRNA analyses

Relative expression levels of *MMP12* mRNA was measured in PMA-matured THP-1 cells treated with 20 ng/mL IFN-γ, 30 ng/mL IL-4, or PBS for 24 h (*n* = 3, *N* = 2 for each condition). After isolating RNA using the RNeasy Mini system (Qiagen), total RNA (1 μg) was amplified (Ambion MessageAmp II amplification kit, Thermo Scientific) and labeled by ULS aRNA fluorescent labeling (Kreatech Biotechnology, Amsterdam, The Netherlands). ULS-Cy3 labeled RNA was mixed 1:1 with ULS-Cy5 labeled control RNA (equimix of all amplified RNA samples) and hybridized to the CLIP-CHIP^TM^ human protease and inhibitor targeted microarray^63^ for 18 h, 42 °C in duplicate with three biological replicates (i.e., six data points/gene). The microarray slides were scanned (428 ArrayScanner, MWG Biotech, High Point, NC, USA) and analyzed using ImaGene 6.1 (BioDiscovery, El Segundo, CA, USA). Data were normalized using the Bioconductor-based CARMAWeb software package^44^ and statistically analyzed and visualized with the MulitExperiment viewer 4.8.1 from TIGR (TM4.org) using the significance analysis of microarrays algorithm as described^45, 63^.

### RNA expression relationships in human systemic lupus erythematosus

We searched three major databases (19/07/2017) GEO (26,622 human array or sequencing-based RNA datasets)^64^, ArrayExpress (70,672 datasets of 2,242,940 assays)^65^, and Gemma^66^ (4118 datasets)) data series relating to human lupus transcript analyses and individually examined more than 239 datasets. Although there are 152 data series for human lupus in GEO and 130 in ArrayExpress, only five in total had longitudinal data, which were downloaded from Gemma. We compared RNA expression levels of *MMP12* and *IFNG* genes in untreated SLE patients vs. healthy subjects in the GSE11909 dataset; and of untreated healthy reference subjects vs. treated SLE patients in the GSE37356 dataset. For each gene, microarray probes with the lowest *p*-value were selected and the multiple testing FDR-corrected *q*-values calculated by a linear modeling approach and taken directly from Gemma. All plots and analyses were done in R.

### Human lupus nephritis analyses and grade scores

Five kidney biopsies from non-SLE patients with mild to moderate arteriosclerosis and healthy, non-lupus, non-inflammatory kidney disease, and kidney samples from SLE patients were classified independently by one blinded pathologist and one nephrologist: Class I, minimal mesangial lupus nephritis; Class II, mesangial proliferative lupus nephritis; Class III, focal lupus nephritis; Class IV, diffuse segmental (IV-S) or global (IV-G) lupus nephritis; Class V, membranous lupus nephritis; Class VI, advanced sclerosing lupus nephritis. Healthy (*n* = 5) and active (A) (*n* = 5) SLE Grade III-(A), IV-Global-(A), and IV-Segmental-(A) kidney biopsies were selected for immunochemical analysis.

Glomeruli were examined and quantified for each antibody from all human biopsies of the healthy kidneys (*n* = 43 glomeruli) and SLE patient kidneys (*n* = 46 glomeruli) and scored according to these classes: Class I, normal glomeruli by light microscopy; Class II, mesangial hypercellularity or mesangial matrix expansion by light microscopy with immune deposits; Class III, active or inactive focal, segmental or global endocapillary or extracapillary glomerulonephritis involving <50% of all glomeruli; Class IV, active or inactive focal, segmental or global endocapillary or extracapillary glomerulonephritis involving >50% of all glomeruli; Class V, global or segmental subepithelial immune deposits or their morphologic sequelae by light microscopy; and Class VI, >90% of glomeruli sclerosed.

### Statistical analyses

A normal distribution of the data were shown in all cases and hence only parametric tests were required. Standard deviation and analysis of variance were used to analyze data variability. The Student’s *t*-test determined statistical significance between two treatments. A *p*-value <0.05 was considered statistically significant.

### Human ethics approvals

The UBC/Providence Health Care British Columbia Research Ethics Board approved the patient consenting procedures and research protocols for studies using human samples (approval #H15-00953), informed consent was obtained from all patients and the research was conducted in accordance with the Declaration of Helsinki.

### Data availability

All data are available from the authors upon reasonable request.

## Electronic supplementary material

Supplementary Information

Peer Review File
