# Supplementary material for: C-terminal truncation of IFN-γ inhibits proinflammatory macrophage responses and is deficient in autoimmune disease
Source: Nat Commun. 2018 Jun 20;9:2416. doi: 10.1038/s41467-018-04717-4 (PMC6010466; doi:10.1038/s41467-018-04717-4)
Supplement: Supplementary file 1 — Supplementary Information [file 41467_2018_4717_MOESM1_ESM.pdf]

Supplementary Information for "**C-terminal truncation of IFN-γ inhibits proinflammatory macrophage responses and is deficient in autoimmune disease**"

Dufour A, Bellac C, Eckhard U, Solis N, Klein T, Kappelhoff R, Fortelny N, Jobin P, Rozmus J, Mark J, Pavlidis P, Dive V, Barbour SJ & Overall CM

1- **Supplementary Figure 1.** Comparison of peripheral-blood mononuclear cell mRNA transcripts in untreated active SLE patients compared to healthy controls

2- **Supplementary Figure 2.** Comparison of peripheral-blood mononuclear cell mRNA transcripts in drug-treated SLE patients compared to healthy controls

3- **Supplementary Figure 3.** Longitudinal comparison of MMP12 and IFN-ɣ disease monitoring with a multivariate disease-activity score

4- **Supplementary Figure 4.** MMP12 cleavage analysis of human and murine interferon-ɣ

5- **Supplementary Figure 5.** ESI-Q-TOF MS analysis of human MMP12 cleavage assays of human interferon-ɣ

6- **Supplementary Figure 6.** Human MMP12 did not cleave human interleukin-4

7- **Supplementary Figure 7.** MMP12 reduced JAK-STAT1 signaling but not JAK-STAT6 signaling

8- **Supplementary Figure 8.** MMP12 dampened arthritis in a murine model of systemic lupus erythematosus

9- **Supplementary Figure 9.** Differences in lymphoid organ size and mortality of female v.s. male Mmp12$^{-/-}$ MRL/lpr mice in a murine model of SLE

10- **Supplementary Figure 10.** Blood cell counts and superficial cervical lymph node immunohistochemical analyses of Mmp12$^{+/+}$ versus Mmp12$^{-/-}$ MRL/lpr mice

11- **Supplementary Figure 11.** Anti-human IFN-γ epitope antibodies and their staining of kidney biopsies from human lupus nephritis

12- **Supplementary Figure 12.** Full Western Blots Figure 3

13- **Supplementary Figure 13.** Full Western Blots Figure 4

14- **Supplementary Figure 14.** Full Western Blots Figure 5

15- **Supplementary Figure 15.** Full Western Blots Figure 6

16- **Supplementary Figure 16.** Full Western Blots Figure 7

17- **Supplementary Figure 17.** Full Western Blots Figure 8

18- **Supplementary Table 1.** Patient diagnostic information related to the histological data shown in Figure 7 and Supplementary Figure 11

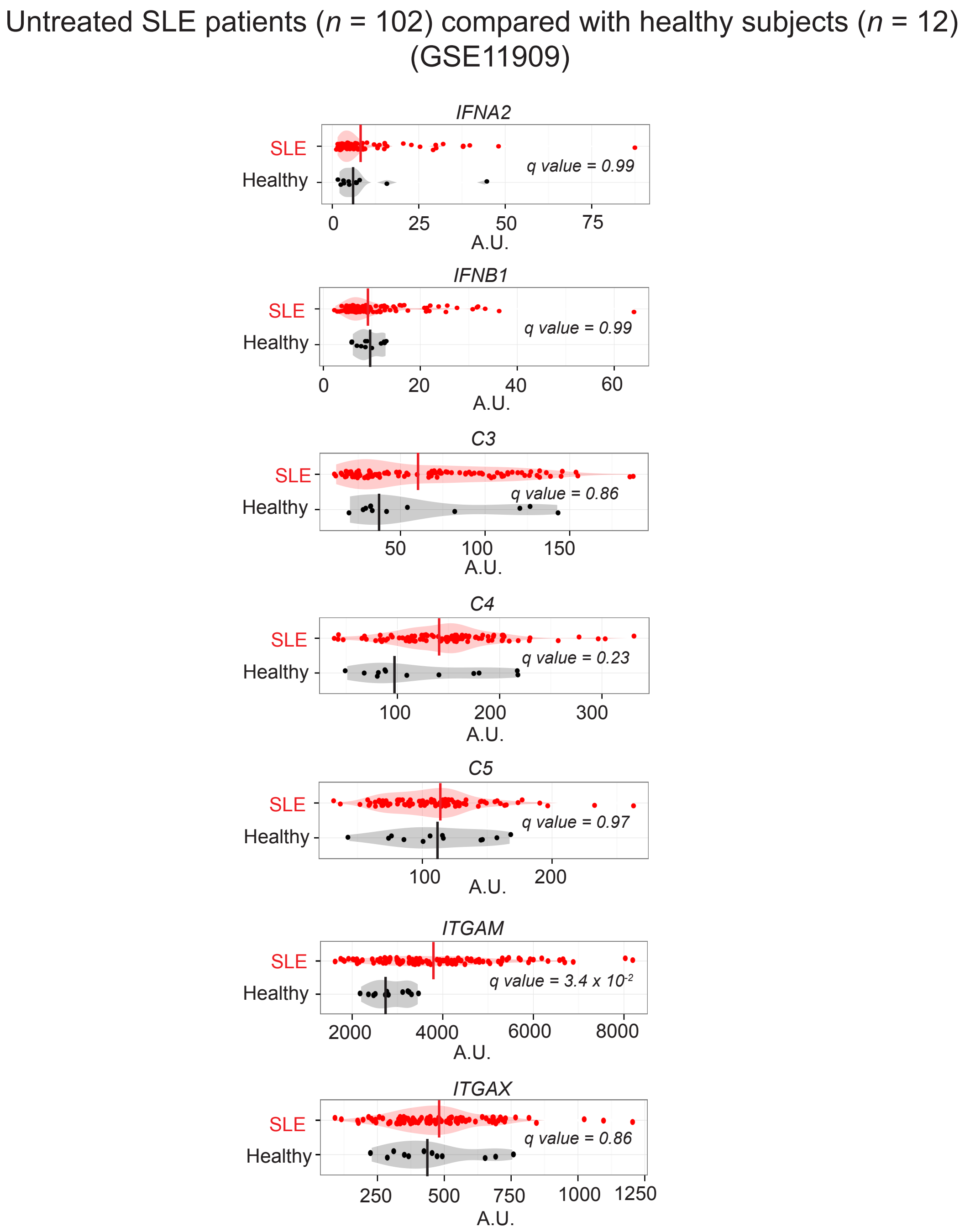

Supplementary Figure 1. Comparison of peripheral-blood mononuclear cell mRNA transcripts in untreated active SLE patients compared to healthy controls

Peripheral-blood mononuclear cell mRNA levels of *IFNA2* (Interferon alpha-2), *IFNB1* (Interferon beta), *C3*, *C4*, *C5*, *ITGAM* and *ITGAX* in SLE patients (n = 102) and healthy subjects (n = 12). Bar denotes the mean. Data taken from GSE11909 data set (Chaussabel *et al.* (2008) *Immunity* **29**, 150-164). A.U., arbitrary units.

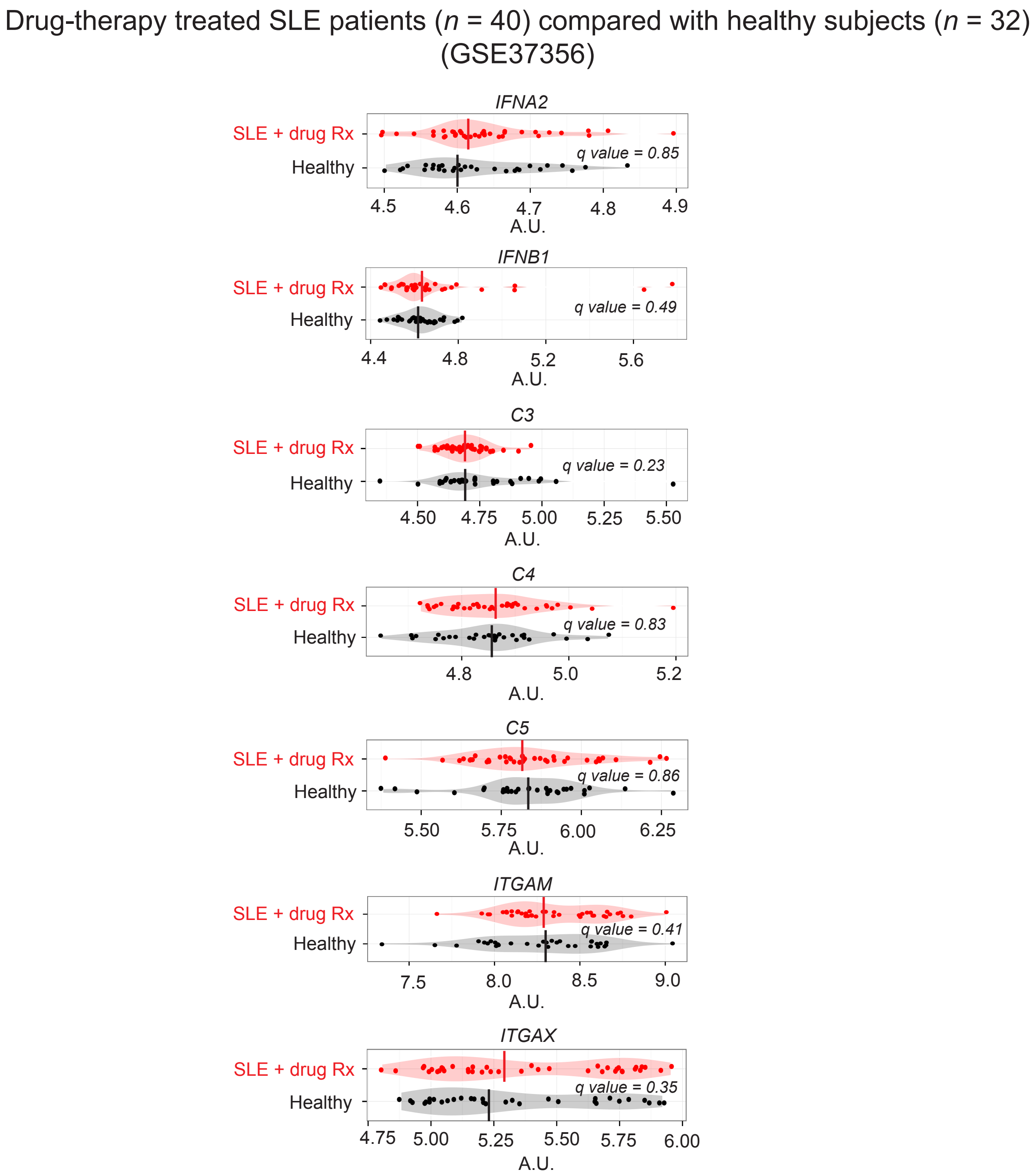

Supplementary Figure 2. Comparison of peripheral-blood mononuclear cell mRNA transcripts in drug-treated SLE patients compared to healthy controls

Peripheral-blood mononuclear cell mRNA levels of *IFNA2* (Interferon alpha-2), *IFNB1* (Interferon beta), *C3*, *C4*, *C5*, *ITGAM* and *ITGAX* in drug-treated (Rx) patients (healthy reference subjects (*n* = 32) and SLE patients (*n* = 40). Bar denotes the mean. Data taken from GSE37356 dataset (Korman *et al.* (2014) *Arthritis Res. Ther.* **16**:R147). A.U., arbitrary units.

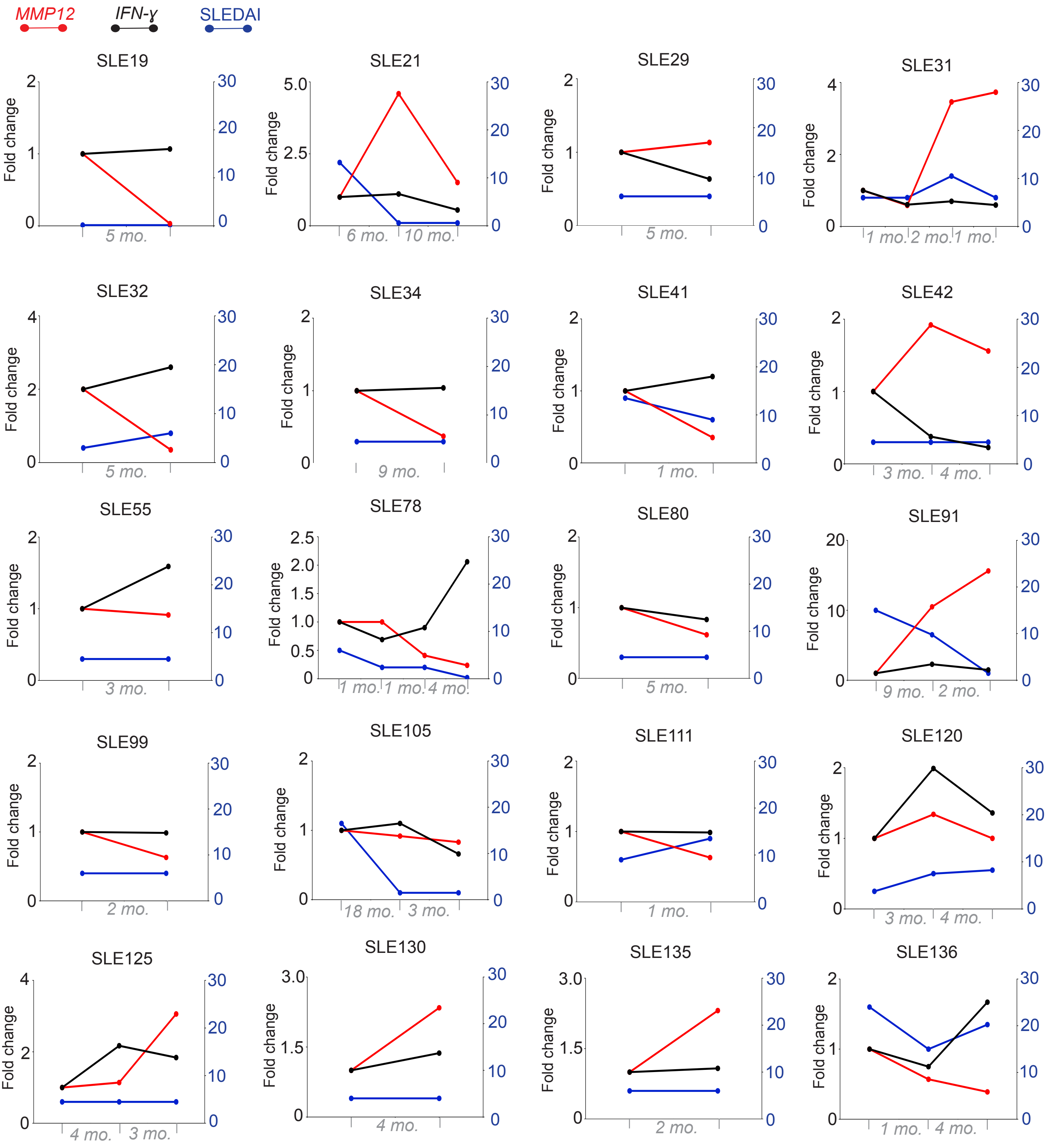

Supplementary Figure 3. Longitudinal comparison of *MMP12* and *IFN-γ* disease monitoring with a multivariate disease-activity score SLEDAI index (blue, right y axis) and transcriptional expression of *MMP12* (red) and *IFNG* (black) of SLE patients (identified by an SLE ID) over time (x axis). Time elapsed between sampling is indicated in months. Data taken from GSE11909 data set (Chaussabel *et al*. (2008) *Immunity* **29**, 150-164).

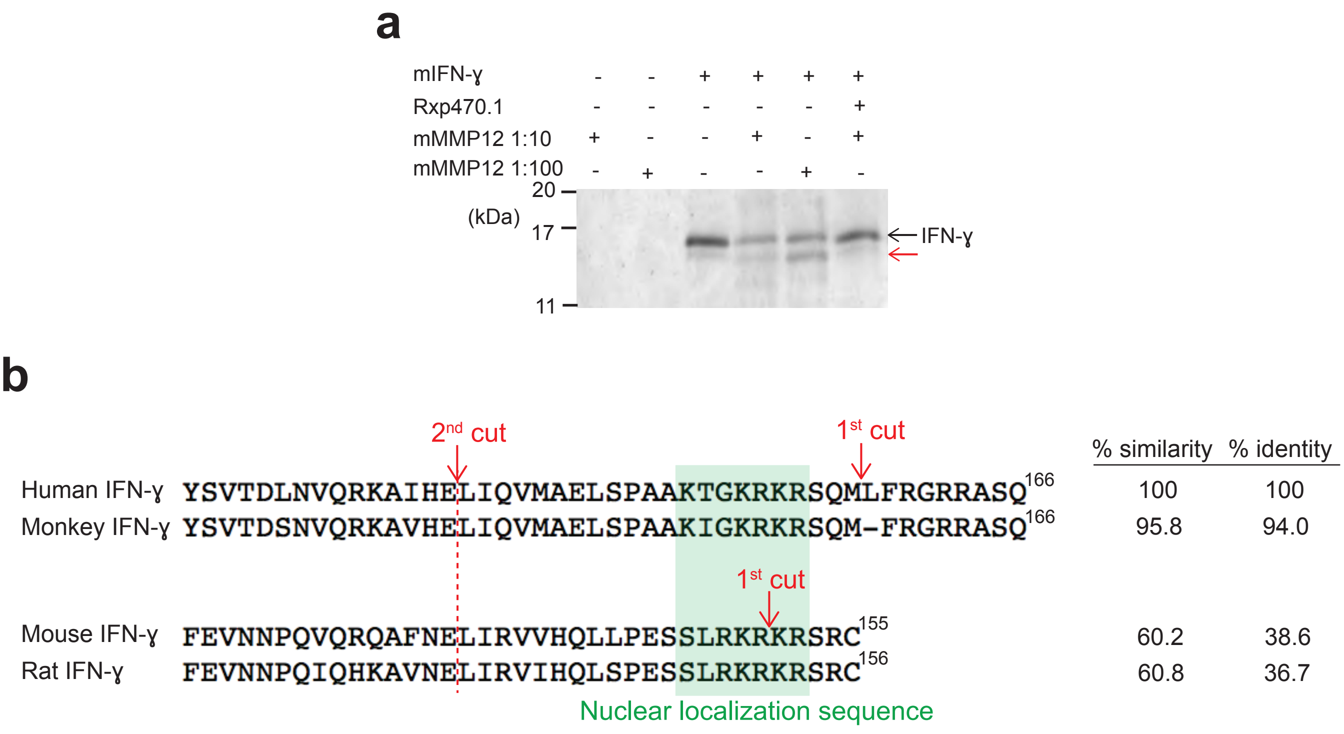

Supplementary Figure 4. MMP12 cleavage analysis of human and murine interferon-ɣ
(**a**) Silver stained 15% SDS-PAGE gel analysis of *in vitro* cleavage of recombinant murine (m) IFN-ɣ by mMMP12 (1:10 and 1:100 enzyme/substrate), red arrow indicates cleaved IFN-ɣ.
Recombinant proteins were incubated at 37 °C for 18 h. MMP12-specific inhibitor, 10 nM Rxp470.1, was also included in the assays as indicated.
(**b**) Two cleavage sites (red) of human and murine IFN-ɣ aligned with monkey and rat amino acid sequences. The nuclear localization sequence is indicated in green.

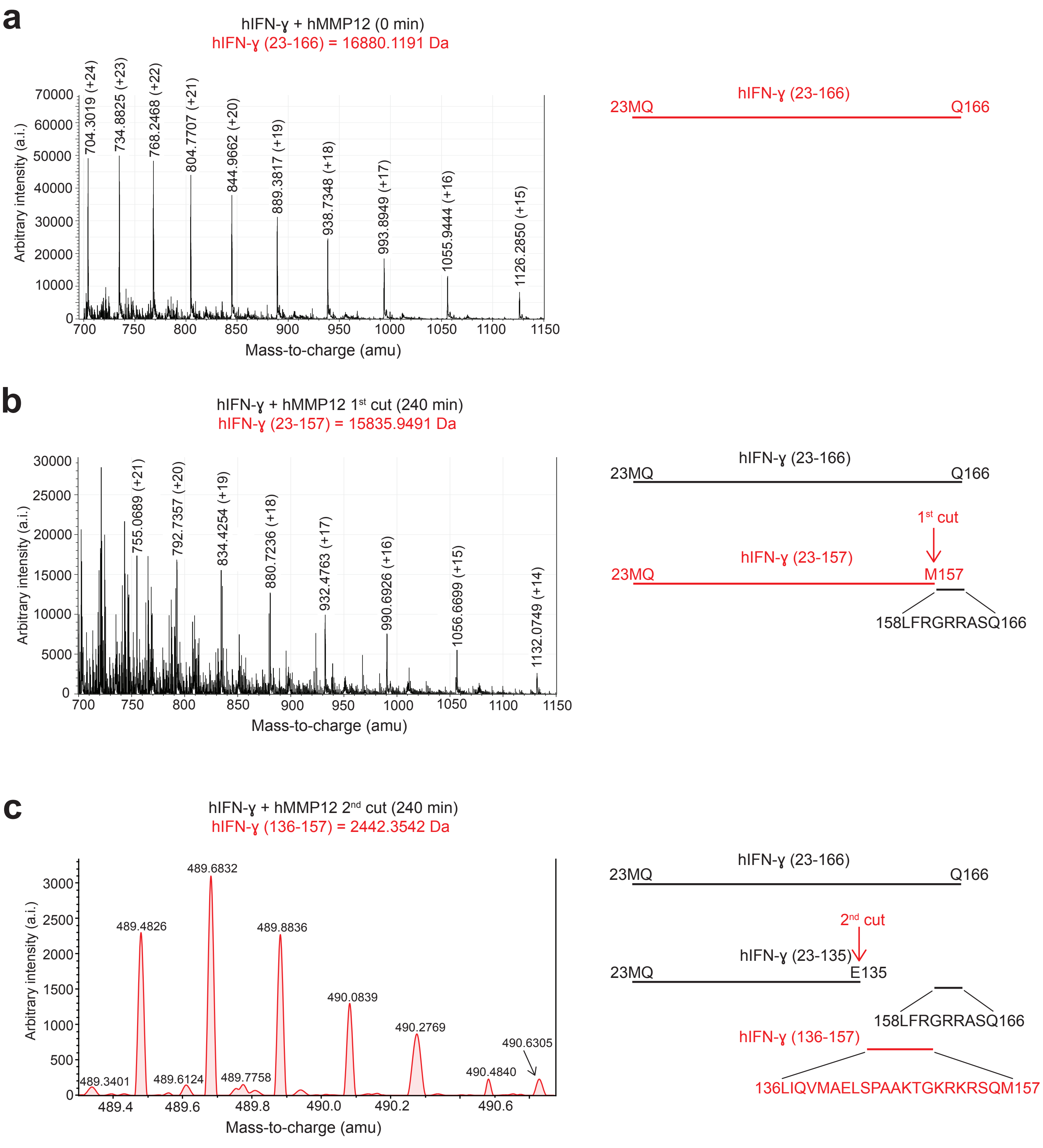

Supplementary Figure 5. ESI-Q-TOF MS analysis of human MMP12 cleavage assays of human interferon-ɣ
ESI-Q-TOF MS spectra of full length human IFN-ɣ incubated with MMP12 at (**a**) 0 min and (**b**,**c**) 240 min. The annotated spectra are of the proteins or peptides shown in red in the diagram. The mass difference between (**a**) full length, (**b**) 1st cut and (**c**) 2nd cut forms of IFN-ɣ deconvolutes to 9 amino acids and 31 amino acids truncated from the C-terminus by MMP12 cleavages at 157M↓L158 and 135E↓L136, respectively. *Note*, recombinant human IFN-ɣ commences at Met23 introduced before the natural start amino acid residue of IFN-ɣ (Gln) left after removal of the signal peptide.

a

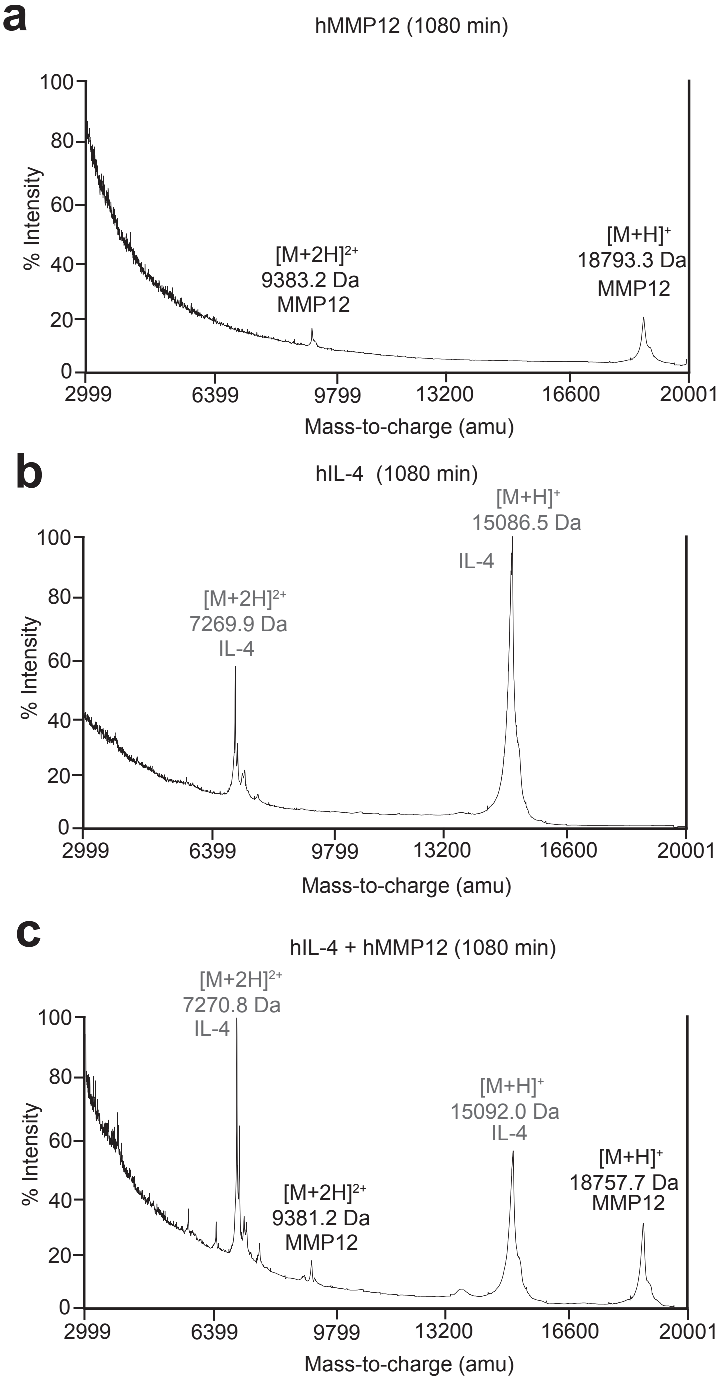

d

Time (min)	hIL-4 (24-153) m/z $[M+H]^+$ Predicted	m/z $[M+H]^+$ Measured	Δ Mass-to-charge (amu)
0	15094.4	15086.5	7.9
1080	15094.4	15092.0	2.4

e

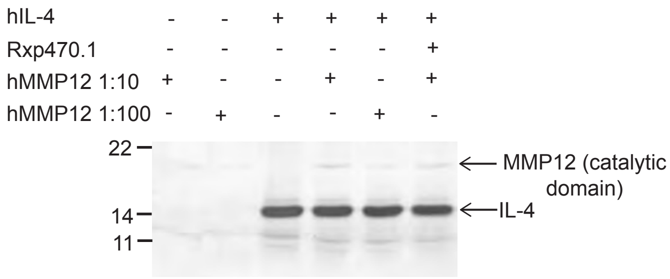

Supplementary Figure 6. Human MMP12 did not cleave human interleukin-4
MALDI-TOF MS spectra of human (**a**) MMP12 or (**b**) IL-4 incubated in the absence or (**c**) presence of MMP12 for 1080 min at 37 °C. (**d**) Spectral peaks were assigned by comparison of measured with predicted mass to charge ratios (*m/z*) of peaks with a +1 charge ($[M + H]^+$) or +2 charge ($[M + 2H]^{2+}$). (**e**) Silver stained 15% SDS-PAGE gel analysis of *in vitro* cleavage assay of IL-4 by MMP12 (1:10 and 1:100 enzyme/substrate). Recombinant proteins were incubated at 37 °C for 18 h. MMP12-specific inhibitor, 10 nM Rxp470.1, was also included in the assays as indicated. No cleavage of IL-4 was detected.

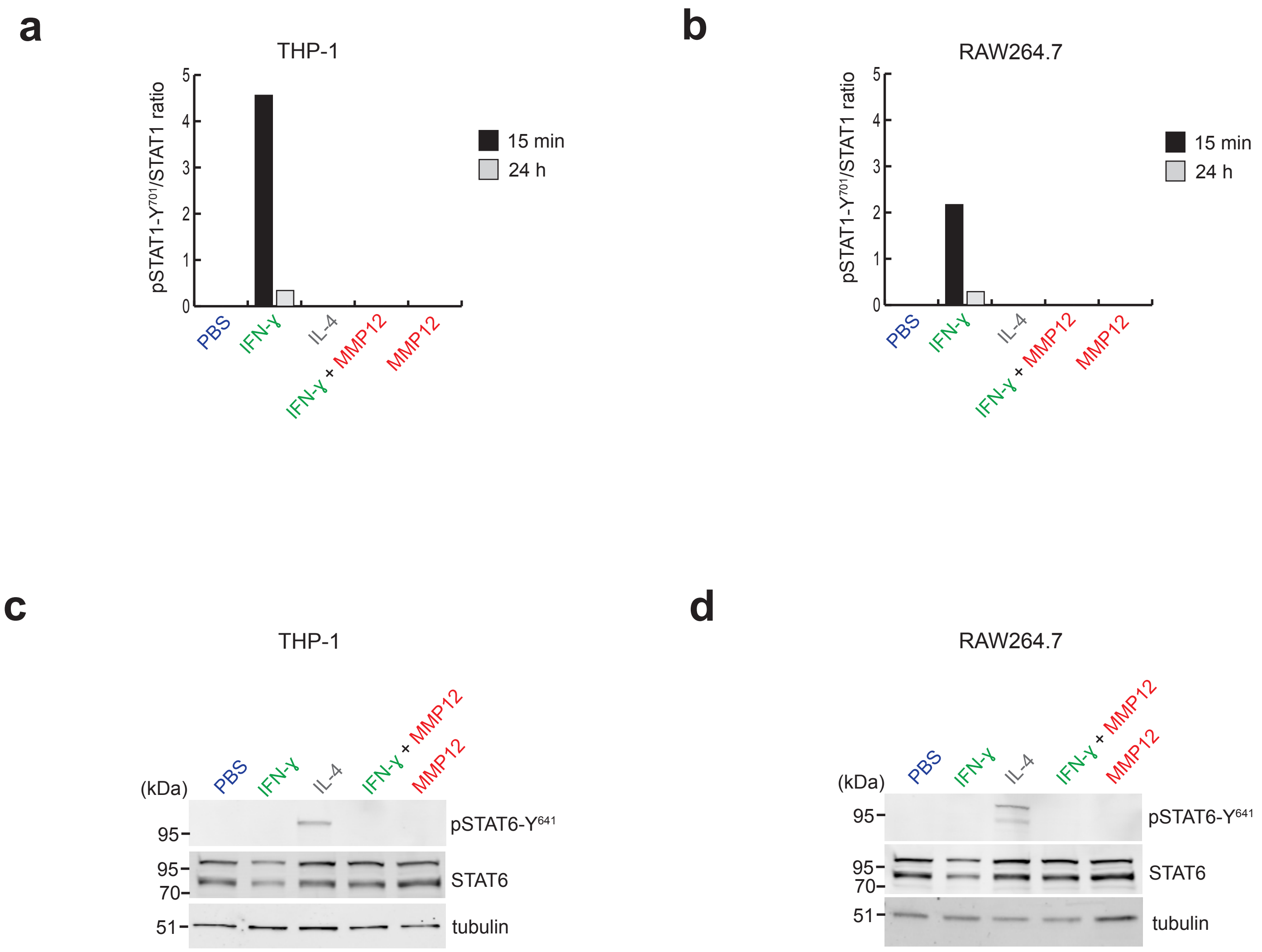

Supplementary Figure 7. MMP12 reduced JAK-STAT1 signaling but not JAK-STAT6 signaling
(**a**) Quantification of western blot analysis of pSTAT1-Y^{701} over total levels of STAT1 protein in human PMA-treated THP-1 monocytes and (**b**) murine RAW264.7 cells for 15 min and 24 h from **Figure 4a,e**. Cells were treated with either PBS, 30 ng/mL IL-4, 20 ng/mL IFN-γ, and/or 2 ng/mL MMP12 for 15 min or 24 h before lysis and samples electrophoresed on 10% SDS-PAGE and blotted with the antibodies shown. Tubulin was used as a loading control. (**c**) Western blot analysis of pSTAT6-Y^{641} and STAT6 in human PMA-matured THP-1 monocytes and (**d**) murine RAW264.7 cells. Cells were treated with either PBS, 30 ng/mL IL-4, 20 ng/mL IFN-γ, and/or 2 ng/mL MMP12 for 15 min before lysis and samples were electrophoresed on 10% SDS-PAGE and blotted with antibodies against the proteins indicated. Tubulin was used as a loading control.

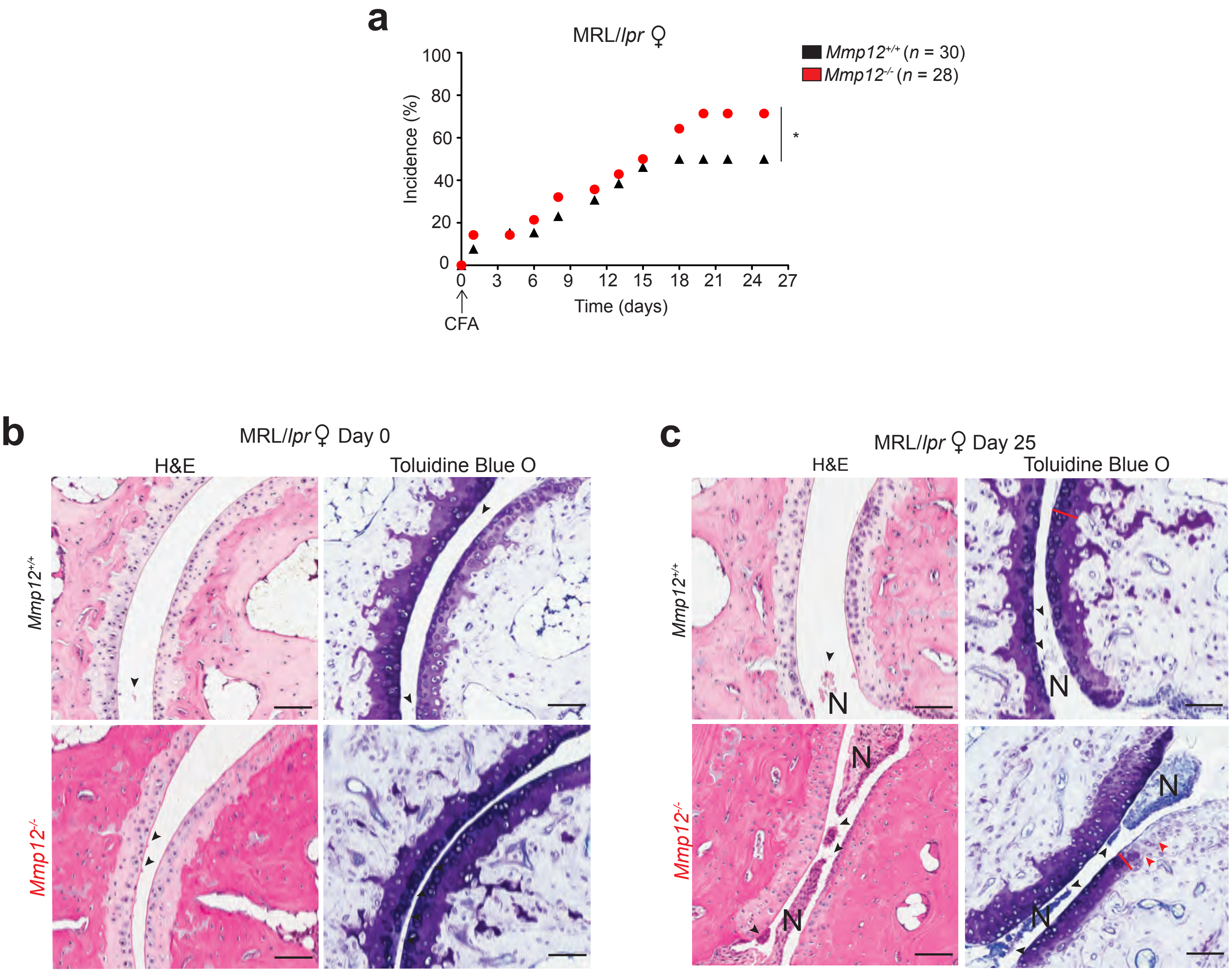

Supplementary Figure 8. MMP12 dampened arthritis in a murine model of systemic lupus erythematosus
(**a**) Incidence of joint inflammation after Complete Freund's Adjuvant (CFA) injection in female *Mmp12*$^{+/+}$ (*n* = 30) and *Mmp12*$^{-/-}$ (*n* = 28) MRL/*lpr* mice (*, $p < 3 \times 10^{-2}$). Statistical analysis was determined by a two-tailed unpaired Student's t test. Original magnification, x400. (**b**) Histological analysis of hind ankle joints at day 0 in female *Mmp12*$^{+/+}$ (*n* = 3) and *Mmp12*$^{-/-}$ (*n* = 3) MRL/*lpr* mice. Minimal synovial inflammatory cell infiltration (arrow heads) was shown by hematoxylin and eosin (H&E) (upper and lower left panels) and with no cartilage damage shown by Toluidine blue O staining for proteolgycan (upper and lower right panels). (**c**) Histological analysis of hind ankle joints at day 25 post-CFA injection in *Mmp12*$^{+/+}$ (*n* = 3) and *Mmp12*$^{-/-}$ (*n* = 3) MRL/*lpr* mice. Moderate synovial inflammatory cell infiltration was seen using hematoxylin and eosin (H&E) (upper left panel) and with mild cartilage damage shown by Toluidine blue O staining (upper right panel). *Mmp12*$^{+/+}$ mice showed low amounts of neutrophils and NETs (N). *Mmp12*$^{-/-}$ mice at day 25 showed massive inflammatory infiltration including invasion of the joint and marrow spaces (lower left panel), large deposits of neutrophils and NETs, and marked loss of joint architecture and proteoglycan content (lower right panel).

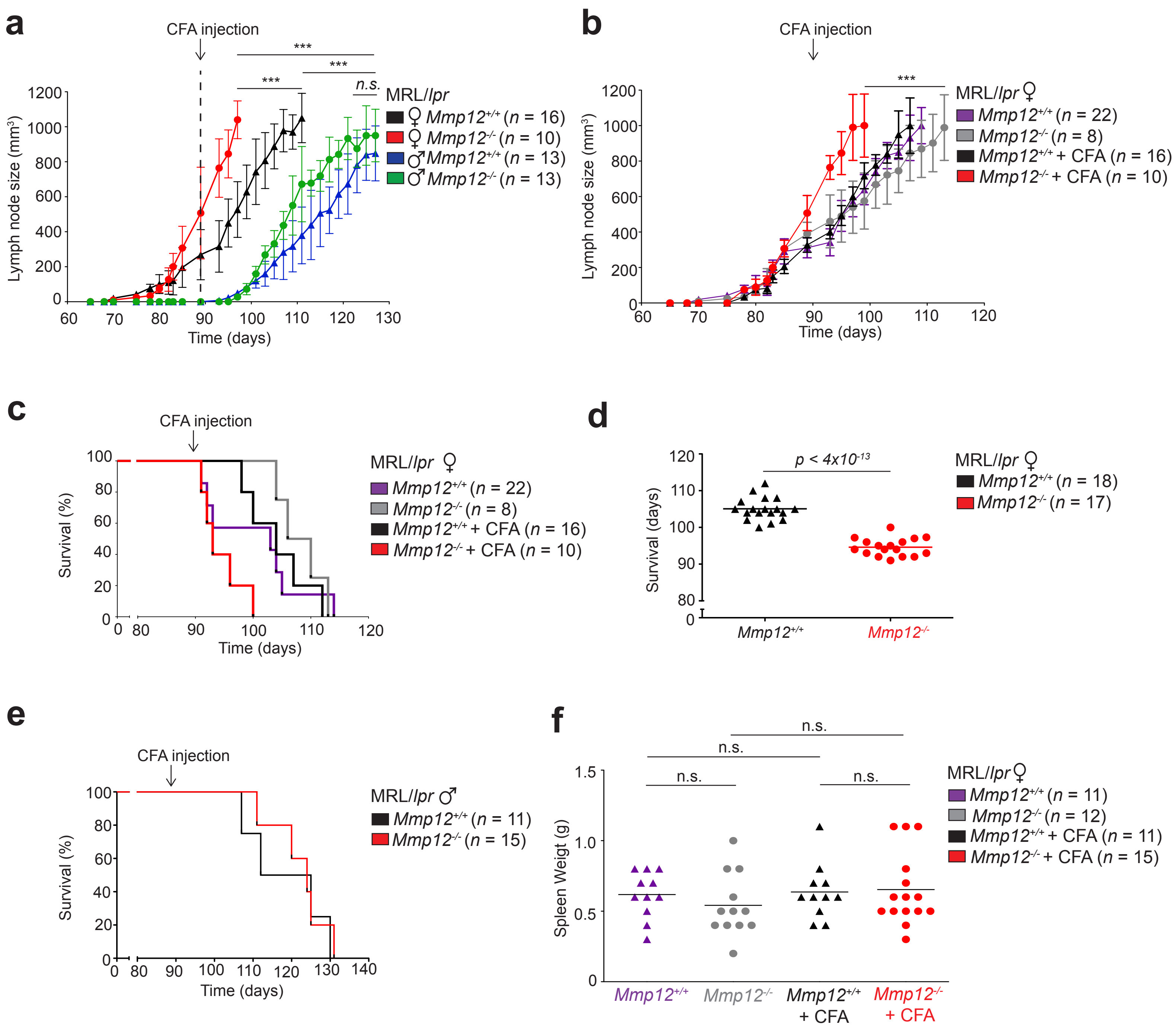

Supplementary Figure 9. Differences in lymphoid organ size and mortality of female *v.s.* male *Mmp12*$^{-/-}$ MRL/*lpr* mice in a murine model of SLE
(**a**) The lymph node size of CFA-treated male *Mmp12*$^{+/+}$ MRL/*lpr* (n = 13) and *Mmp12*$^{-/-}$ MRL/*lpr* (n = 13) mice *versus* CFA-treated female *Mmp12*$^{+/+}$ MRL/*lpr* (n = 16) and *Mmp12*$^{-/-}$ MRL/*lpr* (n = 10) (N = 2) mice; (**b**) The lymph node size and (**c**) the survival of untreated *Mmp12*$^{+/+}$ MRL/*lpr* (n = 22) and *Mmp12*$^{-/-}$ MRL/*lpr* (n = 8) female mice compared in a biological replicate experiment to CFA-treated *Mmp12*$^{+/+}$ (n = 16) and *Mmp12*$^{-/-}$ MRL/*lpr* (n = 10) female mice.
(**d**) The median survival of female *Mmp12*$^{+/+}$ MRL/*lpr* (n = 18) and *Mmp12*$^{-/-}$ MRL/*lpr* (n = 17) mice after injection of complete Freund's adjuvant (CFA) (N = 2).
(**e**) Kaplan-Meier curve showing no differences in mortality rates of male *Mmp12*$^{+/+}$ (n = 11) and *Mmp12*$^{-/-}$ (n = 15) mice after CFA induction (N = 2).
(**f**) Spleen weight of *Mmp12*$^{+/+}$ (n = 11) and *Mmp12*$^{-/-}$ (n = 12) female mice without CFA induction or *Mmp12*$^{+/+}$ (n = 11) and *Mmp12*$^{-/-}$ (n = 15) female mice after CFA induction. No changes in spleen weights were apparent. Statistical analysis was determined by a two-tailed unpaired Student's t test.
n.s., not significant.

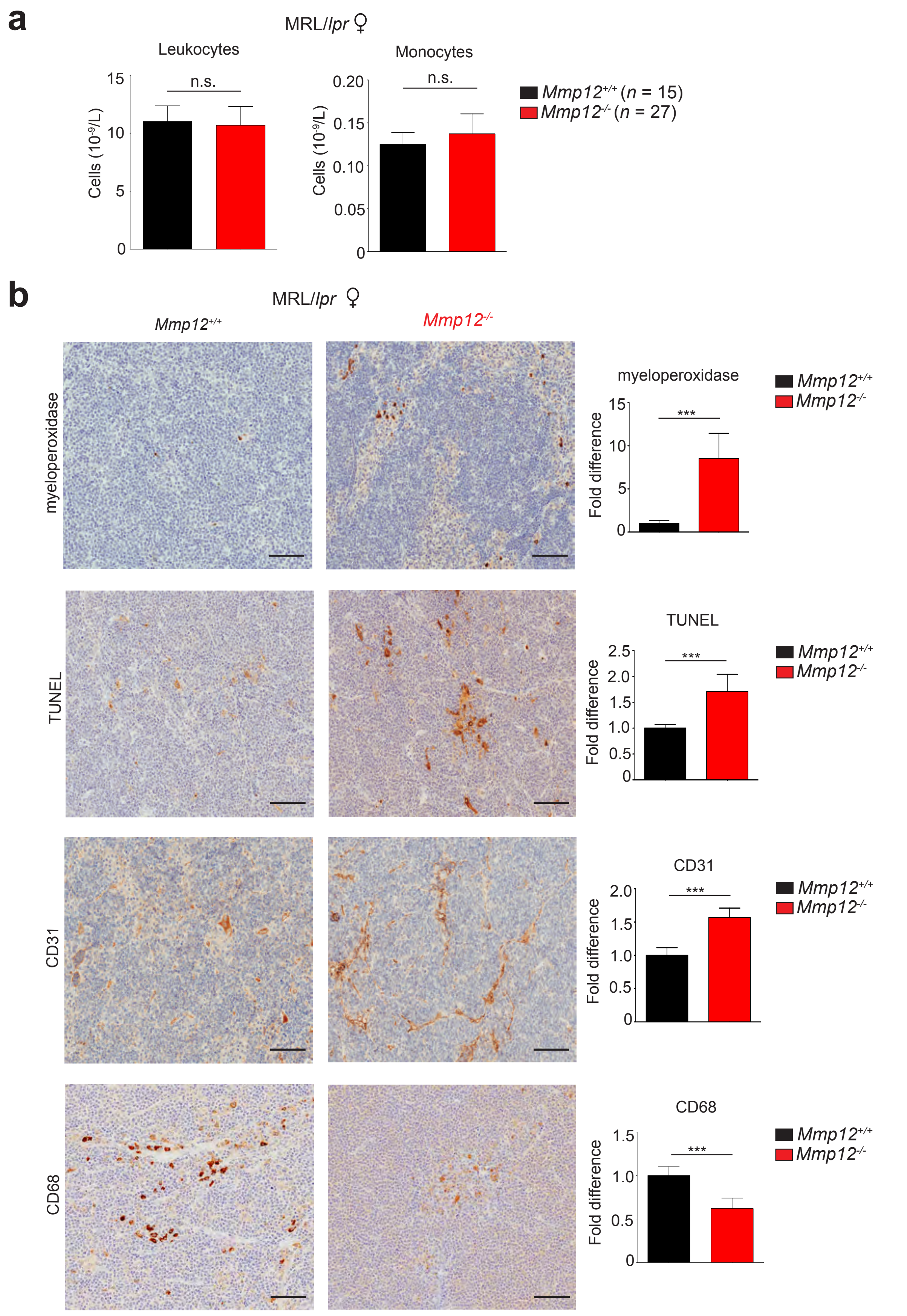

Supplementary Figure 10. Blood cell counts and superficial cervical lymph node immunohistochemical analyses of *Mmp12*$^{+/+}$ *versus Mmp12*$^{-/-}$ MRL/*lpr* mice

(**a**) Blood was collected from 90-day old *Mmp12*$^{+/+}$ (*n* = 15) and *Mmp12*$^{-/-}$ (*n* = 27) MRL/*lpr* mice and the total numbers of leukocytes and monocytes were analyzed in technical duplicates. (**b**) Myeloperoxidase, TUNEL, CD31 and CD68 immunostaining of the superficial cervical lymph nodes of *Mmp12*$^{+/+}$ (*n* = 3) and *Mmp12*$^{-/-}$ (*n* = 3) MRL/*lpr* mice. Scale bars, 100 µm. Quantification of immunostaining intensities of *Mmp12*$^{+/+}$ in comparison to *Mmp12*$^{-/-}$ mice superficial cervical lymph nodes for myeloperoxidase (***, $p < 2 \times 10^{-11}$), TUNEL (***, $p < 6 \times 10^{-5}$), CD31 (***, $p < 1 \times 10^{-4}$), and CD68 (***, $p < 2 \times 10^{-6}$) was performed using Aperio Image Scope v12.1.0 software.Statistical analysis was determined by a two-tailed unpaired Student's t test. Original magnification, x400.

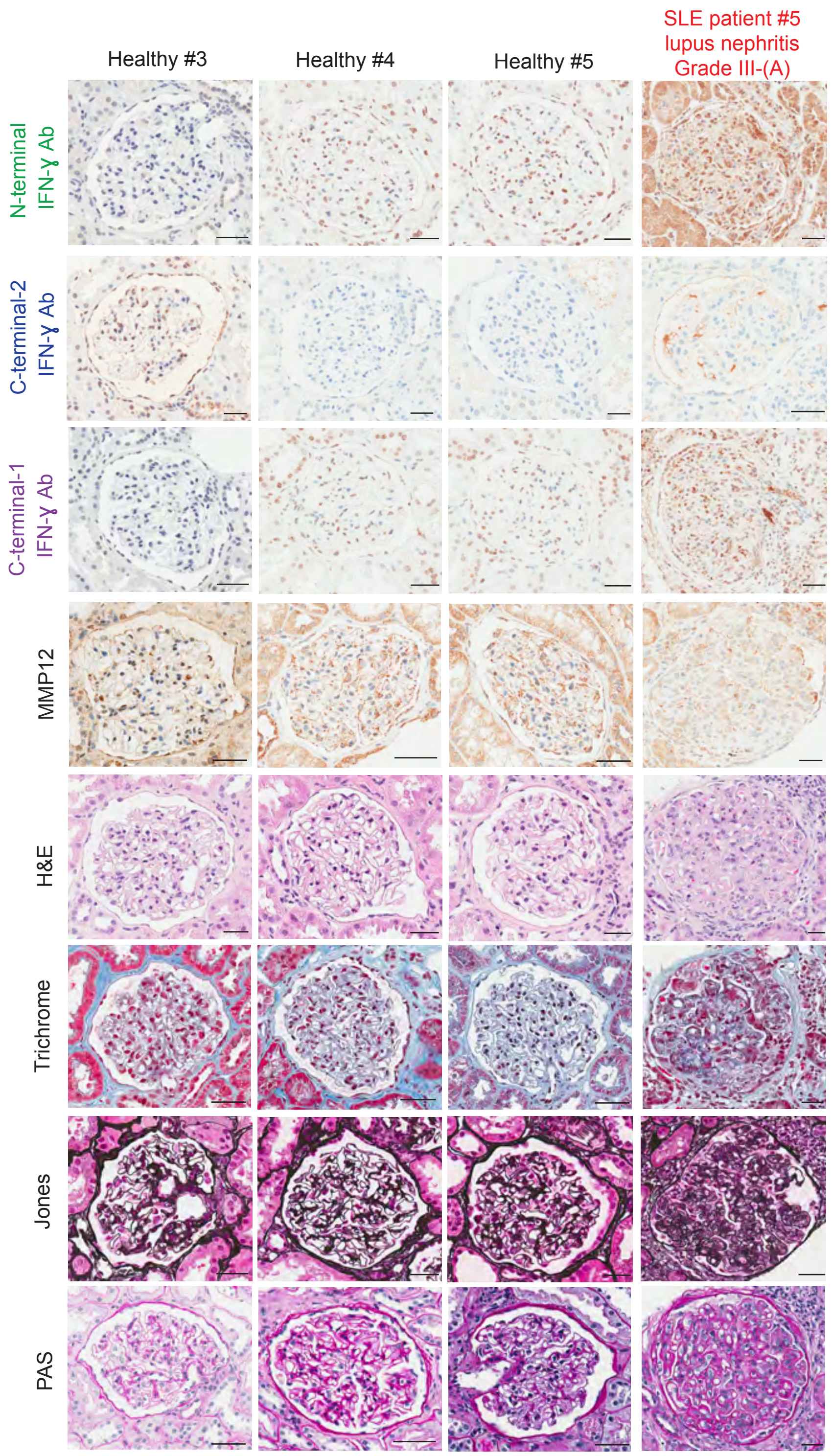

Supplementary Figure 11. Anti-human IFN-γ epitope antibodies and their staining of kidney biopsies from human lupus nephritis
Immunostaining of human kidney using anti–N-terminal, C-terminal-2, C-terminal-1, and MMP12 antibodies; and staining with hematoxylin and eosin (H&E), Masson’s trichrome, Jones, and PAS of additional healthy kidney biopsies (*n* = 5) and of lupus nephritis (*n* = 5) at stage III-(A) from **Figure 7**. Scale bar, 100 µm. Original magnification, x400.

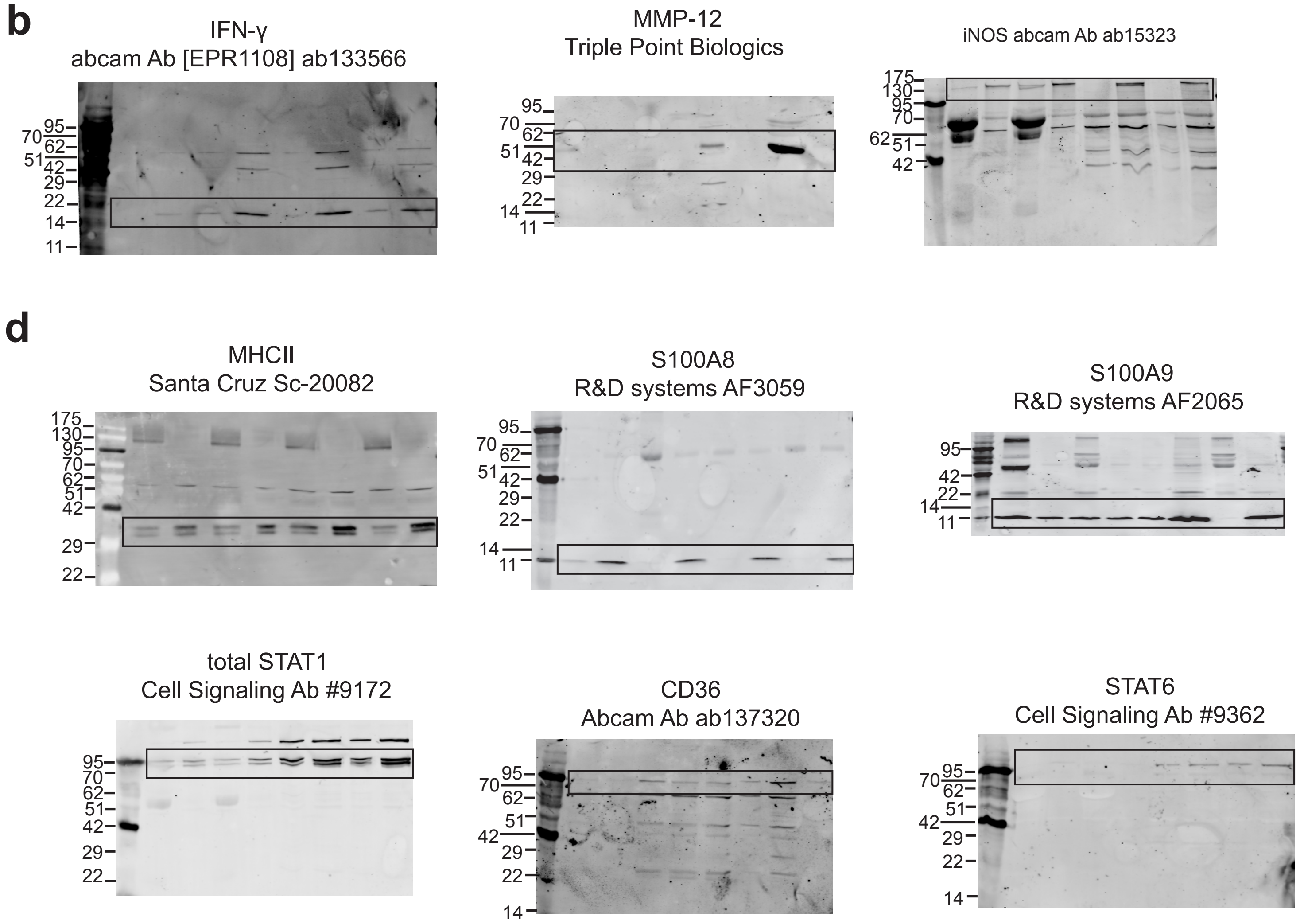

Supplementary Figure 12. Figure 3 full Western blots

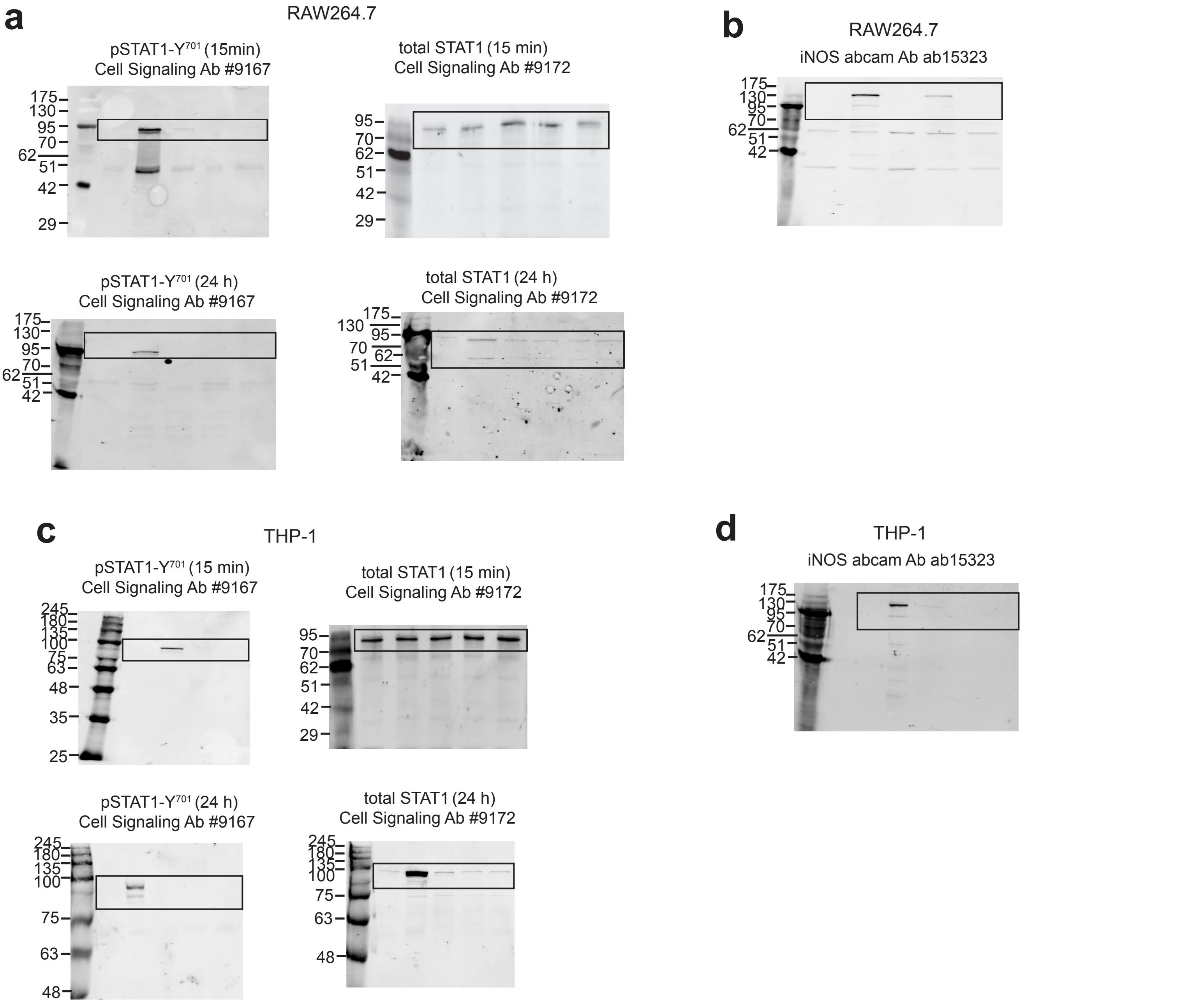

Supplementary Figure 13. Figure 4 full Western blots

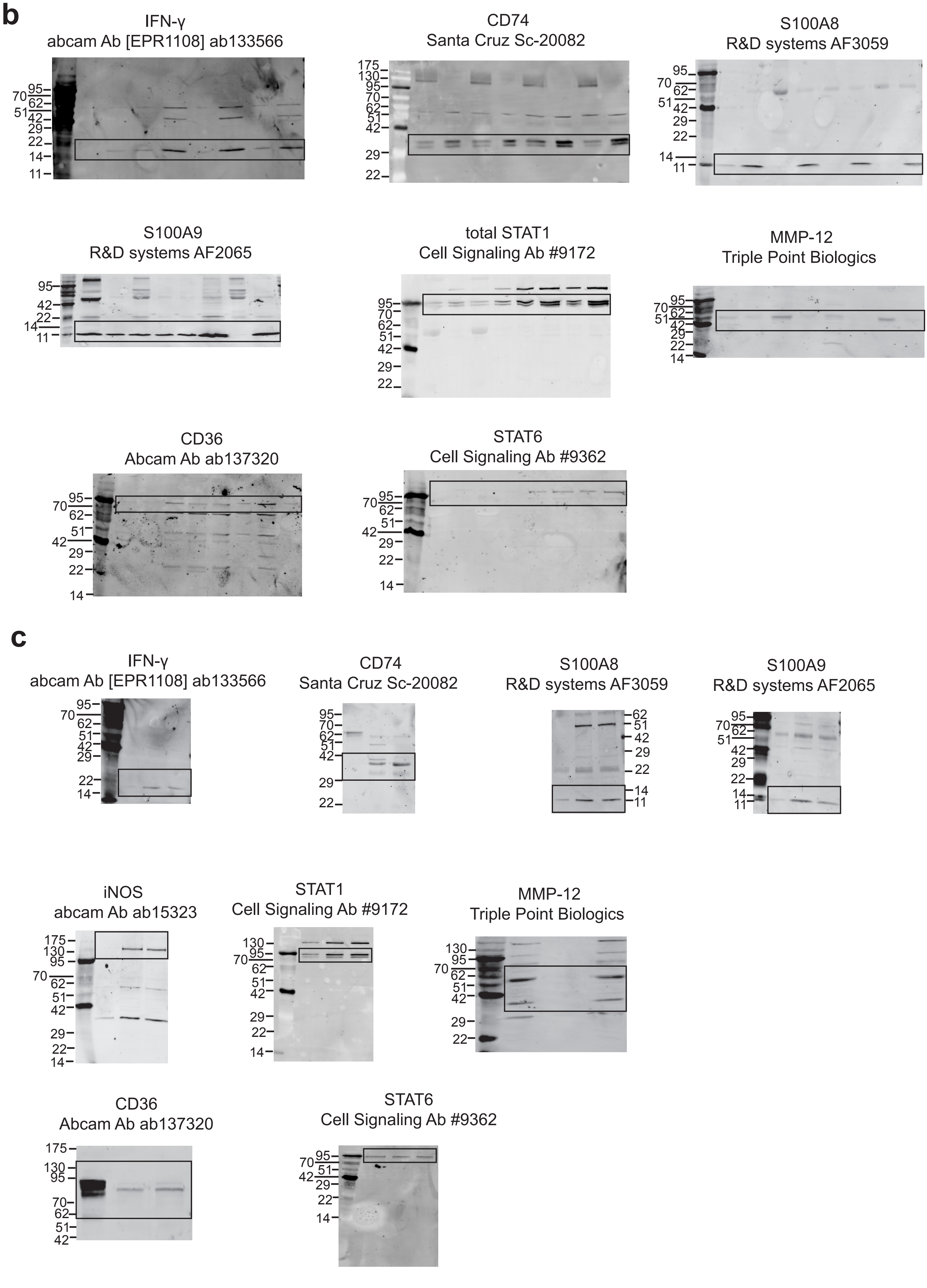

Supplementary Figure 14. Figure 5 full Western blots

e

MRL/*lpr* lymph nodes

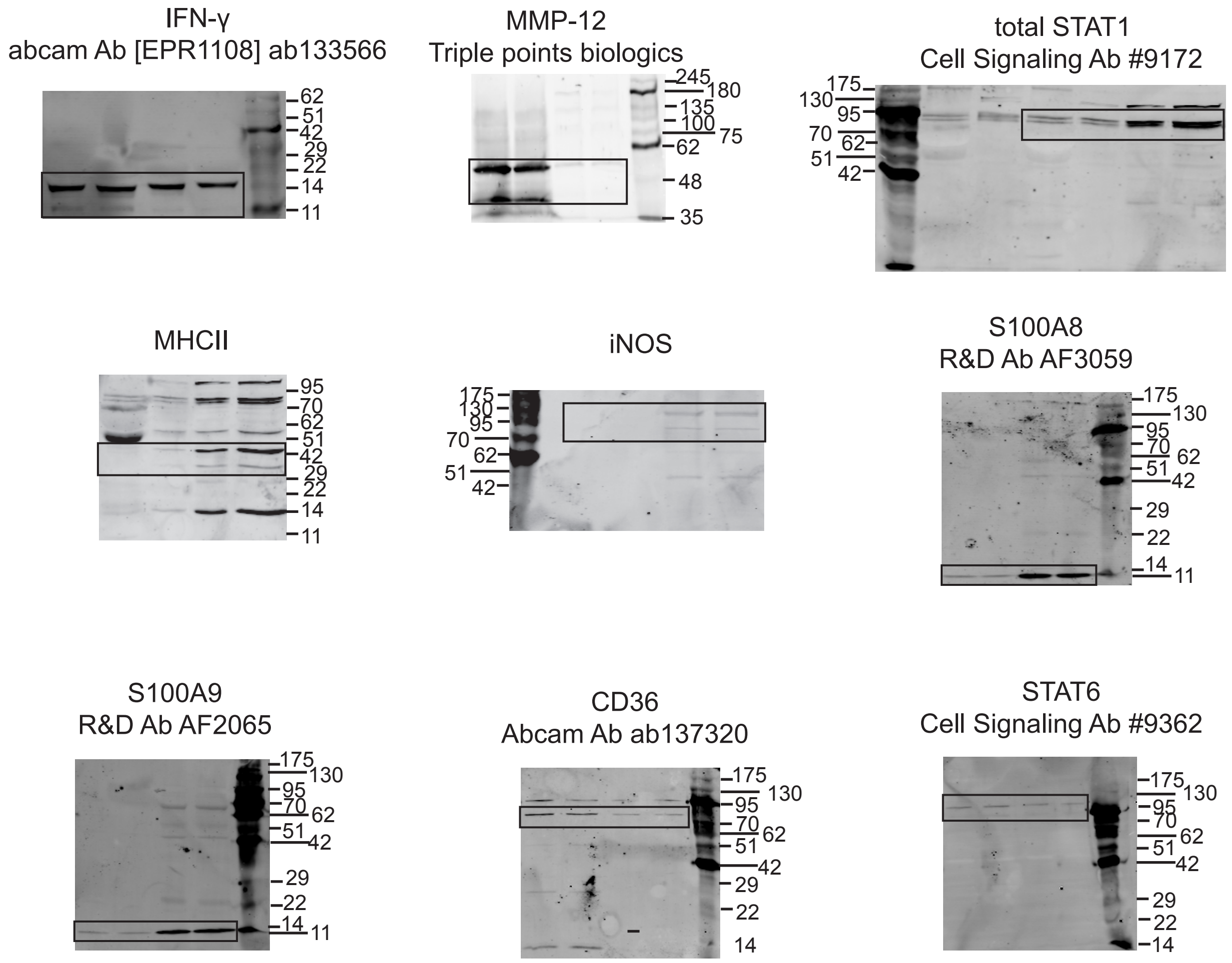

Supplementary Figure 15. Figure 6 full Western blots

a

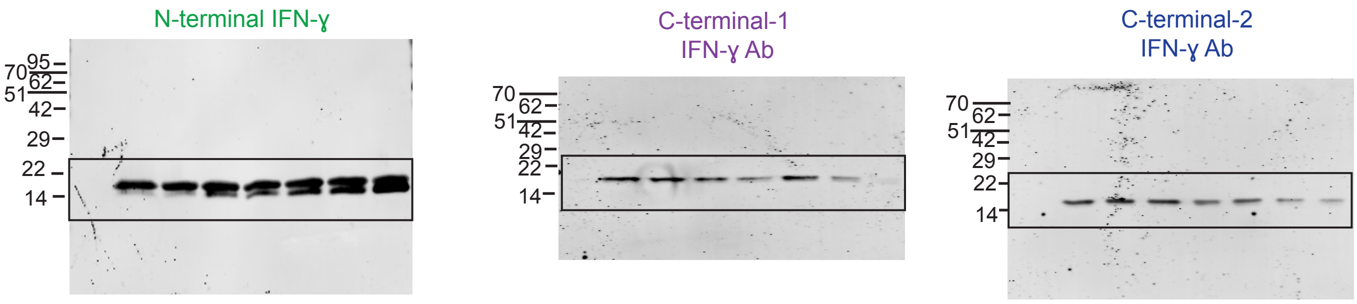

Supplementary Figure 16. Figure 7 full Western blots

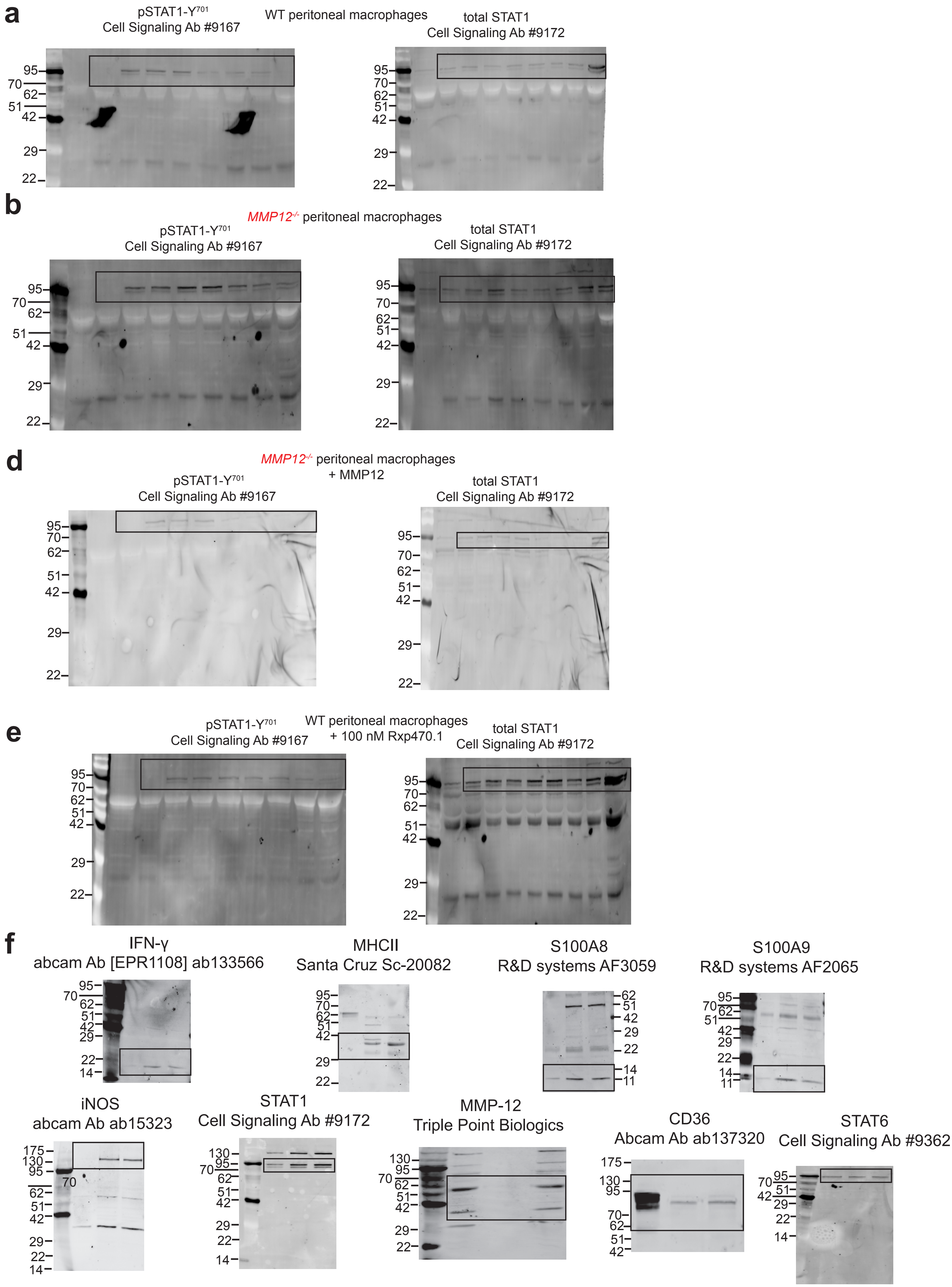

Supplementary Figure 17. Figure 8 full Western blots

SLE patient information

Supplementary Table 1. Patient diagnostic information related to the histological data shown in Figure 7 and Supplementary Figure 11

Patient (number)	1	2	3	4	5
Lupus nephritis SLE class	**III -(A)**	**III-(A)**	**IV-G-(A)**	**IV-S-(A)**	**III-(A)**
Creatinine at biopsy	74	67	80	161	-
Glomerular filtration rate (GFR) at biopsy	91	111	68	50	-
Albumin/creatinine ratio (ACR) at biopsy	89.7	11.4	-	256	-
24 h proteinuria at biopsy	-	-	10.07	9.48	-
Erythrocyte sedimentation rate (ESR) at biopsy (normal range < 20 mm/hr)	-	50	-	-	-
C-reactive protein (CRP) at biopsy (normal range < 5.0 mg/L)	33.7	16.3	-	8.6	-
C3 at biopsy (normal range 0.90-1.90 g/L)	0.45	0.65	0.39	0.39	-
C4 at biopsy (normal range 0.13-0.46 g/L)	0.03	0.1	0.03	0.08	-
dsDNA at biopsy (normal range < 100)	452	271	35	>300	-

SLE patient	1	2	3	4	5
Biopsy details	ISN/RPS Class III-(A) with active focal proliferative lupus glomurelonephritis. The activity is in the form of segmental endocapillary hypercellularity in about 1/3 of the glomureli present in histologic sections. Some of the glomureli showing active segmental lesions also contain small cellular or fibrocellular crescents.	ISN/RPS Class III-(A) with active focal lupus glomurelonephritis. About 20 to 25% of the glomureli show active lesions, mostly in the form of segmental endocapillary hypercellularity.	ISN/RPS Class IV-G-(A) with diffuse endocapillary proliferative glomerulonephritis with significant lobulation of glomurela tufts.	Moderately active diffuse proliferative lupus glomerulonephritis (ISN/RPS Class IV-S-(A)). The activity is in the form of segmental endocapillary proliferation and large circumferential eosinophilic deposits along capillary walls ("wire loop" lesions). This is accompanied by focal acute tubular injury. About 10% of the cortex shows chronic tubular atrophy and insterstitial fibrosis.	Moderately active focal proliferative lupus glomerulo-nephritis (ISN/RPS Class III-(A)). 20-25% of glomeruli in histologic and Toluidine-stained sections show segmental endocapillary hypercellularity. No chronic tubulointerstitial change is noted in sections.
