# Supplementary material for: C-terminal truncation of IFN-γ inhibits proinflammatory macrophage responses and is deficient in autoimmune disease
Source: Nat Commun. 2018 Jun 20;9:2416. doi: 10.1038/s41467-018-04717-4 (PMC6010466; doi:10.1038/s41467-018-04717-4)
Supplement: Supplementary file 2 — Peer Review File [file 41467_2018_4717_MOESM2_ESM.pdf]

Reviewers' comments:

Reviewer #1 (Remarks to the Author):

Interferon-gamma (IFNg) is a central mediator in regulating the activation status of macrophages and their contribution to inflammation. In this paper, Dufour et al describe a novel and potentially interesting mechanism that pertains to the potential of MMP12 to cleave IFNg, and thus curb inflammatory macrophage activity. Further studies then relate to how disruption of this mechanistic pathway may contribute to autoimmune diseases, such as systemic lupus erythematus (SLE; lupus) and rheumatoid arthritis, both in humans and mouse models.

The study is detailed, and the paper is well written. The authors produced a significant body of work which is generally supportive of their hypothesis. Nevertheless, addressing the points below is highly recommended to significantly improve the robustness of the results and the impact of the work.

Major comments:

1. Cohorts

a. Major conclusions made by Dufour et al pertain to the relevance of the described mechanism to SLE, and that MMP12 protects from the development of active disease (page 4). If so, demonstrating that untreated active lupus is associated with decreased MMP12 in one cohort, and that treated SLE patients have comparable MMP12 levels to healthy controls in a totally separate cohort, is not sufficiently convincing. A longitudinal cohort including the same patients studied over time (e.g. active vs. treated) is required, and should not be particularly challenging to obtain. There are many lupus cohorts available to study, and if not lupus patients are not rare in rheumatology units. This would also allow more complete characterization of the cohorts from the point of view of important clinical and epidemiological features (age, sex, duration of disease, disease manifestations, etc). Moreover, this approach could potentially strengthen a causation argument, showing that MMP12 normalization occurs early after instituting treatment and precedes clinical improvement, while patients whose MMP12 levels are not affected do not improve.

b. The number of mice studied for many experiments (as subsets of larger cohorts), as well as the number of lupus biopsies stained, is not sufficient. It is difficult to make any solid conclusions from n=3 in each group, as was sometimes used.

2. Specificity: It is not clear from the design of the study whether this mechanism is specific for lupus (in human disease) or inflammatory arthritis (collagen or CFA induced in mice), or is more broadly applicable. First, since the animal models involve arthritis, analyzing MMP12 levels in a cohort of patients with rheumatoid arthritis is indicated together with the lupus cohort analysis. Furthermore, besides the comparison to normal individuals, including a cohort of patients with chronic non-inflammatory disease (e.g. diabetes, osteoarthritis) is important. Staining kidneys of patients with non-lupus, inflammatory and non-inflammatory renal disease in Figure 7 is important as well.

To clarify, showing for example that patients with untreated rheumatoid arthritis also have significantly reduced MMP12 expression would not decrease from the significance of paper and the findings in lupus. It is, however, important to determine whether the proposed mechanism is limited to a single autoimmune disease or phenotype.

3. MRL/lpr experiments: One major advantage of the MRL-lpr/lpr strain which makes it highly suitable for modeling human disease is the spontaneous onset of the autoimmune and clinical phenotype. Indeed, the large majority of the lupus relevant studies in this strain involve unmanipulated mice. Moreover, spontaneous arthritis is a feature of MRL-lpr/lpr mouse. It is therefore not clear why the authors chose a CFA-accelerated model in wild type and MMP12 KO mice, and furthermore what are the relevance of any results obtained to spontaneous lupus. Moreover, even in the CFA-triggered experimental setup, central features of this model were not studied or provided (proteinuria, circulating anti-nuclear/anti-DNA autoantibodies). It is recommended that the authors study the spontaneous development of disease in the MMP12 wild type and sufficient MRL/lpr mice, including better characterization of renal disease, circulating antibodies and autoantibodies, spleen/lymph node weight, and survival.

Minor comments:

a. What is the physiological function of MMP12? It is mentioned that it has “established mechanisms in regulation of macrophage activation and to other anti-inflammatory activities”, but what are these? Could any of the results reported be due to other effects of MMP12, besides cleavage of IFNg?

b. What is the phenotype of MMP12 KO mice? Furthermore, is there a difference in IFNg related genes at baseline? (Figure 3A).

c. Is it possible that other IFNs are also being targeted by MMP12? e.g., besides showing that MMP12 doesn't cleave IL-4 (Supp. Figure 5), what is the effect on type I interferon?

d. In some of the in vitro studies, it would be interesting to include an additional control such as LPS which stimulates M1 type macrophages via STAT1, but is independent of IFNg. It is then possible to demonstrate the specificity of MMP12 affecting macrophages via IFNg related pathways.

e. Is it standard practice to demonstrate NETs with H&E staining?

f. The conclusion reached in the figure legend of Supplemental figure 8 about auto-reactive B cells is not justified without at least looking at circulating levels of autoantibodies.

g. Additional detail on the scoring of the kidney histology sections would be beneficial. How many glomeruli were examined? What parameters were scored? Moreover, both H+E and IHC (figure 6i) demonstrate peri-glomerular/interstitital disease as well. Tubulo-interstitial disease should be quantitated separately from glomerular disease, whether or not both were included in the glomerulonephritis score provided in figure 6g.

Reviewer #2 (Remarks to the Author):

This manuscript elegantly demonstrates the importance of IFN-γ regulation during cellular inflammatory response. The authors found an inbuilt negative feedback loop regulating IFN-γ activity that correlates with a decrease in proinflammatory markers. Specifically, they found that macrophage-specific matrix metalloproteinase 12 (MMP12) cleaves IFN-γ, which renders the ligand inactive, highlighting the importance of post-translational cleavage events in the regulation of immune responses. To identify the cleavage sites, the authors used a top-down mass spectrometry approach and identified the C-terminal truncations to occur at 157Met↓Leu158 and 135Glu↓Leu136. To characterize the role of truncated IFN-γ , mouse (RAW264.7) and human (THP-1) macrophages were used, and levels of proinflammatory markers iNOS, ROS, as well as activated and inactivated STAT1 protein levels were monitored after treatment with IFN-γ, MMP12-cleaved IFN-γ, or anti-inflammatory IL-4. This analysis showed that cleaved IFN-γ leads to reduced iNOS, ROS, and phosphorylated STAT1. To test the functional aspect of these results, the authors performed a phagocytosis assay and found reduced phagocytic activity in cells treated with MMP12-cleaved IFN-γ when compared to IFN-γ treated cells. In vivo studies were next used to discover that the loss of MMP12 leads to increased IFN-γ-dependent proinflammatory markers in lupus and arthritis mouse models. Using Mmp12+/+ and Mmp12-/- mice, the authors went on to confirm that the catalytic activity of MMP12 is necessary for the IFN-γ –dependent cellular switch from proinflammatory to immunosuppressive. Importantly, the authors showed the significance of misregulated IFN-γ by demonstrating that reduced MMP12 mRNA levels are correlated with systemic lupus erythematosus clinical deterioration, and also demonstrated increased active IFN-γ in glomeruli of patients suffering from lupus nephritis when compared to healthy individuals. Altogether, the authors present strong evidence supporting the importance of MMP12 in regulating IFN-γ-dependent inflammatory responses and highlight a potential diagnostic and therapeutic path for the future of autoimmune

disease treatment. There are some minor points that require clarification that are outlined below, but overall, this is a valuable contribution to the current understanding of immunological regulation.

Main concerns:

1. Wording and interpretation question: The authors state that IFN-γ cleavage is reduced. This is stated both in the main title of the manuscript, as well as in the title of the section focused on patients with SLE lupus nephritis data. However, this only seems to be proven in this manuscript in in vitro studies. Specifically, Figure 7 illustrates an increase in active IFN-γ (as the authors correctly state in the main text), rather than reduced levels of the cleaved form. The wording of the titles should be adjusted accordingly. The results anyway provide support for the author's hypothesis; the conclusions just need to be stated correctly.

2. Connected to the point above, the antibody raised against the N-terminus region of IFN-γ should detect all three IFN-γ forms mentioned – the full length and active and the two cleaved and inactive forms. Have the authors tried to resolve full length from the 2nd cut form by western blotting or other biochemical separations using the generated antibodies? This would provide further support for their conclusion. It is understood that the amount of patient tissue samples may not be sufficient for such studies. Have the authors tried to do this in mouse models? Is this what the authors try to show with the arrows in Figure 6e? These are not explained in the text, and the background of the blot seems brightened.

3. Missing loading controls should be added:

a. Loading controls are necessary for Fig4a and c for the 15min samples, as well as for Fig4b and d (loading control under iNOS).

b. As the total protein levels for STAT1 fluctuate, it would help to add an additional standard loading control for fig8 a, b, d and e.

Minor concerns:

1. Fig2a – the authors should clarify why the MMP12 levels go down when IFN-γ is cleaved (lanes 4 and 5).

2. Fig3a needs an x axis label (days)

3. Fig 4f - Since this experiment nicely displays a function related to active vs. inactive IFN-γ, some additional information would be helpful to clarify the measurement of the phagocytic index. The authors mention that they quantified the number of beads per cell. How many cells were used, as how were the microscopy images acquired and processed for the quantification? Does the description of n=3; N=2 refer to 3 biological replicates in technical duplicates, and is this representative of both panels e and f in this figure?

4. Fig7a the right part of the middle panel of the western blot looks washed out. Do the authors have another blot or exposure they can use?

5. Fig 7a – it seems like the antibody against C-terminal-2 would be able to recognize both the full-length and the form cleaved at 1st cut. The authors should reword the description in the main text.

Reviewer #3 (Remarks to the Author):

The manuscript by Dufour et al provides data supporting the authors' hypothesis that MMP12-mediated processing of interferon-gamma (IFNg) is a method for dampening the inflammatory response, and in the absence of such proteolytic processing, a chronic pro-inflammatory response is seen. Moreover, the authors provide evidence that the MMP12/IFNg axis is altered in autoimmune disease such as systemic lupus erythromous (SLE). Overall, this is a well-conducted study with multiple experimental findings that reinforce each other and support the hypothesis. The findings are of interest particularly with respect to elucidating control mechanisms for different phases of inflammation. However, there are some issues with the choice of models, and with some of the descriptions of animals.

The data showing processing of IFNg by MMP12 and the resulting loss of IFNg-signaling and downstream effects in vitro and in the acute inflammatory peritonitis model in vivo are robust and strongly supportive of the central idea. However, an acute peritonitis (or in vitro treatment of "macrophage" cell lines) is not the same as a chronic lymphoid-driven disease such as SLE. It is puzzling that the MMP12+/+ and MMP12-/- MRL/lpr mice appear to be similar with respect to lymph node size at baseline, which here is 90 days old. At this age, MRL/lpr mice generally show a demonstrable lymphoproliferative phenotype, which is the basis for the disease, thus the lack of difference suggests that MMP12-mediated processing of IFNg is not causal. The fact that differences are seen only after administration of adjuvant to induce an inflammatory response, reinforces the idea that the MMP12/IFNg axis is more critical for acute inflammatory events rather than chronic autoimmune disorders, although there may be an important role in disease flares. This should be discussed. Currently in the discussion, there is an implication for mechanistic contribution of IFNg to disease in the MRL/lpr models that the authors acknowledge cannot be extrapolated yet to the human disease. However, even in the mouse models, the authors have not done a rescue experiment of adoptively transferring MMP12+/+ macrophages into the MMP12-/- MRL/lpr mice to show requirement for this axis in disease progression.

In terms of the description of the mouse models, the genetic backgrounds are not very clear. For example are the MMP12-/- mice backcrossed to C57bl/6 background after having been generated with 129SvEV ES cells? Or are they a true 129XC57 mixed background? This has important implications for controls, especially as C57bl/6 mice are considered a "low inflammation" strain. Following on from this, are MRL/lpr MMP12+/+ mice generated in the same way (by backcrossing from a similar starting strain) or are they just MRL/lpr mice? Similar questions apply to the crossing of 129SvEV MMP12-/- onto the B10.RIII mice.

On additional question regarding the alterations in macrophage polarization observed in the presence and absence of MMP12 in vivo: are the total number of macrophages in the tissue similar, as well as the total number of circulating monocytes?

Minor Point:

In the summary/abstract, the term 'macrophage-specific' is used to refer to MMP12. Since other cell types can express this protease, the term 'macrophage-associated' is more correct.

REVIEWER 1

Interferon-gamma (IFNg) is a central mediator in regulating the activation status of macrophages and their contribution to inflammation. In this paper, Dufour et al describe a novel and potentially interesting mechanism that pertains to the potential of MMP12 to cleave IFNg, and thus curb inflammatory macrophage activity. Further studies then relate to how disruption of this mechanistic pathway may contribute to autoimmune diseases, such as systemic lupus erythematus (SLE; lupus) and rheumatoid arthritis, both in humans and mouse models.

The study is detailed, and the paper is well written. The authors produced a significant body of work which is generally supportive of their hypothesis. Nevertheless, addressing the points below is highly recommended to significantly improve the robustness of the results and the impact of the work.

Response: It was gratifying that all 3 Reviewers considered our paper to be of great interest with a novel mechanism pertinent to the regulated transition of immune responses in acute inflammation and autoimmune disease. In addressing the reviewers' points and adding much new human and mouse data, the paper is further strengthened. We trust it can now move forward so other groups and clinicians can evaluate, consider and build on our hypothesis.

Major comments:

1. Cohorts

a. Major conclusions made by Dufour et al pertain to the relevance of the described mechanism to SLE, and that MMP12 protects from the development of active disease (page 4). If so, demonstrating that untreated active lupus is associated with decreased MMP12 in one cohort, and that treated SLE patients have comparable MMP12 levels to healthy controls in a totally separate cohort, is not sufficiently convincing. A longitudinal cohort including the same patients studied over time (e.g. active vs. treated) is required, and should not be particularly challenging to obtain. There are many lupus cohorts available to study, and if not lupus patients are not rare in rheumatology units. This would also allow more complete characterization of the cohorts from the point of view of important clinical and epidemiological features (age, sex, duration of disease, disease manifestations, etc). Moreover, this approach could potentially strengthen a causation argument, showing that MMP12 normalization occurs early after instituting treatment and precedes clinical improvement, while patients whose MMP12 levels are not affected do not improve.

Done as suggested: i) To address this we searched 3 of the major databases (on 19/07/2017) (GEO (26,622 human array or sequencing-based RNA data sets), ArrayExpress (70,672 data sets of 2,242,940 assays), and Gemma (4,118 data sets)) for data series relating to human lupus transcript analyses. We individually examined more than 239 datasets. Although there are 152 data series for human lupus in GEO and 130 in Array Express, surprisingly there are *only 5 longitudinal data series* in these databases for lupus, 3 of which had no baseline patient data prior to treatment and so were not suited and only 2/5 had untreated patient data as below:

i) GSE65391 *(Banchereau R et al. 2016. Personalized Immunomonitoring Uncovers Molecular Networks that Stratify Lupus Patients. Cell (165):551-65)* did not detect MMP12 in whole blood (see figure), and in fact, previous studies showed that *Mmp12* is not expressed or detectable in whole blood.

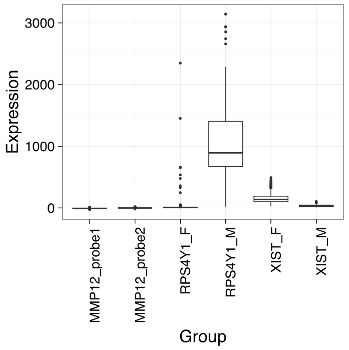

ii) ***New* Supplementary Fig. 3** presents a longitudinal comparison of *Mmp12* and *Ifng* expression in 20 SLE patients with their SLEDAI multivariate disease-activity score over time (31 time points)). Patient information *e.g.* ethnicity, gender, age, SLEDAI, drug treatment *etc.* is in Tables S4-S5 from GSE11909 (Ref 28). **Figure 1e** summarizes this association of longitudinal time dependant changes of *Mmp12* and *Ifng* with SLEDAI. NB, this microarray analysis was longitudinal data of only *untreated* patients.

Legend: RPS4Y1 in female (F) was used as a negative control as it is not expressed in female, but is expressed in male (M) and was then used as a positive control. XIST is only expressed in females, but not in males, and was used as an additional control.

Thus, other than GSE11909, there are no published data sets with untreated and then treated SLE patients or where *Mmp12* is expressed that we can analyse as requested by the Reviewer.

As the Review appreciates, the suggested in depth clinical study proposed is a major undertaking. Launching a collaboration with new clinical teams (we are not clinicians) and recruiting large numbers of patients for longitudinal analysis pre- and post- treatment is beyond a reasonable time frame for revising this paper. As well, Reviewer 3 considered that the principal mechanistic role for the cleavage of IFN-γ lies more towards regulating acute flares of inflammation. With our present and new data, we agree. Therefore, we have realigned our conclusions and discussion along these lines, and qualified our conclusions regarding your point at the end of discussion (page 14).

b. The number of mice studied for many experiments (as subsets of larger cohorts), as well as the number of lupus biopsies stained, is not sufficient. It is difficult to make any solid conclusions from n=3 in each group, as was sometimes used.

Done as suggested. We added more human patients up to the limit approved by the University of BC and Vancouver General Hospital Human Ethics Boards. The *new* **Figure 7b-c** and **Supplementary Figures 11-12** presents the new analyses for a total of 10 patients. The new data are consistent with that previously presented and strengthened the p values.

We also made the *n* values clear for the 206 human patients in **Figure 1**:
a-b: n = 114: SLE patients (n = 102) or healthy control subjects (n = 12).
c-d: n = 72: SLE patients (n = 40) or healthy control subjects (n = 32).
e: n = 20.

Our statistician, Ass. Professor Gabriela Cohen-Freue, Department of Statistics, UBC, considered the studies to be adequately powered for statistical significance and conclusions. However, to address this point *new* mouse data has been added increasing *n* values *e.g.* **Fig. 3a,b**, **Supplementary Figs. 9,10**. All *n* and *N* values are now presented for a total of **519 mice**.

We can now see that in some instances, our descriptions of the longitudinal experiments were not clear, perhaps leading the reviewer having an impression that the number of animals at the end points were the total animal numbers. The animal numbers are now fully detailed in the legends and Methods. The only n = 3 refers to the number of mice used for histological analysis at the last time point per group (from n = 18 and 20 mice Fig. 5a-d; from 30 and 28 mice Fig. 5e-g; from n = 16 and 12 Fig. 6a-d). All other longitudinal studies can now be seen to be powered adequately e.g.:

Fig. 3 Acute peritonitis: Numbers have been increased with new analyses as suggested:
n = 20 for WT, n = 20 for KO mice; repeated N = 2 (n = 80 total).
Fig. 5a B10.RIII arthritis: n = 18 and n = 20 for WT and KO, respectively.
Fig. 5e MRL/*lpr* arthritis: n = 30 and n = 28 for WT and KO, respectively.
Fig. 6 MRL/*lpr* lymphadenopathy: n = 16 & n = 12 for WT and KO, respectively; repeated N = 2.
Fig. 6e lymphadenopathy: n = 16 mice, 8 for WT and 8 for KO. *N.B.* only 4 / 16 are on this gel.
Fig. 6f,g MRL/*lpr* glomerulonephritis: n = 11 and n = 15 for WT and KO, respectively.
Fig. 8 peritonitis time courses: Numbers have been increased with new analyses: n = 5, n = 4 for each of the 8 time points for WT & KO, respectively; and n = 4, n = 4 and n = 4 for each of the WT mice treated *in vivo* with drug or vehicle and the KO mice.

Supplementary Fig. 9: n = 152 mice:
9a, 26 additional mice to those in **Fig. 6** ($Mmp12^{+/+}$ (n = 13) and $Mmp12^{-/-}$ (n = 13))
9b,c, 16 additional mice to those in **Fig. 6** ($Mmp12^{+/+}$ (n = 22) and $Mmp12^{-/-}$ (n = 8))
9d, 35 additional mice to those in **Fig. 6** ($Mmp12^{+/+}$ (n = 18) and $Mmp12^{-/-}$ (n = 17))
9e, 26 mice ($Mmp12^{+/+}$ (n = 11), $Mmp12^{-/-}$ (n = 15))
9f, 49 mice ($Mmp12^{+/+}$ (n = 22), $Mmp12^{-/-}$ (n = 27))
Supplementary Figure 10: n = 48 mice:

10a, 42 mice (*Mmp12*$^{+/+}$ (*n* = 15), *Mmp12*$^{-/-}$ (*n* = 27))
10b, 6 mice (*Mmp12*$^{+/+}$ (*n* = 3), *Mmp12*$^{-/-}$ (*n* = 3))

2. Specificity: It is not clear from the design of the study whether this mechanism is specific for lupus (in human disease) or inflammatory arthritis (collagen or CFA induced in mice), or is more broadly applicable. First, since the animal models involve arthritis, analyzing MMP12 levels in a cohort of patients with rheumatoid arthritis is indicated together with the lupus cohort analysis. Furthermore, besides the comparison to normal individuals, including a cohort of patients with chronic non-inflammatory disease (e.g. diabetes, osteoarthritis) is important. Staining kidneys of patients with non-lupus, inflammatory and non-inflammatory renal disease in Figure 7 is important as well.
To clarify, showing for example that patients with untreated rheumatoid arthritis also have significantly reduced MMP12 expression would not decrease from the significance of paper and the findings in lupus. It is, however, important to determine whether the proposed mechanism is limited to a single autoimmune disease or phenotype.
Response: The extreme severity of the *Mmp12* KO lymphadenopathy severely limits our ability to study the more slowly developing spontaneous manifestations of arthritis and lupus nephritis before sacrifice at day 100 is required by the UBC veterinarians mandated by the Ethics Board (*see* below for response to CFA enquiry). We also agree with an excellent point raised by Reviewer 3, who considered that the principal mechanistic role for the cleavage of IFN-γ lies more towards regulating acute inflammation and acute flares. Therefore, we have realigned our conclusions and discussion to support this rendering some of the suggested extensive clinical studies proposed peripheral to the scope of our study, which was to firmly establish a new post-translational mechanism of IFN-γ regulation biochemically, proteomically, *in vitro*, in animal models, with human relevance indicated by patient examples. As the Review appreciates, the suggested in depth clinical study proposed is a major undertaking beyond the capability of our lab without engaged clinical partner teams and large numbers of patients recruited. Thus, we trust our paper will encourage other investigators with the capacity to perform clinical studies to establish the mechanism in various diseases.

We do take the reviewer's point seriously. In **Figure 7** (human lupus nephritis) we added 5 controls of *non*-SLE kidney biopsies from patients with non-inflammatory renal diseases having mild to moderate arteriosclerosis with healthy, non-lupus, non-inflammatory disease, *see new* Methods page 26. Their data shows that in non-lupus, non-inflammatory disease there were high levels of MMP12 and very low levels of IFN-γ and low levels of cleaved IFN-γ. This was the maximum number of healthy biopsies allowed by the UBC/VGH Human Ethics Boards for this study. We also increased the number of SLE nephritis biopsies to the maximum allowed by these boards (*n* = 5).

We performed Western blot analyses of synovial fluid taken from 6 untreated acute rheumatoid arthritis patients using a variety of antibodies. Full length IFN-γ (anti-N and C-term 1) was associated with low to undetectable MMP12 in acute rheumatoid arthritis synovial fluid. This was coincident with IFN-γ markers MHC class II, S100A9 and STAT1 whereas STAT6 was detected at low levels. *See* first figure to right:

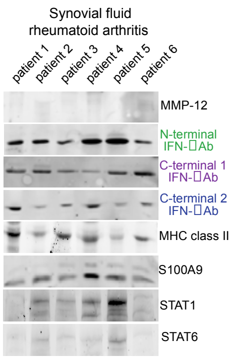

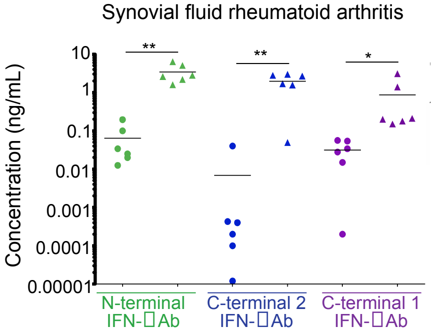

Next, we compared plasma from the same 6 rheumatoid arthritis patients (triangles) with plasma from 6 healthy individuals (circles). The rheumatoid patients had higher levels of full-length IFN-γ than healthy controls with consistent concentrations in the low ng/mL. We agree these promising results could be bolstered by further studies recruiting a larger cohort of patients. Again, for a

biochemical lab, there is a difficulty in obtaining synovial fluid from healthy age matched patients and so this requires considerable time to obtain ethics approval, recruit clinicians, patients and collect synovial fluid for analysis.

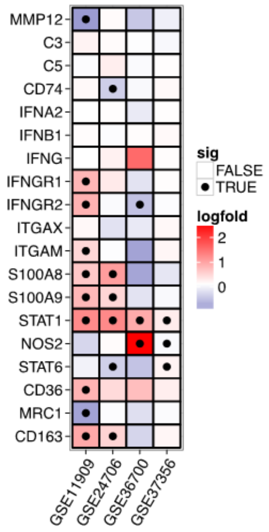

Finally, in PBMCs of SLE patients (*n* = 299) vs. controls (*n* = 451) we found low levels of MMP12 mRNA (blue/white) associated with elevated levels of IFN-γ and IFN-γ response genes in 4 human gene array data sets of SLE:

1. GSE11909 data set <u>from SLE patients</u>: Chaussabel D et al. 2008. A Modular Analysis Framework for Blood Genomics Studies: Application to Systemic Lupus Erythematosus. Immunity 29;150-164
2. GSE24706 data set <u>from SLE patients</u>: Li QZ et al. 2011. Risk factors for ANA positivity in healthy persons. Arthritis Research & Therapy 13;150-164
3. GSE36700 data set <u>from rheumatoid arthritis, osteoarthritis and SLE patients</u>: Toukap AN et al. 2007. Identification of Distinct Gene Expression Profiles in the Synovium of Patients with Systemic Lupus Erythematosus. Arthritis & Rheumatism 56(5);1579-88
4. GSE37356 data set <u>from SLE patients</u>: Korman BD et al. 2014. Inflammatory expression profiles in monocyte-to-macrophage differentiation in patients with systemic lupus erythemastosus and relationship with atherosclerosis. Arthritis & Rheumatism 56(5);1579-88

Thus, our strong *in vitro*, proteomic, biochemical, cell and animal model data suggest a more generally applicable mechanism potentially not limited to SLE or lupus nephritis, but also at play in several IFN-γ-linked phenotypes. This will require further clinical analyses to firmly establish. In light of the comment we have tempered the conclusion in introduction, results and discussion to convey this (page 3, 14).

3. MRL/lpr experiments: One major advantage of the MRL-lpr/lpr strain which makes it highly suitable for modeling human disease is the spontaneous onset of the autoimmune and clinical phenotype. Indeed, the large majority of the lupus relevant studies in this strain involve unmanipulated mice. Moreover, spontaneous arthritis is a feature of MRL-lpr/lpr mouse. It is therefore not clear why the authors chose a CFA-accelerated model in wild type and MMP12 KO mice, and furthermore what are the relevance of any results obtained to spontaneous lupus. Moreover, even in the CFA-triggered experimental setup, central features of this model were not studied or provided (proteinuria, circulating anti-nuclear/anti-DNA autoantibodies). It is recommended that the authors study the spontaneous development of disease in the MMP12 wild type and sufficient MRL/lpr mice, including better characterization of renal disease, circulating antibodies and autoantibodies, spleen/lymph node weight, and survival.

Done as suggested:
i) ***new* Supplementary Figure 9a,b,c,e** shows data on spontaneous development of lymphadenopathy in mice before treatment with CFA.
ii) ***new* Supplementary Figure 9f** shows no significant differences were identified in spleen weights for MRL/*lpr* female mice not treated with CFA *vs.* mice injected with CFA.
iii) ***new* Supplementary Fig. 10a** presents mouse whole blood leukocyte and monocyte counts as described on page 26: "*…The whole blood of each mouse (Mmp12$^{+/+}$ (n = 15) and Mmp12$^{-/-}$ (n = 27)) was analysed in technical duplicates*".
iv) ***new* Figure 6f** compares kidney weights in mice not treated with CFA *vs.* treated with CFA to test the *in vivo* effect of MMP12 processing of IFNγ during a CFA proinflammatory disease flare.
v) ***new* Supplementary Figure 9c-e** shows no difference in survival between the untreated and CFA-treated *Mmp12*$^{+/+}$ mice, which only impacts CFA treated female mice and not males.
vi) ***new* Figure 6i**, quantifies both the glomeruli and interstitium together (*n* = 20 per group) in the kidney nephritis model of SLE (consistent with results for glomeruli quantification alone, **Figure 6h**). Further descriptive information of how renal disease is included on page 26.
*"**Mouse glomerulonephritis grade scoring.** Glomerulonephritis grade in mouse kidneys was scored as described[60-62]. In brief, the kidneys were scored according to the International Society of Nephrology/Renal Pathology Society classification of lupus nephritis scoring system as follows: Grade I, minimal mesangial lupus nephritis; Grade II, mesangial proliferative lupus nephritis; Grade III, focal lupus nephritis; Grade IV, diffuse segmental (IV-S) or global (IV-G) lupus nephritis; Grade*

Class V, membranous lupus nephritis; and Grade Class VI, advanced sclerosing lupus nephritis. The means of the grade scores from Mmp12$^{+/+}$ MRL/lpr (n = 11) and Mmp12$^{-/-}$ MRL/lpr (n =15) mice were calculated by analysis of a minimum of 10 fields with a minimum of 5 glomeruli per field for each mouse kidney biopsy. For immunostaining analyses of mouse kidney glomeruli, and glomeruli plus the interstitium a minimum of 10 different fields, with each field having a minimum of 5 glomeruli were examined and quantified for each antibody, for each of these two separate analyses (N = 2). Three mice per group (Mmp12$^{+/+}$ (n = 3) and Mmp12$^{-/-}$ MRL/lpr (n = 3)) were analyzed separately for the glomeruli and the glomeruli and interstitium staining quantifications."

vii) ***new* Supplementary Figure 11**, the lupus nephritis SLE class, creatinine, glomerular filtration rate (GFR), the albumin/creatinine ratio (ACR), the 24 h proteinuria, the erythrocyte sedimentation rate (ESR), the C-reactive protein (CRP), the C3, the C4 and the dsDNA at biopsy as well as additional biopsy details for each individual human patient are provided. As there could be differences between comparing mouse and human, we decided to focus on the clinical features of human patients.

Response re spontaneous vs. CFA induction of disease.

The Jackson Laboratory used to supply the 006825-MRL/MpJ-Fas^{lpr}/2J substrain, which developed systemic autoimmunity, arthritis and immune complex glomerulonephritis slowly. In our hands, these mice survived more than 120-150 days before their lymphadenopathy reached humane end point requiring euthanasia. We used this strain in former studies with other protease knockout backcrosses and we believe that the Reviewer may have experience with this strain. However, The Jackson Laboratory since reported that the 006825 substrain had an apparent loss of the lymphoproliferative phenotype (albeit with no detectable genetic change from the parental strain 000485-MRL/MpJ-Fas^{lpr}/J), and replaced 006825 with a new 000485-MRL/MpJ-Fas^{lpr}/J substrain derived from the parental 000485.

Due to a refreshing of this substrain of MRL/*lpr* by the Jackson Lab, these mice develop severe lymphadenopathy earlier than the previously supplied substrain. While arthritis develops spontaneously in 100% of our backcrossed *Mmp12*$^{+/+}$ and *Mmp12*$^{-/-}$ mice the time of onset is variable and most animals reach humane endpoint before the development of lupus, requiring euthanasia by around day 110 in the *Mmp12*$^{+/+}$ mice and at around day 100 for *Mmp12*$^{-/-}$ mice, which limits experimental studies considerably. Thus, spontaneous arthritis and immune complex glomerulonephritis/SLE does not have time in our backcrosses to develop sufficiently in the time frame before sacrifice is mandated. Initiation of disease with CFA allows us to stay within a testable window: One advantage of CFA injection is the synchronization of the initiation of disease development, facilitating analysis: A single injection of CFA is in fact common as it stimulates the hyperproduction of IFN-γ and expression of MHC class II genes (Billiau 01 Ref 40; Balomenos 98 Ref 39; Brand 07 Ref 38), which is required for lymphoaccumulation, lupus development, and a Th1/classical activation of macrophages. *On page 25* we now state: *"To synchronize the initiation of systemic autoimmunity and to develop sufficient disease before human endpoints require euthanasia, we injected... CFA synchronizes the time of disease onset ensuring reasonably severe and consistent disease stage between animals before excessive lymphadenopathy necessitates animal euthanasia"*. Synchronization also facilitates the quantitative histomorphometric comparison of similarly affected joints in each mouse group (+/- MMP12) at different times. The reviewers point is well taken and so we have dampened our conclusions in the text at several places to indicate that the effects of MMP12 may be more apparent on acute disease and flare ups of chronic disease.

On page 8-9 we now state: "*The signature lymphoproliferative response in female MRL/lpr mice, as evidenced by enlarged lymph nodes, was seen by day 90 in female Mmp12$^{-/-}$ (n = 18) and Mmp12$^{+/+}$ (n = 38) mice, (Fig. 6a, Supplementary Fig. 9a, b, N = 2) and later for males (n = 13 each for Mmp12$^{-/-}$ and Mmp12+/+ male mice, Supplementary Fig. 9a). Consistent with the MMP12-dependent*

differences in lymph node size apparent by day 90 in the female mice and that lead to animal euthanized before day 100 according to UBC Animal Ethics Guidelines, CFA synchronization further expanded the differential increase in lymphadenopathy manifest in the Mmp12$^{-/-}$ MRL/lpr females (n = 18, N = 2) over that seen for the female Mmp12+/+ MRL/lpr mice (n = 38, N = 2) (Fig. 6a, Supplementary Fig. 9b), supporting the role for MMP12 in acute inflammatory responses."

Minor comments:

a. What is the physiological function of MMP12? It is mentioned that it has "established mechanisms in regulation of macrophage activation and to other anti-inflammatory activities", but what are these? Could any of the results reported be due to other effects of MMP12, besides cleavage of IFNg?
b. What is the phenotype of MMP12 KO mice? Furthermore, is there a difference in IFNg related genes at baseline? (Figure 3A).

Done as suggested: *New* Figure 3a: There was no IFNγ at day 0 in either *Mmp12$^{+/+}$* or *Mmp12$^{-/-}$* mouse peritoneal fluid. We elaborated on the function of MMP12 on page 3 (and elsewhere): "*MMP12 is long known to cleave substrates with importance in macrophage migration[21], e.g. elastin, fibronectin, laminin, entactin, type I and IV collagens, and proteoglycan core proteins[21-23]. Mmp12$^{-/-}$ mice are relatively healthy animals, and consistent with the extracellular matrix substrates of MMP12, exhibit a defect in elastinolytic activity and a reduction in macrophage invasive ability to penetrate basement membranes, and a decrease in IL-13[21,24]. MMP12 also exhibits antimicrobial activity[25] and by cytokine cleavage plays roles in antiviral immunity by cleavage of IFN-α, but <u>not</u> IFN-β[6], and reduces neutrophil influx via chemokine processing[22,26]*".

c. Is it possible that other IFNs are also being targeted by MMP12? e.g., besides showing that MMP12 doesn't cleave IL-4 (Supp. Figure 5), what is the effect on type I interferon?

Done as suggested: We demonstrated that MMP12 cleaves IFN-α, but <u>not</u> IFN-β during viral infection in *"Marchant D,… **Dufour A**,… and **Overall CM**. 2014 **Nature Medicine**; 20(5):493-502",* previously cited, but now made more obvious: twice in Introduction (page 2 and 3) *e.g.* "*…a role in antiviral immunity by cleavage of IFN-α, but not IFN-β[6],...*" and "*Potentially contributing to the temporal regulation of IFN-γ activity and hence immune cell responses, is the post-translational modification of IFN-γ as recently shown for IFN-α[6], although this is not known*"; and Discussion, page 13.

d. In some of the in vitro studies, it would be interesting to include an additional control such as LPS which stimulates M1 type macrophages via STAT1, but is independent of IFNg. It is then possible to demonstrate the specificity of MMP12 affecting macrophages via IFNg related pathways.

Answer: Numerous publications (*e.g.* ten are listed below) show that IFN-γ is an important mediator of LPS responses. As LPS and IFN-γ work in synergy and not independently, such a further control is unlikely to yield a meaningful outcome that would add significantly to our data.

1. Blanchard DK et al. Interferon-gamma induction by lipopolysaccharide: dependence on interleukin 2 and macrophages. 1986 Journal of Immunology; 136(3):963-970.
2. Kanevskiy LM et al. LPS induces IFN-γ production in human NK cells. 2013 Frontiers in Immunology; 4(11):1-10.
3. Varma TK et al. Endotoxin-induced gamma interferon production: contributing cell types and key regulatory factors. 2002 Clinical and Vaccine Immunology; 9(3):530-43.
4. Southworth T et al. IFN-γ synergistically enhances LPS signaling in alveolar macrophages from COPD patients and controls by corticosteroid-resistant STAT1 activation. 2012 British Journal of Pharmacology; 166:2070-83.
5. Yoshino S et al. Bacterial LPS acts as an adjuvant to induce autoimmune arthritis in mice. 2000 Immunology; 99:607-14
6. Fultz MJ et al. Induction of IFN-gamma in macrophages by LPS. 1993 International Immunology; 5(11):1383-92.
7. Yoshino S and Ohsawa M The role of LPS injected systemically in the reactivation of collagen-induced arthritis in mice. 2000 British Journal of Pharmacology; 129:1309-14.
8. Held TK et al. Gamma Interferon Augments Macrophage Activation by LPS by Two Distinct Mechanisms, at the Signal Transduction Level and via an Autocrine Mechanism Involving Tumor Necrosis Factor Alpha and Interleukin-1. 1999. Infection and Immunity; 67(1):206-12.
9. Lee JY and Sullivan KE Gamma Interferon and LPS Interact at the Level of Transcription To Induce Tumor Necrosis Factor Alpha Expression. 2001 Infection and Immunity; 69(5):2847-2852
10. Negishi M et al. Lipopolysaccharide (LPS)-induced Interferon (IFN)-gamma Production by Decidual Mononuclear Cells (DMNC) is Interleukin-2 and IL-12 Dependent. 2011 American Journal Reproductive Immunology; 65:20-27.

e. Is it standard practice to demonstrate NETs with H&E staining?

Clarified as suggested: Yes, we have added to Experimental, page 25: "*NETs were previously*

characterized in collagen-induced arthritis by a variety of stains including hematoxylin and eosin in combination[22] or alone[58,59], the histological appearance and distribution of which are consistent with the NETs identified here by hematoxylin and eosin."

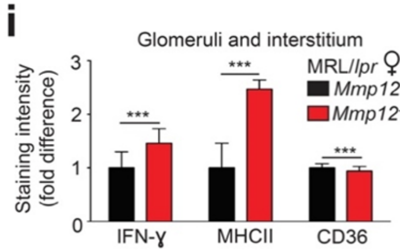

f. The conclusion reached in the figure legend of Supplemental figure 8 about auto-reactive B cells is not justified without at least looking at circulating levels of autoantibodies.

Done as suggested: Fair point and statement removed (now Supplementary Fig. 9f).

g. Additional detail on the scoring of the kidney histology sections would be beneficial. How many glomeruli were examined? What parameters were scored?

Done as suggested: Very good point and apologies for the oversight. For the mouse studies, see *new* ***Methods*** *(page 25) and* ***Figure 6g*** *y axis label changed. "****Mouse glomerulonephritis grade scoring.*** *Glomerulonephritis grade in mouse kidneys was scored as described[60-62]. In brief, the kidneys were scored according to the International Society of Nephrology/Renal Pathology Society classification of lupus nephritis scoring system as follows: Grade I, minimal mesangial lupus nephritis; Grade II, mesangial proliferative lupus nephritis; Grade III, focal lupus nephritis; Grade IV, diffuse segmental (IV-S) or global (IV-G) lupus nephritis; Grade Class V, membranous lupus nephritis; and Grade Class VI, advanced sclerosing lupus nephritis. The means of the grade scores from Mmp12$^{+/+}$ MRL/lpr (n = 11) and Mmp12$^{-/-}$ MRL/lpr (n =15) mice were calculated by analysis of a minimum of 10 fields with a minimum of 5 glomeruli per field for each mouse kidney biopsy.*
For immunostaining analyses of mouse kidney glomeruli, and glomeruli plus the interstitium a minimum of 10 different fields, with each field having a minimum of 5 glomeruli were examined and quantified for each antibody, for each of these two separate analyses (N = 2). Three mice per group (Mmp12$^{+/+}$ (n = 3) and Mmp12$^{-/-}$ MRL/lpr (n = 3)) were analyzed separately for the glomeruli and the glomeruli and interstitium staining quantifications."

For the human analyses, see *new* **Methods** (page 26) describing grading and reflecting the increased numbers of healthy and SLE patient biopsies included as new data: "***Human lupus nephritis analyses and grade scores.*** *Five kidney biopsies from non-SLE patients with mild to moderate arteriosclerosis and healthy, non-lupus, non-inflammatory kidney disease, and kidney samples from SLE patients were classified independently by one blinded pathologist and one nephrologist: Class I, minimal mesangial lupus nephritis; Class II, mesangial proliferative lupus nephritis; Class III, focal lupus nephritis; Class IV, diffuse segmental (IV-S) or global (IV-G) lupus nephritis; Class V, membranous lupus nephritis; Class VI, advanced sclerosing lupus nephritis. Healthy (n = 5) and active (A) (n = 5) SLE Grade III-(A), IV-Global-(A), and IV-Segmental-(A) kidney biopsies were then selected for immunochemical analysis. Glomeruli were examined and quantified for each antibody from all human biopsies of the healthy kidneys (n = 43 glomeruli) and SLE patient kidneys (n = 46 glomeruli) and scored according to these classes: Class I, normal glomeruli by light microscopy; Class II, mesangial hypercellularity or mesangial matrix expansion by light microscopy with immune deposits; Class III, active or inactive focal, segmental or global endocapillary or extracapillary glomerulonephritis involving less than 50% of all glomeruli; Class IV, active or inactive focal, segmental or global endocapillary or extracapillary glomerulonephritis involving more than 50% of all glomeruli; Class V, global or segmental subepithelial immune deposits or their morphologic sequelae by light microscopy; and Class VI, more than 90% of glomeruli sclerosed.*

Moreover, both H+E and IHC (figure 6i) demonstrate peri-glomerular/interstitital disease as well. Tubulo-interstitial disease should be quantitated separately from glomerular disease, whether or not both were included in the glomerulonephritis score provided in figure 6g.

Done as suggested: *new* **Fig. 6i**.

REVIEWER 2

This manuscript elegantly demonstrates the importance of IFN-γ regulation during cellular inflammatory response. The authors found an inbuilt negative feedback loop regulating IFN-γ activity that correlates with a decrease in proinflammatory markers. Specifically, they found that macrophage-specific matrix metalloproteinase 12 (MMP12) cleaves IFN-γ, which renders the ligand inactive, highlighting the importance of post-translational cleavage events in the regulation of immune responses. To identify the cleavage sites, the authors used a top-down mass spectrometry approach and identified the C-terminal truncations to occur at 157Met↓Leu158 and 135Glu↓Leu136. To characterize the role of truncated IFN-γ , mouse (RAW264.7) and human (THP-1) macrophages were used, and levels of proinflammatory markers iNOS, ROS, as well as activated and inactivated STAT1 protein levels were monitored after treatment with IFN-γ, MMP12-cleaved IFN-γ, or anti-inflammatory IL-4. This analysis showed that cleaved IFN-γ leads to reduced iNOS, ROS, and phosphorylated STAT1. To test the functional aspect of these results, the authors performed a phagocytosis assay and found reduced phagocytic activity in cells treated with MMP12-cleaved IFN-γ when compared to IFN-γ treated cells. In vivo studies were next used to discover that the loss of MMP12 leads to increased IFN-γ-dependent proinflammatory markers in lupus and arthritis mouse models. Using Mmp12+/+ and Mmp12-/- mice, the authors went on to confirm that the catalytic activity of MMP12 is necessary for the IFN-γ –dependent cellular switch from proinflammatory to immunosuppressive. Importantly, the authors showed the significance of misregulated IFN-γ by demonstrating that reduced MMP12 mRNA levels are correlated with systemic lupus erythematosus clinical deterioration, and also demonstrated increased active IFN-γ in glomeruli of patients suffering from lupus nephritis when compared to healthy individuals.

Altogether, the authors present strong evidence supporting the importance of MMP12 in regulating IFN-γ-dependent inflammatory responses and highlight a potential diagnostic and therapeutic path for the future of autoimmune disease treatment. There are some minor points that require clarification that are outlined below, but overall, this is a valuable contribution to the current understanding of immunological regulation.

Main concerns:

1. Wording and interpretation question: The authors state that IFN-γ cleavage is reduced. This is stated both in the main title of the manuscript, as well as in the title of the section focused on patients with SLE lupus nephritis data. However, this only seems to be proven in this manuscript in in vitro studies. Specifically, Figure 7 illustrates an increase in active IFN-γ (as the authors correctly state in the main text), rather than reduced levels of the cleaved form. The wording of the titles should be adjusted accordingly. The results anyway provide support for the author's hypothesis; the conclusions just need to be stated correctly.

Done as suggested. The new paper title is: "*Post-translational truncation of IFN-γ dampens proinflammatory macrophage responses and is deficient in autoimmune disease*". The new Results subtitle reads: "*Relative levels of MMP12 and C-terminal cleavage of IFN-γ are reduced in lupus nephritis"*. Please note, we added new immunohistological data of biopsies from 2 additional healthy and 2 additional SLE patients, which further strengthened our results.

2. Connected to the point above, the antibody raised against the N-terminus region of IFN-γ should detect all three IFN-γ forms mentioned – the full length and active and the two cleaved and inactive forms. Have the authors tried to resolve full length from the 2nd cut form by western blotting or other biochemical separations using the generated antibodies? This would provide further support for their conclusion. It is understood that the amount of patient tissue samples may not be sufficient for such studies. Have the authors tried to do this in mouse models? Is this what the authors try to show with the arrows in Figure 6e? These are not explained in the text, and the background of the blot seems brightened.

Done as suggested. Good suggestion. We now include new time course data extended to 1,080 min from 120 min (**Fig. 7a**) that shows the N-terminal antibody recognises a lower molecular weight form of IFN-γ during incubation that in fact reaches ~50% of the amounts of intact IFN-γ and so further validates C-terminal processing. However, we never saw all three forms of IFN-y in the same lane or individually resolved over time by the N-terminal antibody alone. This excellent suggestion prompted us to improve the experimental description (page 10).

As we now state on page 10: "As the human and murine IFN-γ sequences diverge significantly in the C-terminus (Supplementary Fig. 4), the anti-human IFN-γ antibodies did not

cross react with mouse IFN-γ”. However, we have added new lupus nephritis patient samples (*n* = 5 healthy and *n* = 5 lupus nephritis) to our study in ***new*** **Figure 7b-c** and **Supplementary Figures 11-12**. In addition, in **Figure 5c,d, 5g,h, 6c,d,** and **6h,i,j** we demonstrated an increase in IFN-γ levels and M1 signature in 2 distinct animal strains and different disease models. In **Figure 6e**, we also found cleaved IFN-γ *in vivo* (2/8 different *Mmp12*$^{+/+}$ mice shown), but not in *Mmp12*$^{-/-}$ mice (*n* = 8), further supporting the link between MMP12 and IFN-γ processing.

The image reproduction of the blot was suboptimal and this has been replaced. The text on page 9 and the legend for **Figure 6e** (page 14) now states that red arrows indicate likely cleaved lower molecular forms of IFN-γ in duplicate *Mmp12*$^{+/+}$ mice.

3. Missing loading controls should be added:
a. Loading controls are necessary for Fig4a and c for the 15min samples, as well as for Fig4b and d (loading control under iNOS).
b. As the total protein levels for STAT1 fluctuate, it would help to add an additional standard loading control for fig8 a, b, d and e.

Done as suggested: Tubulin loading controls for all blots are now shown in **Figure 4a-d** and in **Figure 8a, b, d, e** (actin) and with tubulin in **Fig. 8f** rather than just selected blots.

Minor comments:

1. Fig2a – the authors should clarify why the MMP12 levels go down when IFN-γ is cleaved (lanes 4 and 5)

Done as suggested: During incubation, MMPs are subject to autolysis, probably to turn off activity *in vivo*. What is commonly seen upon addition of MMP inhibitors is stabilization of MMP protein levels due to blockage of activity including autoproteolysis (now in legend for **Figure 2a**).

2. Fig3a needs an x axis label (days)

Done as suggested. Note, we now include new mice and quantification of IFN-γ at day 0.

3. Fig 4f - Since this experiment nicely displays a function related to active vs. inactive IFN-γ, some additional information would be helpful to clarify the measurement of the phagocytic index. The authors mention that they quantified the number of beads per cell. How many cells were used, as how were the microscopy images acquired and processed for the quantification? Does the description of n=3; N=2 refer to 3 biological replicates in technical duplicates, and is this representative of both panels e and f in this figure? Does the description of n=3; N=2 refer to 3 biological replicates in technical duplicates, and is this representative of both panels e and f in this figure?

Done as suggested: See new legend for **Figure 4f** and METHODS, page 22-23: “*The phagocytosis index was determined in each experiment (n = 3 repeats per experiment, and the experiment itself performed twice, N = 2) by counting the number of beads per cell in 20 separate fields containing 5 – 30 cells/image for each repeat.*” *n* = number of replicate assays per experiment; *N* = the number of experiments here and elsewhere. This was clarified in METHODS**,** as mentioned above, and in the legend.

4. Fig7a the right part of the middle panel of the western blot looks washed out. Do the authors have another blot or exposure they can use?

Done as suggested: See ***new*** **Figure 7a.** The N-terminal IFN-γ Ab and C-terminal-1 IFN-γ blots have been replaced with a new experiment and over a longer time course (to 1,080 min).

5. Fig 7a – it seems like the antibody against C-terminal-2 would be able to recognize both the full-length and the form cleaved at 1st cut. The authors should reword the description in the main text.

Done as suggested: In theory yes, but the molecular mass difference is only 1,044 Da, which if present, was not resolved on these gels. Alternatively (and more likely), this indicates that the second cleavage event rapidly followed the first. Nonetheless, in replacing **Figure 7a** blots by those from a new experiment, we now show two cleaved forms of IFN-γ recognised by the N-terminal antibody, which further validates C-terminal processing. As suggested, the description was re-worded in **RESULTS** (page 10). Note: *n* values were increased to 5 healthy and 5 SLE.

REVIEWER 3

The manuscript by Dufour et al provides data supporting the authors' hypothesis that MMP12-mediated processing of interferon-gamma (IFNg) is a method for dampening the inflammatory response, and in the absence of such proteolytic processing, a chronic pro-inflammatory response is seen. Moreover, the authors provide evidence that the MMP12/IFNg axis is altered in autoimmune disease such as systemic lupus erythromous (SLE). Overall, this is a well-conducted study with multiple experimental findings that reinforce each other and support the hypothesis. The findings are of interest particularly with respect to elucidating control mechanisms for different phases of inflammation. However, there are some issues with the choice of models, and with some of the descriptions of animals.

Response: It was gratifying that all 3 Reviewers considered our paper to be of great interest with a novel mechanism pertinent to the regulated transition of immune responses in acute inflammation and as a risk factor in autoimmune disease.

The data showing processing of IFNg by MMP12 and the resulting loss of IFNg-signaling and downstream effects in vitro and in the acute inflammatory peritonitis model in vivo are robust and strongly supportive of the central idea. However, an acute peritonitis (or in vitro treatment of "macrophage" cell lines) is not the same as a chronic lymphoid-driven disease such as SLE. It is puzzling that the MMP12+/+ and MMP12-/- MRL/lpr mice appear to be similar with respect to lymph node size at baseline, which here is 90 days old. At this age, MRL/lpr mice generally show a demonstrable lymphoproliferative phenotype, which is the basis for the disease, thus the lack of difference suggests that MMP12-mediated processing of IFNg is not causal. The fact that differences are seen only after administration of adjuvant to induce an inflammatory response, reinforces the idea that the MMP12/IFNg axis is more critical for acute inflammatory events rather than chronic autoimmune disorders, although there may be an important role in disease flares. This should be discussed. Currently in the discussion, there is an implication for mechanistic contribution of IFNg to disease in the MRL/lpr models that the authors acknowledge cannot be extrapolated yet to the human disease. However, even in the mouse models, the authors have not done a rescue experiment of adoptively transferring MMP12+/+ macrophages into the MMP12-/- MRL/lpr mice to show requirement for this axis in disease progression

Done as suggested. ***New*** **Supplementary Figure 9a:** shows that the lymph node size differences were apparent at day 90 and beyond. The previous figure somewhat masked this important difference due to curve overlays, and perhaps also the text was not clear. As can be seen below and elsewhere *e.g.* pages 7, 8, 12 – 14, conclusions were recast to focus more on the role of the new IFN-γ-MMP12 axis for acute inflammation and chronic disease flares and dampened the implication that IFN-γ cleavage defects lead to chronic inflammation. On page 8 we clarify this: "*The signature lymphoproliferative response in female MRL/lpr mice, as evidenced by enlarged lymph nodes, was seen by day 90 in female Mmp12$^{–/–}$ (n = 18) and Mmp12$^{+/+}$ (n = 38) mice, (Fig. 6a, Supplementary Fig. 9a, b, N = 2) and later for males (n = 13 each for Mmp12$^{–/–}$ and Mmp12$^{+/+}$ male mice, Supplementary Fig. 9a). Consistent with the MMP12-dependent differences in lymph node size apparent by day 90 in the female mice and that lead to animal euthanized before day 100 according to UBC Animal Ethics Guidelines, CFA synchronization further expanded the differential increase in lymphadenopathy manifest in the Mmp12$^{–/–}$ MRL/lpr females (n = 18, N = 2) over that seen for the female Mmp12$^{+/+}$ MRL/lpr mice (n = 38, N = 2) (Fig. 6a, Supplementary Fig. 9b), supporting the role for MMP12 in acute inflammatory responses*".

New human studies added to the paper: We considered this experiment, but for the reasons below we preferred the complementary experiment in acute peritonitis in which we treated WT mice daily with the MMP12 specific inhibitor (*n* = 4 *Mmp12$^{+/+}$* + Rxp470.1; *n* = 4 *Mmp12$^{+/+}$* + vehicle), which recapitulated the *Mmp12$^{-/-}$* results (*n* = 4 *Mmp12$^{-/-}$*) for every marker examined (**Figure 8f**). The suggested experiment may not be clear cut due to expression of MMP12 by cells other than macrophages, now clearer (*e.g.* pages 3, 11). Thus, the systemic administration of the inhibitor would block all cellular sources of MMP12, not just MMP12 from macrophages.

Moreover, as the role of MMP12 extends beyond processing of just IFN-γ, there are additional roles of MMP12 that will most likely contribute to the dampening of the response (*see*

pages 3,13. We now discuss that MMP12 plays a role in the reducing PMN influx, has antiviral immunity roles by cleavage of IFN-α but not IFN-β[12], and has antimicrobial activity[13].

For these reasons, we have not performed this set of extensive mouse experiments, but decided instead to focus our resources on new human data:
***new* Supplementary Fig. 3:** shows new longitudinal SLEDAI analyses for 20 human genes in 20 patients over 31 time points.
***new* Figure 7b and *new* Supplementary Figures 11 and 12** present human data from new human biopsy material analysed for MMP12 and IFN-γ intact and cleaved forms in lupus nephritis (*n* = 10 subjects). This new human data bolsters the considerable gene expression association data already provided from greater than a hundred SLE patients that associates reduced MMP12 with elevated IFN-γ in PBMCs of untreated SLE patients (**Figure 1a,b**; **Supplementary Fig. 1**) and drug-treated SLE (**Figure 1c-e**; **Supplementary Fig. 2**).

In terms of the description of the mouse models, the genetic backgrounds are not very clear. For example are the MMP12-/- mice backcrossed to C57bl/6 background after having been generated with 129SvEV ES cells? Or are they a true 129XC57 mixed background? This has important implications for controls, especially as C57bl/6 mice are considered a "low inflammation" strain. Following on from this, are MRL/lpr MMP12+/+ mice generated in the same way (by backcrossing from a similar starting strain) or are they just MRL/lpr mice? Similar questions apply to the crossing of 129SvEV MMP12-/- onto the B10.RIII mice.
Done as suggested: As now described on page 20 – 21 the original knockout mice were generated by a targeting vector electroporated into 129X1/SvJ derived RW4 ES cells. Correctly targeted ES cells were injected into C57BL/6J blastocysts. The resulting chimeric animals were crossed to C57BL/6J mice to establish the colony. The colony was then backcrossed to C57BL/6J for ten generations and then homozygous mice bred together for several generations. We obtained these strains, established a colony and then backcrossed these homozygous mice with MRL/*lpr* mice for 8 generations and so the *MRL/lpr MMP12*$^{+/+}$ mice were originally derived from here for **Figures 5e-h**, and **6**, and **Supplementary Figs. 8**, **9** and **10**. The reviewer is right about the C57bl/6 strain being considered a "low inflammation strain" and so we utilized the B10.RIII-H2^{r} *H2-T18*^{b} strain to backcross heterozygote mice to establish wildtype and knock out strains as described page 21 for the acute inflammation experiments in **Figures 3**, **5a-d**, and **8**.

On additional question regarding the alterations in macrophage polarization observed in the presence and absence of MMP12 in vivo: are the total number of macrophages in the tissue similar, as well as the total number of circulating monocytes?
Done as suggested: *new* Supplementary Figure 10b our new data shows that the number of CD68^{+} macrophages was reduced in the absence of *Mmp12* in the lymph node tissues of MRL/*lpr* female mice, which is in accordance with previous reports[11,15] (see page 9).
***new* Supplementary Fig. 9f:** As there was no difference in spleen weights of *Mmp12*$^{+/+}$ (*n* = 11) *vs. Mmp12*$^{-/-}$ (*n* = 12) female mice without CFA induction, or *Mmp12*$^{+/+}$ (*n* = 11) *vs. Mmp12*$^{-/-}$ (*n* = 15) female mice after CFA induction, we did not stain for cells here.
***new* Supplementary Figure 10a** shows there were no significant differences in the number of circulating leukocytes and monocytes in *Mmp12*$^{+/+}$ (*n* = 15) and *Mmp12*$^{-/-}$ (*n* = 27) MRL/*lpr* female mice (page 9).

Minor Point:
In the summary/abstract, the term 'macrophage-specific' is used to refer to MMP12. Since other cell types can express this protease, the term 'macrophage-associated' is more correct.
Done as suggested. In addition to Abstract, this was corrected on page 3, 12 and on page 11 we state: "*While MMP12 is predominantly a macrophage-associated*[21,27] *protease, other cells also express the enzyme*[6]*, which systemic administration of Rxp470.1 will block*".

Reviewers' comments:

Reviewer #1 (Remarks to the Author):

Interferon-gamma (IFNg) is a central mediator in regulating the activation status of macrophages and their contribution to inflammation. In this paper, Dufour et al describe a novel and potentially interesting mechanism that pertains to the potential of MMP12 to cleave IFNg, and thus curb inflammatory macrophage activity. Further studies then relate to how disruption of this mechanistic pathway may contribute to autoimmune diseases, such as systemic lupus erythematus (SLE; lupus) and rheumatoid arthritis, both in humans and mouse models.

The study is detailed, and the paper is well written. The authors produced a significant body of work which is generally supportive of their hypothesis. Nevertheless, addressing the points below is highly recommended to significantly improve the robustness of the results and the impact of the work.

Major comments:

1. Cohorts

a. Major conclusions made by Dufour et al pertain to the relevance of the described mechanism to SLE, and that MMP12 protects from the development of active disease (page 4). If so, demonstrating that untreated active lupus is associated with decreased MMP12 in one cohort, and that treated SLE patients have comparable MMP12 levels to healthy controls in a totally separate cohort, is not sufficiently convincing. A longitudinal cohort including the same patients studied over time (e.g. active vs. treated) is required, and should not be particularly challenging to obtain. There are many lupus cohorts available to study, and if not lupus patients are not rare in rheumatology units. This would also allow more complete characterization of the cohorts from the point of view of important clinical and epidemiological features (age, sex, duration of disease, disease manifestations, etc). Moreover, this approach could potentially strengthen a causation argument, showing that MMP12 normalization occurs early after instituting treatment and precedes clinical improvement, while patients whose MMP12 levels are not affected do not improve.

b. The number of mice studied for many experiments (as subsets of larger cohorts), as well as the number of lupus biopsies stained, is not sufficient. It is difficult to make any solid conclusions from n=3 in each group, as was sometimes used.

2. Specificity: It is not clear from the design of the study whether this mechanism is specific for lupus (in human disease) or inflammatory arthritis (collagen or CFA induced in mice), or is more broadly applicable. First, since the animal models involve arthritis, analyzing MMP12 levels in a cohort of patients with rheumatoid arthritis is indicated together with the lupus cohort analysis. Furthermore, besides the comparison to normal individuals, including a cohort of patients with chronic non-inflammatory disease (e.g. diabetes, osteoarthritis) is important. Staining kidneys of patients with non-lupus, inflammatory and non-inflammatory renal disease in Figure 7 is important as well.

To clarify, showing for example that patients with untreated rheumatoid arthritis also have significantly reduced MMP12 expression would not decrease from the significance of paper and the findings in lupus. It is, however, important to determine whether the proposed mechanism is limited to a single autoimmune disease or phenotype.

3. MRL/lpr experiments: One major advantage of the MRL-lpr/lpr strain which makes it highly suitable for modeling human disease is the spontaneous onset of the autoimmune and clinical phenotype. Indeed, the large majority of the lupus relevant studies in this strain involve unmanipulated mice. Moreover, spontaneous arthritis is a feature of MRL-lpr/lpr mouse. It is therefore not clear why the authors chose a CFA-accelerated model in wild type and MMP12 KO mice, and furthermore what are the relevance of any results obtained to spontaneous lupus. Moreover, even in the CFA-triggered experimental setup, central features of this model were not studied or provided (proteinuria, circulating anti-nuclear/anti-DNA autoantibodies). It is recommended that the authors study the spontaneous development of disease in the MMP12 wild type and sufficient MRL/lpr mice, including better characterization of renal disease, circulating antibodies and autoantibodies, spleen/lymph node weight, and survival.

Minor comments:

a. What is the physiological function of MMP12? It is mentioned that it has “established mechanisms in regulation of macrophage activation and to other anti-inflammatory activities”, but what are these? Could any of the results reported be due to other effects of MMP12, besides cleavage of IFNg?

b. What is the phenotype of MMP12 KO mice? Furthermore, is there a difference in IFNg related genes at baseline? (Figure 3A).

c. Is it possible that other IFNs are also being targeted by MMP12? e.g., besides showing that MMP12 doesn't cleave IL-4 (Supp. Figure 5), what is the effect on type I interferon?

d. In some of the in vitro studies, it would be interesting to include an additional control such as LPS which stimulates M1 type macrophages via STAT1, but is independent of IFNg. It is then possible to demonstrate the specificity of MMP12 affecting macrophages via IFNg related pathways.

e. Is it standard practice to demonstrate NETs with H&E staining?

f. The conclusion reached in the figure legend of Supplemental figure 8 about auto-reactive B cells is not justified without at least looking at circulating levels of autoantibodies.

g. Additional detail on the scoring of the kidney histology sections would be beneficial. How many glomeruli were examined? What parameters were scored? Moreover, both H+E and IHC (figure 6i) demonstrate peri-glomerular/interstitital disease as well. Tubulo-interstitial disease should be quantitated separately from glomerular disease, whether or not both were included in the glomerulonephritis score provided in figure 6g.

Reviewer #2 (Remarks to the Author):

This manuscript elegantly demonstrates the importance of IFN-γ regulation during cellular inflammatory response. The authors found an inbuilt negative feedback loop regulating IFN-γ activity that correlates with a decrease in proinflammatory markers. Specifically, they found that macrophage-specific matrix metalloproteinase 12 (MMP12) cleaves IFN-γ, which renders the ligand inactive, highlighting the importance of post-translational cleavage events in the regulation of immune responses. To identify the cleavage sites, the authors used a top-down mass spectrometry approach and identified the C-terminal truncations to occur at 157Met↓Leu158 and 135Glu↓Leu136. To characterize the role of truncated IFN-γ , mouse (RAW264.7) and human (THP-1) macrophages were used, and levels of proinflammatory markers iNOS, ROS, as well as activated and inactivated STAT1 protein levels were monitored after treatment with IFN-γ, MMP12-cleaved IFN-γ, or anti-inflammatory IL-4. This analysis showed that cleaved IFN-γ leads to reduced iNOS, ROS, and phosphorylated STAT1. To test the functional aspect of these results, the authors performed a phagocytosis assay and found reduced phagocytic activity in cells treated with MMP12-cleaved IFN-γ when compared to IFN-γ treated cells. In vivo studies were next used to discover that the loss of MMP12 leads to increased IFN-γ-dependent proinflammatory markers in lupus and arthritis mouse models. Using Mmp12+/+ and Mmp12-/- mice, the authors went on to confirm that the catalytic activity of MMP12 is necessary for the IFN-γ –dependent cellular switch from proinflammatory to immunosuppressive. Importantly, the authors showed the significance of misregulated IFN-γ by demonstrating that reduced MMP12 mRNA levels are correlated with systemic lupus erythematosus clinical deterioration, and also demonstrated increased active IFN-γ in glomeruli of patients suffering from lupus nephritis when compared to healthy individuals. Altogether, the authors present strong evidence supporting the importance of MMP12 in regulating IFN-γ-dependent inflammatory responses and highlight a potential diagnostic and therapeutic path for the future of autoimmune

disease treatment. There are some minor points that require clarification that are outlined below, but overall, this is a valuable contribution to the current understanding of immunological regulation.

Main concerns:

1. Wording and interpretation question: The authors state that IFN-γ cleavage is reduced. This is stated both in the main title of the manuscript, as well as in the title of the section focused on patients with SLE lupus nephritis data. However, this only seems to be proven in this manuscript in in vitro studies. Specifically, Figure 7 illustrates an increase in active IFN-γ (as the authors correctly state in the main text), rather than reduced levels of the cleaved form. The wording of the titles should be adjusted accordingly. The results anyway provide support for the author's hypothesis; the conclusions just need to be stated correctly.

2. Connected to the point above, the antibody raised against the N-terminus region of IFN-γ should detect all three IFN-γ forms mentioned – the full length and active and the two cleaved and inactive forms. Have the authors tried to resolve full length from the 2nd cut form by western blotting or other biochemical separations using the generated antibodies? This would provide further support for their conclusion. It is understood that the amount of patient tissue samples may not be sufficient for such studies. Have the authors tried to do this in mouse models? Is this what the authors try to show with the arrows in Figure 6e? These are not explained in the text, and the background of the blot seems brightened.

3. Missing loading controls should be added:

a. Loading controls are necessary for Fig4a and c for the 15min samples, as well as for Fig4b and d (loading control under iNOS).

b. As the total protein levels for STAT1 fluctuate, it would help to add an additional standard loading control for fig8 a, b, d and e.

Minor concerns:

1. Fig2a – the authors should clarify why the MMP12 levels go down when IFN-γ is cleaved (lanes 4 and 5).

2. Fig3a needs an x axis label (days)

3. Fig 4f - Since this experiment nicely displays a function related to active vs. inactive IFN-γ, some additional information would be helpful to clarify the measurement of the phagocytic index. The authors mention that they quantified the number of beads per cell. How many cells were used, as how were the microscopy images acquired and processed for the quantification? Does the description of n=3; N=2 refer to 3 biological replicates in technical duplicates, and is this representative of both panels e and f in this figure?

4. Fig7a the right part of the middle panel of the western blot looks washed out. Do the authors have another blot or exposure they can use?

5. Fig 7a – it seems like the antibody against C-terminal-2 would be able to recognize both the full-length and the form cleaved at 1st cut. The authors should reword the description in the main text.

Reviewer #3 (Remarks to the Author):

The manuscript by Dufour et al provides data supporting the authors' hypothesis that MMP12-mediated processing of interferon-gamma (IFNg) is a method for dampening the inflammatory response, and in the absence of such proteolytic processing, a chronic pro-inflammatory response is seen. Moreover, the authors provide evidence that the MMP12/IFNg axis is altered in autoimmune disease such as systemic lupus erythromous (SLE). Overall, this is a well-conducted study with multiple experimental findings that reinforce each other and support the hypothesis. The findings are of interest particularly with respect to elucidating control mechanisms for different phases of inflammation. However, there are some issues with the choice of models, and with some of the descriptions of animals.

The data showing processing of IFNg by MMP12 and the resulting loss of IFNg-signaling and downstream effects in vitro and in the acute inflammatory peritonitis model in vivo are robust and strongly supportive of the central idea. However, an acute peritonitis (or in vitro treatment of "macrophage" cell lines) is not the same as a chronic lymphoid-driven disease such as SLE. It is puzzling that the MMP12+/+ and MMP12-/- MRL/lpr mice appear to be similar with respect to lymph node size at baseline, which here is 90 days old. At this age, MRL/lpr mice generally show a demonstrable lymphoproliferative phenotype, which is the basis for the disease, thus the lack of difference suggests that MMP12-mediated processing of IFNg is not causal. The fact that differences are seen only after administration of adjuvant to induce an inflammatory response, reinforces the idea that the MMP12/IFNg axis is more critical for acute inflammatory events rather than chronic autoimmune disorders, although there may be an important role in disease flares. This should be discussed. Currently in the discussion, there is an implication for mechanistic contribution of IFNg to disease in the MRL/lpr models that the authors acknowledge cannot be extrapolated yet to the human disease. However, even in the mouse models, the authors have not done a rescue experiment of adoptively transferring MMP12+/+ macrophages into the MMP12-/- MRL/lpr mice to show requirement for this axis in disease progression.

In terms of the description of the mouse models, the genetic backgrounds are not very clear. For example are the MMP12-/- mice backcrossed to C57bl/6 background after having been generated with 129SvEV ES cells? Or are they a true 129XC57 mixed background? This has important implications for controls, especially as C57bl/6 mice are considered a “low inflammation” strain. Following on from this, are MRL/lpr MMP12+/+ mice generated in the same way (by backcrossing from a similar starting strain) or are they just MRL/lpr mice? Similar questions apply to the crossing of 129SvEV MMP12-/- onto the B10.RIII mice.

On additional question regarding the alterations in macrophage polarization observed in the presence and absence of MMP12 in vivo: are the total number of macrophages in the tissue similar, as well as the total number of circulating monocytes?

Minor Point:

In the summary/abstract, the term ‘macrophage-specific’ is used to refer to MMP12. Since other cell types can express this protease, the term ‘macrophage-associated’ is more correct.

REVIEWER 1

The authors made a significant effort to address the comments on the initial review of the manuscript, and have substantially responded to most of them in a satisfactory matter.

However, several important original comments were not appropriately addressed. The MRL/lpr spontaneous lupus model is central in this paper, yet is not properly characterized. Besides lymphadenopathy, the key features of lupus in this strain which should be evaluated here are kidney disease and anti-double stranded DNA antibodies. The standard evaluation of nephritis in lupus is not by kidney weight (Fig 6f), or MHC/IFN-gamma staining (Fig. 6h, i), but rather via quantification of kidney function (urine protein, blood urea nitrogen) and the severity of the glomerulonephritis.

Urine may not have been collected, but is serum still available to measure blood urea nitrogen with commercially available kits?

While a glomerulonephritis score is provided (Fig. 6g), Dufour et al appear to have confused histological class (which is a morphological pattern, and not linear class I through VI; moreover MRL/lpr mice have proliferative disease, rather than membranous (class V) histology) with the usual scoring of activity and chronicity (e.g. mesangial proliferation, crescent formation, IgG deposits, interstitial infiltrates, tubular atrophy, etc). See for example Renner et al Kid Int 2015; Jiang et al Arth Rheum 2011, and many other papers studying this strain.

Finally, anti-nuclear humoral autoimmunity is a central feature of murine (and human) lupus, and measurement of serum anti-DNA antibodies can be completed over several hours.

Response: Done as suggested. The severity of glomerulonephritis pathology was further evaluated. Each biopsy specimen was assessed for 10 components, of which 6 make up the *Renal Activity Index* (***new* Fig. 6g left**) and 4 the *Chronicity Activity Index* (***new* Fig. 6g right**) as described in the suggested papers (Renner *et al*. (2015)*;* Jiang et al. (2011)*;* and Austin 3rd *et al.* (1984) *Kidney International* **25**: 689-695; Wernick *et al.* (1993) *Annals Internal Medicine* **119**: 805-811).

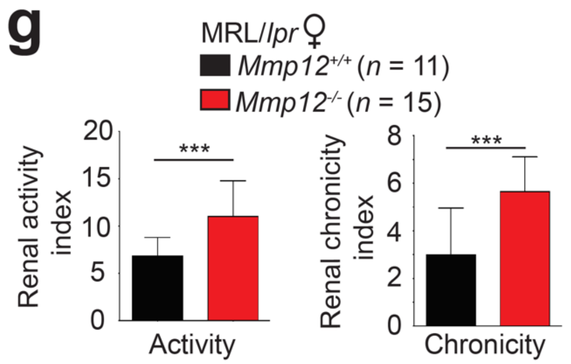

Thus, *the Renal Activity Index* is derived from:
Glomerular abnormalities:
1- cellular proliferation
2- leukocyte infiltration
3- fibrinoid necrosis or karyorrhexis
4- cellular crescents
5- hyaline thrombi, wire loops
Tubulointerstitial abnormalities:
6- mononuclear cell infiltration

The *Chronicity Activity Index* is derived from:
Glomerular abnormalities
1- glomerular sclerosis 2- fibrous crescents
Tubulointerstitial abnormalities:
3- interstitial fibrosis 4- tubular atrophy

Each component was scored 0 (for normal), 1, 2 or 3 (for severe abnormality, 3 being the worst). In calculating the activity index, fibrinoid necrosis and cellular crescents were weighted by a factor of 2. The renal activity index scale ranges from 0 – 24 points and the renal chronicity index scale ranges form 0 – 12. *New* **Fig. 6g** shows that the histological scores of Renal ($p < 3 \times 10^{-4}$) and Chronicity ($p < 7 \times 10^{-4}$) Activity Indexes are significantly elevated in $Mmp12^{-/-}$ MRL/*lpr* female mice (n = 15) in comparison to $Mmp12^{+/+}$ MRL/*lpr* littermates (n = 11). Thus, the Reviewer was correct and in fact the new indices confirm and further strengthen our findings.

Response: Unfortunately, there is no serum or urine remaining from our previous mouse studies collected during disease development to measure blood urea nitrogen or anti-DNA antibodies. The mice do not breed well and we had collapsed our colony to a few maintenance breeders ~3 years ago. To collect serum would require at least three – four months of breeding to expand the colony, waiting 90 days before CFA injection and then following the mice over 28 days. Seven to eight months will easily pass before we could provide these analyses. Thus, after thoughtful consideration of the practicalities, we have elected not to perform these readouts.

The important question is: *What would this data add to the regulation of IFN-y by MMP12 that warrants 7+ months delay?*

As our paper focuses on the proinflammatory signatures in these mice rather than characterization of autoimmune kidney functions, with respect, we suggest that on balance, further technical readouts will provide little new insight on the role of MMP12 in IFN-y function.

Finally, and as suggested by the Reviewers at first revision, rather than emphasizing autoimmune disease, we refocussed the paper on acute inflammation and inflammatory flares. Indeed, our original conclusion to this section did not mention autoimmunity, and on page 9 read: "Overall, in the MRL/*lpr* mice, *Mmp12*$^{-/-}$ mice exhibited sustained IFN-γ signaling favoring ongoing proinflammatory responses *versus* the *Mmp12*$^{+/+}$ MRL/*lpr* mice, which exhibited normal macrophage phenotypic succession to alternatively activated CD36+ macrophages over time". Indeed, Reviewer 3 now pointed out: "Resetting the mechanism from the point of view of inflammatory flares fits well with the current data..."

Therefore, with respect, adding anti-DNA antibody levels might not provide meaningful new insight over the extensive pathological evaluation now done as suggested by the Reviewer, particularly with the emphasis now on acute inflammatory flares.

Nonetheless, as the MRL/lpr autoimmune lupus-like mouse model aligns closely with what is seen in humans **Supplementary figure 11** reports **anti-double stranded DNA antibody levels in 5 lupus nephritis patients**, which is more relevant, and several other factors including glomerular filtration rate (which both our pathologists (Jacob Rozmus/Sean Barbour) consider a better measure than BUN), creatinine, albumin/creatinine ratio, 24 h proteinuria, erythrocyte sedimentation rate, C-reactive protein, C3, C4 and as well as MMP12 and IFN-γ protein levels.

We thank all reviewers for their excellent insights and constructive comments, which by taking them seriously, have led to a much-improved manuscript.
